# RNA virus diversity in three parasitoid wasps of tephritid flies: insights from novel and known species

**DOI:** 10.1128/spectrum.03139-23

**Published:** 2023-11-06

**Authors:** Wei Zhang, Rong Li, Shuai Li, Shao-Yang Li, Jinzhi Niu, Jin-Jun Wang

**Affiliations:** 1 Key Laboratory of Entomology and Pest Control Engineering, College of Plant Protection, Southwest University, Chongqing, China; 2 Key Laboratory of Agricultural Biosafety and Green Production of Upper Yangtze River (Ministry of Education), International Joint Laboratory of China-Belgium on Sustainable Crop Pest Control, Academy of Agricultural Sciences, Southwest University, Chongqing, China; Fujian Agriculture and Forestry University, Fuzhou, Fujian, China

**Keywords:** parasitoid wasps, tephritid flies, RNA virus, VsiRNA

## Abstract

Parasitoid wasps are a diverse group of parasitoid insects that play a crucial role in biological control and integrated pest management programs due to their wide range of host species and complex behaviors. Previous studies have reported that select RNA viruses in parasitoid wasps can affect either the host-parasitoid wasp or the pest host parasitized by the wasps. Therefore, a study of the dynamics of RNA viruses in parasitoid wasps is essential for artificial breeding programs and improving their parasitic capabilities. In this study, we detected the presence of nine novel and three known RNA viruses with complete genomes in three parasitoid wasp species: *Diachasmimorpha longicaudata*, *Fopius arisanus*, and *Spalangia endius*. These wasps are parasitoids that can parasitel tephritid flies, one of the most important pest groups of fruits and vegetables. In *D. longicaudata*, the small RNAs derived from the viruses exhibited distinct peaks at 22 nt and were symmetrically distributed across the viral genome. These findings indicate that these viruses can activate the host’s RNAi immune response. PCR detection further revealed that DlNaLV efficiently infects female individuals of *D. longicaudata* with 100% efficiency, and ZcNLV is capable of infecting both *D. longicaudata* and its host, *Zeugodacus cucurbitae*. Our findings contribute to a better understanding of RNA viruses in parasitoid wasps and lay the foundation for future research on the triple-level trophic relationships between tephritid flies, parasitoid wasps, and RNA viruses.

**IMPORTANCE:**

Parasitoid wasp populations have developed persistent beneficial symbiotic relationships with several viruses through repeated evolution. However, there have been limited reports on RNA viruses in parasitoid wasps of tephritid flies, a significant pest group affecting fruits and vegetables. This study explores the diversity of RNA viruses in three parasitoid wasps of tephritid flies and highlights the potential biological significance of specific viruses in *Diachasmimorpha longicaudata*. These findings have important implications for the development of sustainable pest management strategies and the enhancement of artificial rearing techniques for parasitoid wasps.

## INTRODUCTION

Biological control, which is a green, environmentally friendly, safe, and sustainable approach, can effectively manage pest populations in agricultural ecosystems (1). This method utilizes natural enemies, biological secretions, and pathogens as alternatives to reduce excessive reliance on chemical pesticides (2). Among these, the field release of parasitoid wasps constitutes an essential strategy for pest biological control. Despite its longer duration and potentially higher costs, this method is environmentally friendly and avoids the issues associated with pesticide toxicity, residue, or resistance (3). Furthermore, once released artificially, these organisms often undergo natural reproduction and spread, resulting in the establishment of stable populations within the ecosystem.

Viruses play a crucial role in the parasitism of parasitoid wasps, co-evolving with them to establish a mutually beneficial symbiotic relationship characterized by persistent infection (4). The normal growth and development of parasitoid wasp offspring within the host organism depend on the virulence exerted by the virus, while the virus relies on the reproductive behaviors of the parasitoid wasp for transmission and dissemination. It has been reported that some viruses have even integrated into the genome of parasitoid wasps, forming endogenous virus elements (EVEs). Polydnaviruses (PDVs) represent extreme cases of beneficial EVEs, as parasitoid wasps harbor complete machinery of complex DNA viruses in their genomes, enabling the transfer of virulence genes into parasitized hosts and ensuring the success of wasp parasitism (5). Additionally, some DNA and RNA viruses have also been confirmed to be associated with the parasitism of parasitoid wasps. For example, the *Diachasmimorpha longicaudata* entomopoxvirus (DlEPV) disrupts the regular functions of the host’s hemocytes, ensuring the normal growth and development of offspring of the parasitoid wasp *D. longicaudata* within its host (6, 7). Infection with *Pteromalus puparum* negative-strand RNA virus-1 (PpNSRV-1) has been observed to extend the lifespan of its host, *P. puparum*, and significantly reduce the number of female progenies, thereby modulating the offspring sex ratio (8). Rondani’s wasp virus 1 (RoWV-1) infects the host *Drosophila melanogaster*, which is parasitized by the parasitoid wasp *Pachycrepoideus vindemmiae*, and it exhibits extensive proliferation within the fruit fly. Furthermore, RoWV-1 enhances the oviposition capacity and prolongs the developmental duration of *D. melanogaster*, thereby ensuring that the parasitoid wasps have a higher number of hosts for successful reproduction of offspring (9).

Recent advancements in viral metagenomics and high-throughput sequencing technologies have significantly facilitated the discovery of invertebrate RNA viruses (10 – 12), accelerating research on the complex interplay between parasitoid wasps and their associated viruses. Furthermore, these technological advancements have not only enabled the identification of new invertebrate RNA viruses but have also allowed for a deeper understanding of the intricate dynamics and interactions between parasitoid wasps and their associated viruses. As scientists continue to delve into the fascinating realm of viral metagenomics and utilize advanced sequencing techniques, our knowledge of the co-evolution, transmission, and impact of these viruses on parasitoid wasp ecology and behavior will undoubtedly expand. This knowledge, in turn, can contribute to the development of novel strategies for biological control and the sustainable management of pest populations in agricultural ecosystems.

Tephritid flies comprise a highly invasive and detrimental group of pests, encompassing over 500 genera and 5,000 species (13). When introduced to a new region with favorable environmental conditions, they undergo rapid proliferation and spread, leading to devastating impacts on local fruit and vegetable industries (14). Three parasitoid species, namely *D. longicaudata*, *Fopius arisanus*, and *Spalangia endius*, show great potential for biologically controlling tephritid flies by parasitizing their larvae or pupae in their natural habitats. For a comprehensive understanding of the relationship between these parasitoid wasps and their hosts, we employed a meta-transcriptomic approach to characterize the RNA viromes within these parasitoid wasps. This study will provide valuable insights and a reference for investigating the interactions between parasitoid wasps and their host tephritid flies, as well as optimizing the utilization of parasitoid wasps in the biological control of tephritid pests.

## RESULTS

### RNA virome of three parasitoid wasps

To identify potential viral sequences in three parasitoid wasps, we constructed an RNA-Seq library (accession number: SRR25621800) for *D. longicaudata*. Additionally, RNA-seq libraries were downloaded for *S. endius* and *F. arisanus*. Following quality control, we obtained 44,117,406 paired clean reads from the RNA-seq library of *D. longicaudata*. Utilizing *de novo* assembly fragments, we searched non-redundant protein databases and identified potential viral sequences based on NR annotation results. Our analysis identified 12 potential viral sequences, with no cross-species phenomenon observed. Among these, nine showed less than 85% amino acid sequence identity with the known viral sequences, implying that they potentially represent novel viral sequences (Table 1). Furthermore, three potential viral sequences exhibited over 85% amino acid sequence identity with the known viral sequences, indicating that they may represent the known viral sequences (Table 2). In total, we identified three novel and one known RNA viral sequence from *D. longicaudata*, two novel and one RNA viral sequence from *S. endius*, and four novel and one known RNA viral sequence from *F. arisanus* (Tables 1 and 2).

**TABLE 1:** Putative novel RNA virus-derived sequences were identified in three parasitoid wasps

Virus name	Host	Length	Genome	Family	Closest relative species	Percent identity
*Diachasmimorpha longicaudata* tombus-like virus	*D. longicaudata*	2,024 nt	ssRNA (+)	*Tombusviridae*	Megalopteran tombus-related virus	51.53%
*Diachasmimorpha longicaudata* narna-like virus	*D. longicaudata*	3,345 nt	ssRNA (+)	*Narnaviridae*	Sanya narnavirus 2	44.42%
*Diachasmimorpha longicaudata* rhabdo-like virus	*D. longicaudata*	11,938 nt	ssRNA (−)	*Rhabdoviridae*	Hymenopteran rhabdo-related virus	43.85%
*Spalangia endius* iflavirus	*S. endius*	6,282 nt	ssRNA (+)	*Iflaviridae*	Tetrastichus brontispae RNA virus 3	45.66%
*Spalangia endius* hurwu-like virus	*S. endius*	Segment 1: 8,880 nt	ssRNA (−)	*Phenuiviridae*	Solenopsis invicta virus 14	45.01%
		Segment 2: 1,786 nt				
		Segment 3: 1,180 nt				
*Fopius arisanus* dicistrovirus	*F. arisanus*	8,295 nt	ssRNA (+)	*Dicistroviridae*	Black queen cell virus	47.41%
*Fopius arisanus* Nora virus	*F. arisanus*	11,881 nt	ssRNA (+)	Undefined	Nora virus	36.51%
*Fopius arisanus* permutotetra-like virus	*F. arisanus*	4,953 nt	ssRNA (+)	*Permutotetraviridae*	Inari permutotetravirus	48.93%
*Fopius arisanus* narna-like virus	*F. arisanus*	3,327 nt	ssRNA (+)	*Narnaviridae*	Hubei narna-like virus 21	44.62%

**TABLE 2:** Three known RNA viruses in three parasitoid wasps

Virus name	Host	Length	GenBank accession no.	Query cover	E value	Percent identity
*Zeugodacus cucurbitae* negev-like virus	*D. longicaudata*	10,107 nt	MW310350.1	99%	0.0	99.92%
*Vespa velutina* associated acypi-like virus	*S. endius*	6,875 nt	OK491517.1	99%	0.0	90.75%
*Zeugodacus cucurbitae* dicistrovirus	*F. arisanus*	8,621 nt	MW310349.1	98%	0.0	88.96%

### Novel positive-sense RNA viruses

**Fig 1:**
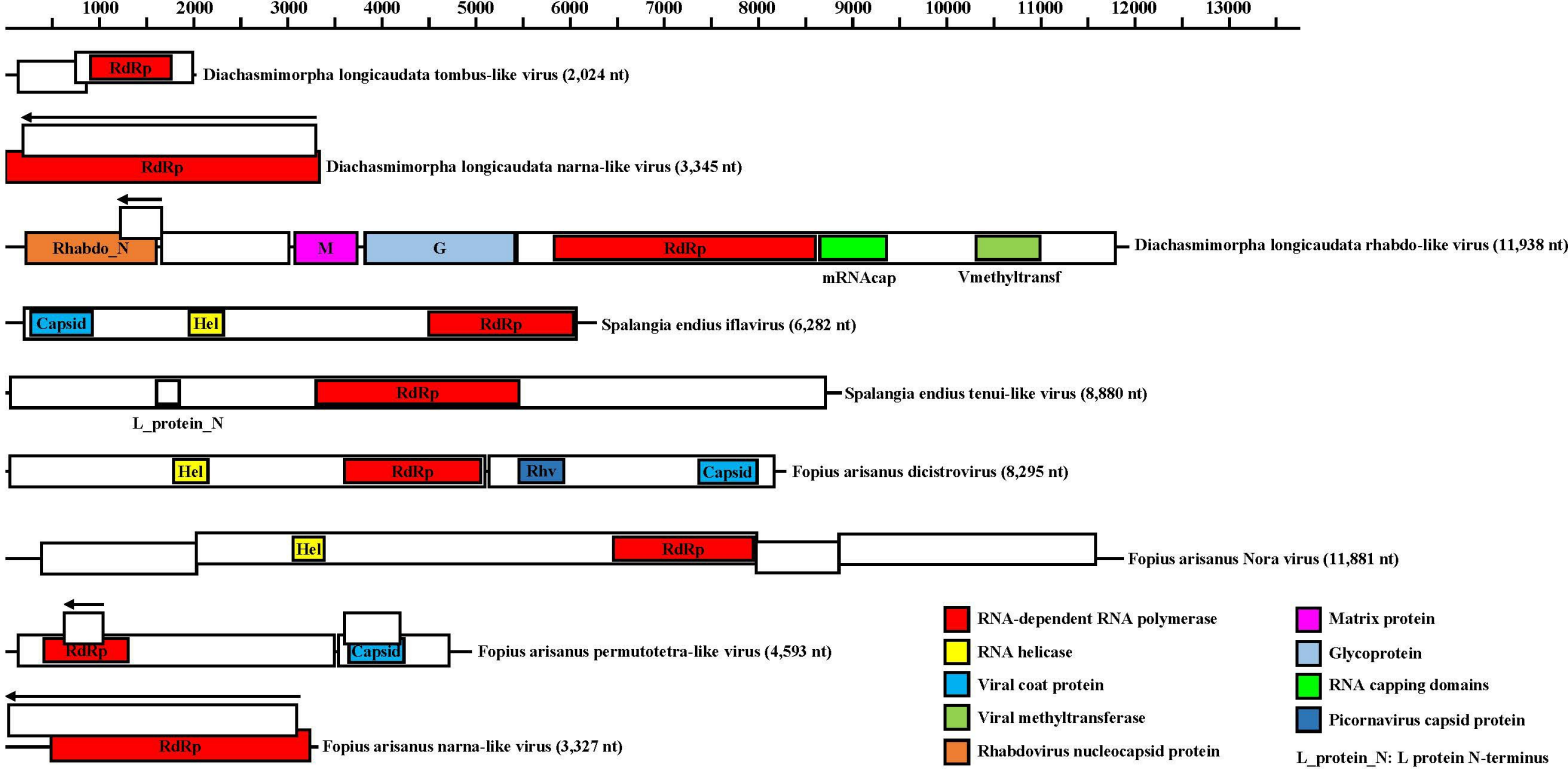
Schematic representation of the viral genomic structure of nine novel RNA viruses in three parasitoid wasps. White rectangles represent ORFs and black arrows represent the direction of the reverse ORFs translation. The conserved domains were shown as rectangles and marked with different colors in ORFs.

**Fig 2:**
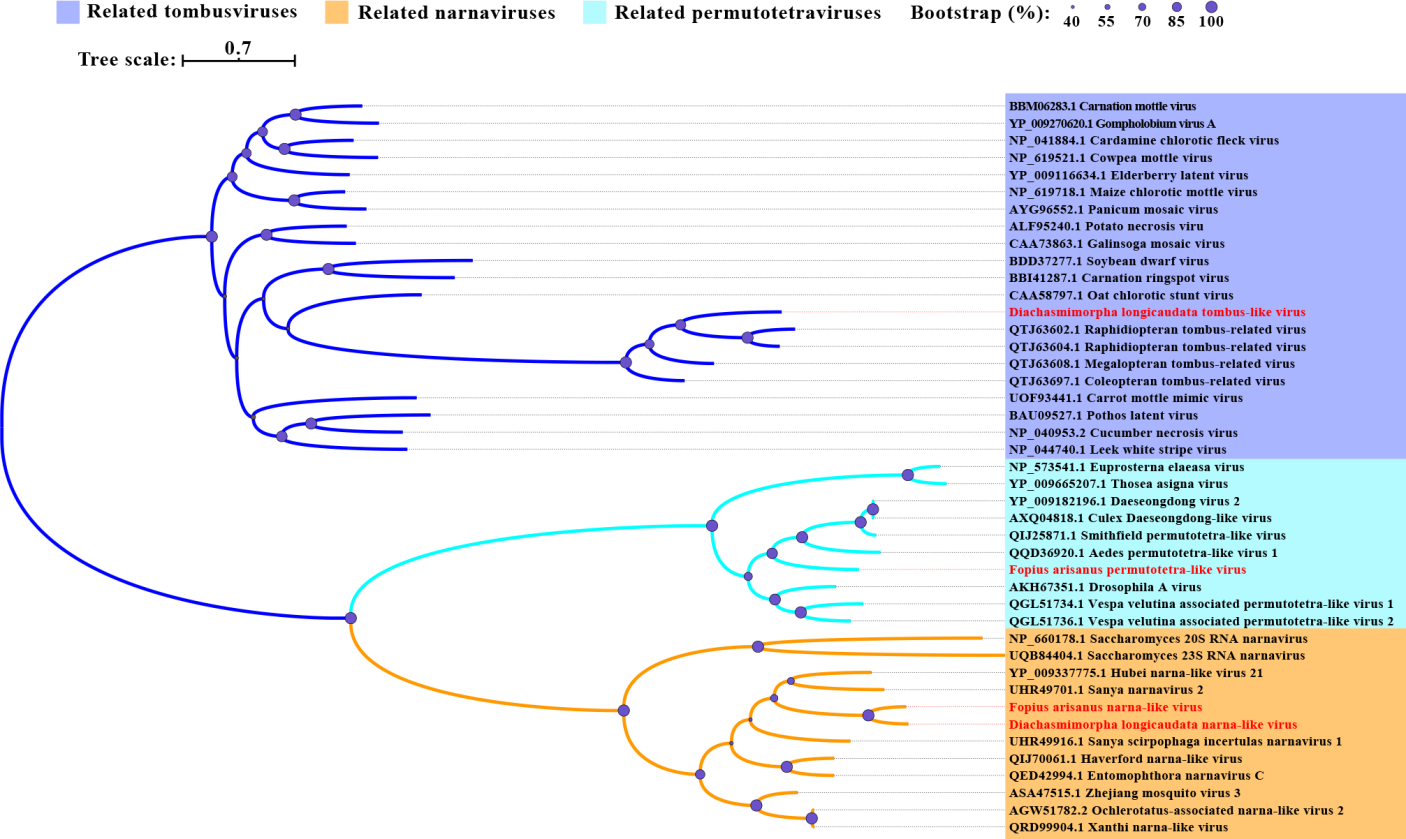
Phylogenetic analysis of DlTLV, FaPLV, DlNaLV, and FaNaLV. Phylogenetic tree of picornaviruses based on the deduced amino acid sequences of RdRp domains. On the individual branches, purple circles represented bootstrap support from 1,000 bootstrap replicates with a larger circle size indicating greater support.

We discovered seven positive-sense RNA virus sequences in three parasitoid wasps. In *D. longicaudata*, we identified and named a novel tombusvirus and a novel narnavirus as *D. longicaudata* tombus-like virus (DlTLV) and *D. longicaudata* narna-like virus (DlNaLV), respectively. The genome of DlTLV consisted of 2,024-nt and contained two partially overlapping open reading frames (ORFs). ORF1 (239 aa) encoded a hypothetical protein, while ORF2 (414 aa) encoded RNA-dependent RNA polymerase (RdRp) (Fig. 1). The RdRp of DlTLV exhibited the highest genetic identity (51.53%) to the Megalopteran tombus-related virus (Table 1). Phylogenetic analysis revealed that DlTLV clustered with Raphidiopteran tombus-related viruses, Megalopteran tombus-related viruses, and Coleopteran tombus-related viruses (Fig. 2). On the other hand, the positive sense genome of DlNaLV had a length of 3,349-nt and included two forward ORFs and one reverse ORF. Forward ORF1 (1,115 aa) encoded RdRp, forward ORF2 (194 aa) encoded an unknown protein, and the reverse ORF (1,036 aa) encoded a hypothetical protein (Fig. 1). The RNA-dependent RNA polymerase of DlNaLV exhibited a high genetic identity (44.42%) to Sanya narnavirus 2 (Table 1). Phylogenetic analysis suggested that DlNaLV belonged to the Narnavirus-related clade (Fig. 2).

**Fig 3:**
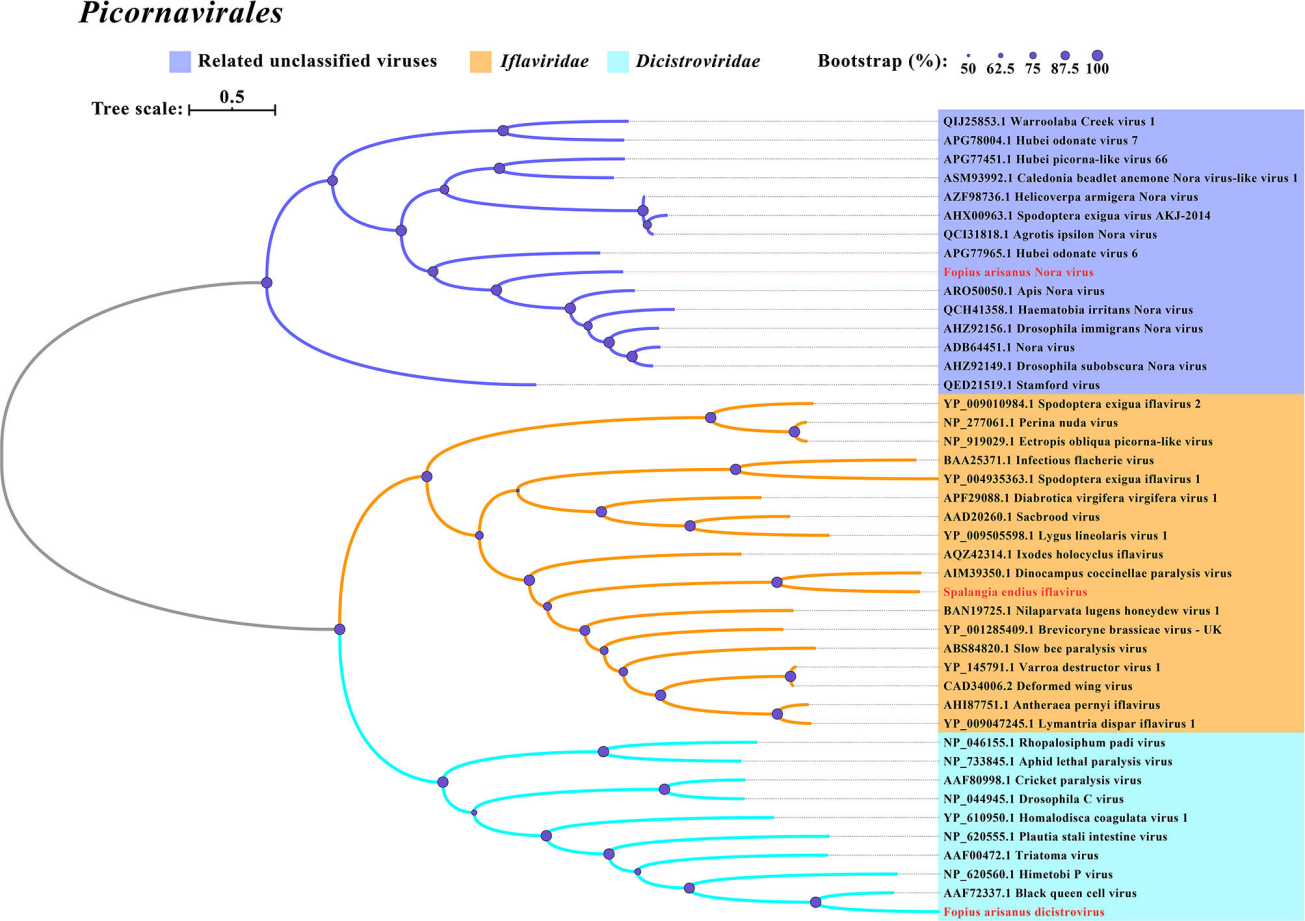
Phylogenetic analysis of picornaviral viruses discovered in the three parasitoid wasps. The other legends are the same as those used in Fig. 2.

Four novel positive-sense RNA viruses were discovered in *F. arisanus* and given the names *Fopius arisanus* permutotetra-like virus (FaPLV), *F. arisanus* dicistrovirus (FaDV), *F. arisanus* Nora virus (FaNV), and *F. arisanus* narna-like virus (FaNaLV), respectively. The genome sequence of FaPLV is 4,953-nt and includes three forward ORFs and one reverse ORF. Forward ORF1 (1,119 aa) encodes the RdRp protein, forward ORF2 (391 aa) encodes the capsid protein, while forward ORF3 (196 aa) and reverse ORF (138 aa) encode unknown proteins (Fig. 1). The RdRp of FaPLV showed the highest genetic similarity (48.93%) to the Inari permutotetravirus (Table 1). Phylogenetic analysis placed FaPLV in a clade with the Smithfield permutotetra-like virus and Aedes permutotetra-like virus (Fig. 2). The genome sequence of FaDV is 8,295-nt and consists of two ORFs. ORF1 (1,682 aa) encodes helicase (Hel) and RdRp, while ORF2 (1,010 aa) encodes two capsid proteins: Picornavirus capsid protein (Rhv) and Capsid (Fig. 1). Sequence alignment analysis revealed that FaDV had the highest similarity (47.41%) to Black queen cell virus (Table 1). Phylogenetic analysis also showed that FaDV was most closely related to Black queen cell virus (Fig. 2). The genome sequence of FaNV is 11,881-nt long and includes four ORFs. ORF2 encodes Hel and RdRp proteins, while ORF1, ORF3, and ORF4 encode hypothetical proteins (Fig. 1). The RdRp of FaNV showed a high genetic identity (36.51%) to Nora virus (Table 1). Phylogenetic analysis indicated that FaNV belonged to the Nora virus-related clade (Fig. 3). The genome of FaNaLV is 3,327-nt and contains one forward ORF and one reverse ORF. The forward ORF (916 aa) encodes the RdRp protein, while the reverse ORF (1,020 aa) encodes a hypothetical protein (Fig. 1). Sequence analysis based on RdRp revealed that FaNaLV had the highest similarity to Hubei narna-like virus 21, with a similarity of 44.62% (Table 1). The phylogenetic tree indicated the closest relationship between FaNaLV and DlNaLV (Fig. 2).

A new iflavirus, named Spalangia endius iflavirus (SeIV), was discovered in *S. endius*. SeIV has a genome of 6,282-nt, which contains a single open reading frame (ORF) encoding Capsid, Hel, and RdRp (Fig. 1). Sequence analysis demonstrated that this virus exhibits the highest similarity to Tetrastichus brontispae RNA virus 3. The phylogenetic tree indicated that SeIV clusters together with other iflaviruses (Fig. 3).

### Novel negative-sense RNA viruses

**Fig 4:**
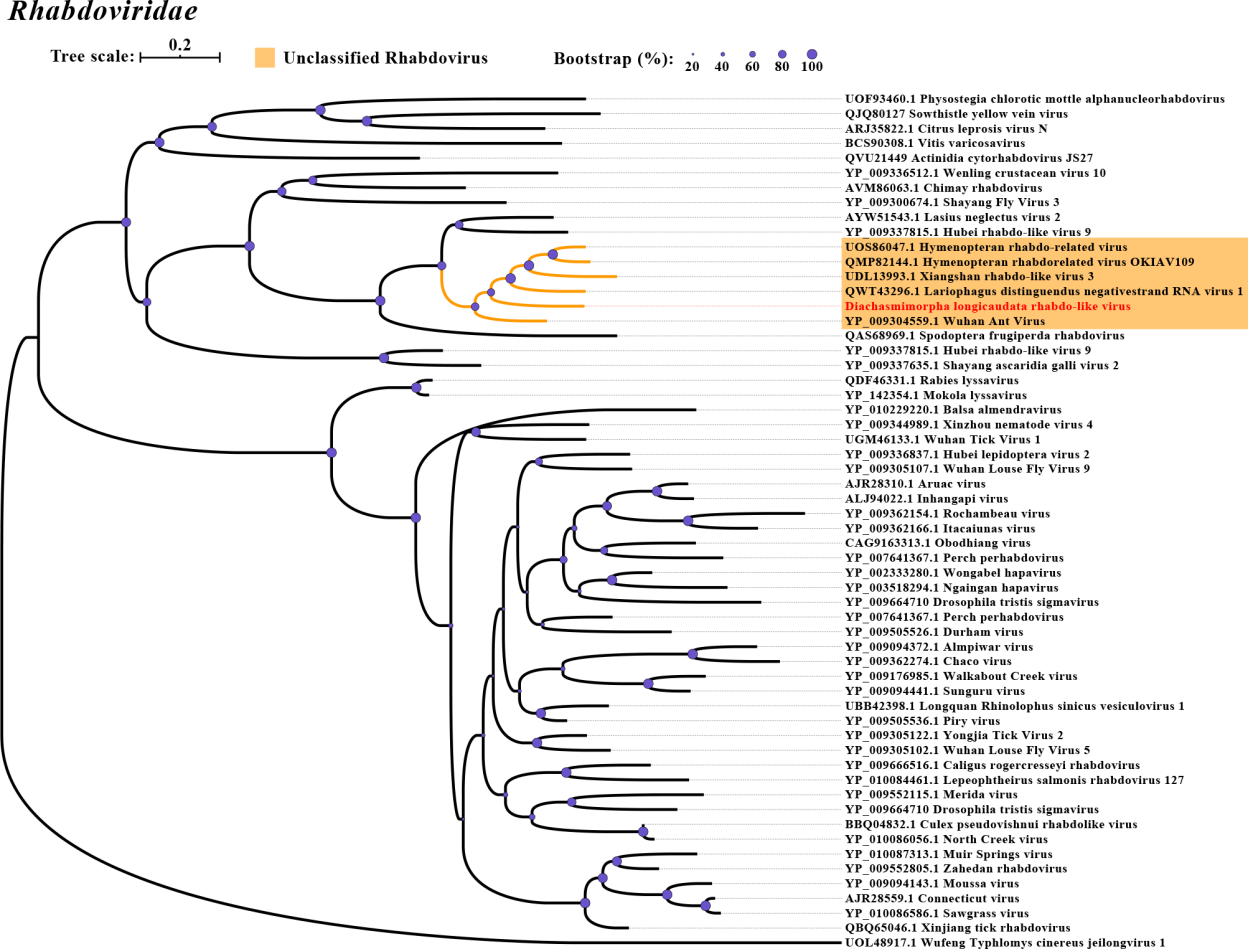
Phylogenetic tree of *Diachasmimorpha longicaudata* rhabdo-like virus. The other legends are the same as those used in Fig. 2.

**Fig 5:**
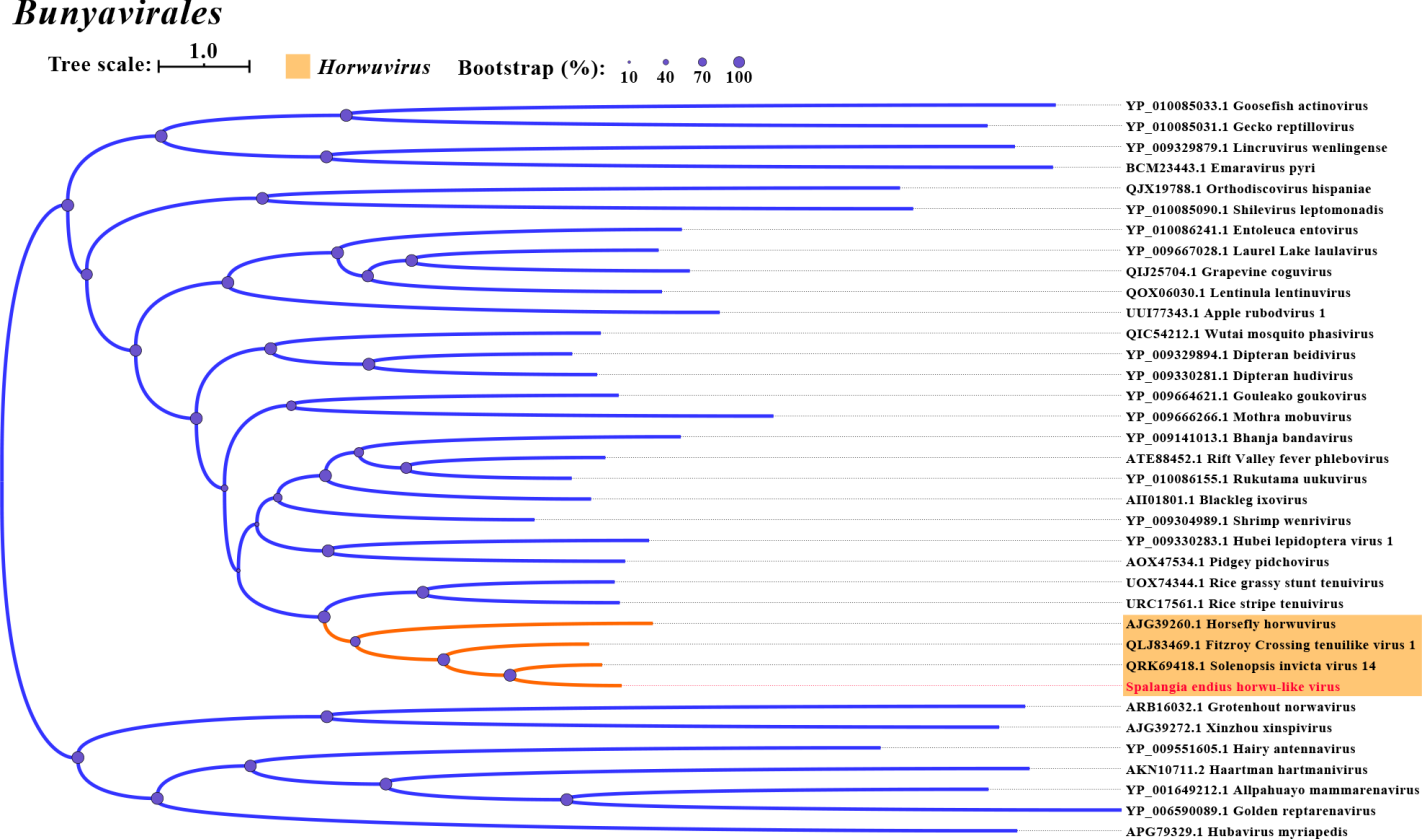
Phylogenetic tree of *Spalangia endius* horwu-like virus 1. The other legends are the same as those used in Fig. 2.

In this study, we identified two negative-sense RNA viruses in *D. longicaudata* and *S. endius*, named *D. longicaudata* rhabdo-like virus (DlRLV) and Spalangia endius hurwu-like virus (SeHLV), respectively. The assembled genome of DlRLV was 11,938 nt, consisting of five forward ORFs and one reverse ORF. ORF1 (460 aa) encoded a Rhabdovirus nucleocapsid protein, ORF2 (449 aa) encoded a hypothetical protein, ORF3 (219 aa) encoded the Matrix protein, ORF4 (529 aa) encoded the Glycoprotein, and ORF5 (2,117 aa) encoded RdRP, RNA capping domain, and Viral methyltransferase. The reverse ORF encoded an unknown protein (Fig. 1). The RdRp of DlRLV showed the highest genetic identity (43.85%) with the Hymenopteran rhabdo-related virus (Table 1). Phylogenetic analysis revealed that DlRLV belongs to a clade containing unclassified Rhabdoviruses, including Hymenopteran rhabdo-related virus, Xiangshan rhabdo-related virus 3, Lariophagus distinguendus negative-strand RNA virus 1, and Wuhan Ant Virus (Fig. 4). The genome of SeHLV consisted of three segments: Segment 1 (8,880 nt), Segment 2 (1,786 nt), and Segment 3 (1,180 nt). Segment 1 contained one ORF (2,885 aa) encoding the L protein N-terminus and RdRp. Segment 2 consisted of one forward ORF (262 aa) encoding the *Tenuivirus* NS4 protein and one reverse ORF (175 aa) encoding the *Tenuivirus* NCP protein. Segment 3 consisted of one ORF (308 aa) encoding the *Tenuivirus* nucleocapsid protein (Fig. 1). Sequence analysis based on RdRp revealed that SeHLV displayed the highest similarity (45.01%) with Solenopsis invicta virus 14 (Table 1). Phylogenetic analysis also revealed that SeHLV was phylogenetically closest to Solenopsis invicta virus 14 and belonged to the *Horwuvirus* (Fig. 5).

### Virus-derived small RNAs in *D. longicaudata*

**Fig 6:**
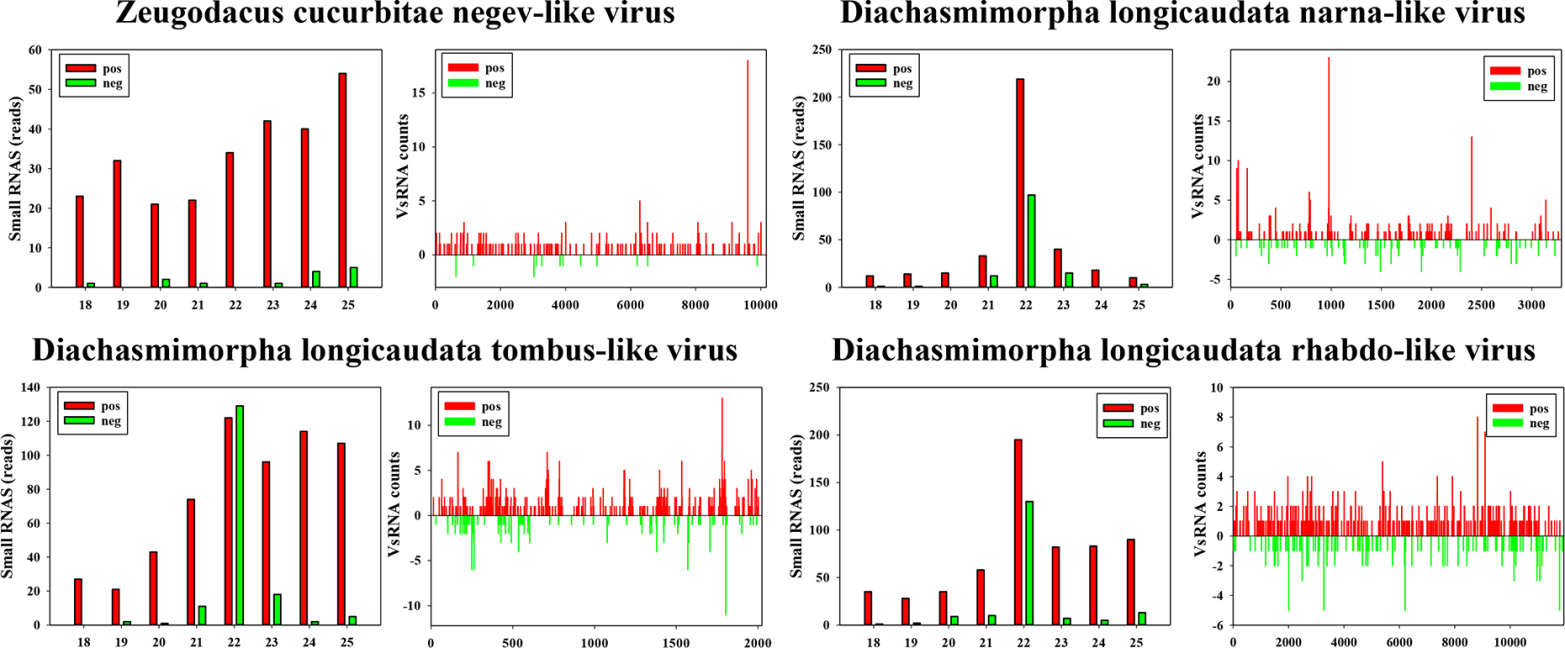
Distribution of small RNAs derived from four RNA viruses in *Diachasmimorpha longicaudata*. Size distribution analysis and schematic representation distribution of RNA virus-derived VsRNAs, red bars represent the VsRNAs from the positive genome, while the green bar represents the VsRNAs from the negative strand.

In the context of RNAi antiviral immunity, viral double-stranded RNA structures can induce host RNAi responses and produce virus-derived small RNAs (VsRNAs). These VsRNAs then specifically silence viral gene expression by targeting the viral genome (15). To determine whether these viruses truly represent infections rather than being contaminants from food or surfaces, we constructed a small RNA library and analyzed the distribution characteristics of VsRNAs derived from four viruses in *D. longicaudata*. The sRNA library yielded a total of 32,801,219 clean reads (accession number: SRR25621798). DlNaLV, DlTLV, and DlRLV exhibited robust small RNA signals, characterized by a prominent peak at 22-nt, and a symmetrical distribution of VsRNAs across both strands of the genome (Fig. 6). In contrast, the *Zeugodacus cucurbitae* negev-like virus (ZcNLV) showed a robust small RNA signal on the positive strand of the genome, but it did not display a prominent peak at 22 nt. To gain further insight into the small RNA distribution of ZcNLV, we performed a reconstruction of the small RNA library (accession number: SRR25621799) and conducted a thorough analysis of ZcNLV’s small RNA data. The results showed that the VsRNAs derived from ZcNLV displayed a minor peak at 22 nt (Fig. S1).

### Abundance of viruses in *D. longicaudata*

**Fig 7:**
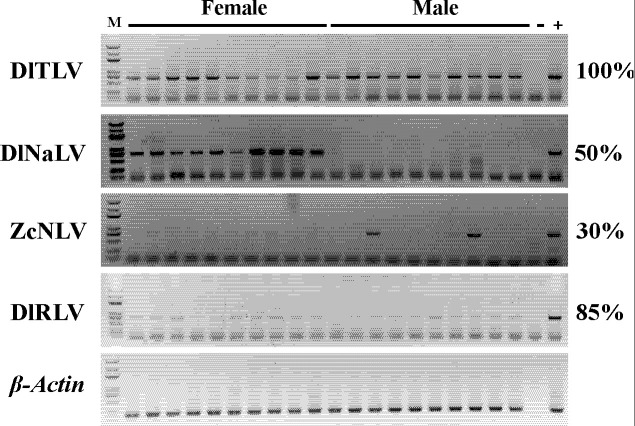
Detection of virus-derived sequences from putative four RNA viruses in *Diachasmimorpha longicaudata* by RT-PCR. DlTLV, *Diachasmimorpha longicaudata* tombus-like virus; DlNaLV, *Diachasmimorpha longicaudata* narna-like virus; ZcNLV, *Zeugodacus cucurbitae* negev-like virus; DlRLV, *Diachasmimorpha longicaudata* rhabdo-like virus; *β-Actin*, reference gene of *D. longicaudata*; Female, female adults; Male, male adults; M, maker 5000; + , positive control; − , negative control.

To determine the prevalence level of each virus species associated with *D. longicaudata*, we conducted RT-PCR analysis on 10-female and 10-male-adult specimens. The reference gene test results confirmed that the samples were of high quality and free from any quality issues. Overall, all four viruses were found to be present in the *D. longicaudata* populations (Fig. 7). Specifically, DlTLV showed a 100% infection frequency with a distinct electrophoretic band, while DlNaLV exclusively infected females with a 100% infection rate. Infection frequencies of 30% and 80% were observed in both female and male adults for ZcNLV and DlRLV, respectively (Fig. 7).

### Replication of ZcNLV in *Z. cucurbitae* and *D. longicaudata*

To assess the infectivity of ZcNLV in *D. longicaudata* and their host, *Z. cucurbitae*, Tag-PCR was performed. The Tag primer and virus-specific primer were able to specifically detect the replicating strand of ZcNLV. The results showed that the positive sample successfully detected the replicating strand in *Z. cucurbitae* and *D. longicaudata*, while the negative sample and water (used as a negative control) did not show any amplification (Fig. S2).

## DISCUSSION

Meta-virome and high-throughput sequencing technologies are revolutionizing our understanding of the viral landscape, leading to the discovery and identification of an increasing number of viruses (16). Furthermore, metagenomic analyses have become indispensable tools in the identification and monitoring of insect viruses related to economic and public health. For example, more than 23 novel viruses were identified in honeybees and mites across China during 2016–2019, which provides support to the point that mites are an important reservoir and spill-over host for honeybee viruses (17). Sequencing of the meta-transcriptomes of 31 different tick species from China revealed the presence of 724 RNA viruses with diverse inter-genus compositions. This finding serves as an early warning for the growing global prevalence and expanding distribution of tick-borne viruses, which pose significant health concerns (12). In this study, we identified nine novel and three known RNA viruses from three species of hymenopteran parasitoids of tephritid flies. We characterized the organization of viral genomes and their phylogenetic locations. Furthermore, we analyzed virus-derived small RNAs and examined the distribution of those viruses within populations of *D. longicaudata*. These findings indicate that the parasitoid wasps *D. longicaudata*, *S. endius*, and *F. arisanus* harbor a diverse range of RNA viruses, providing substantial evidence of their genuine *in vivo* infections.

All nine novel RNA viruses have nearly complete genomes in comparison to their closest relatives (Fig. 1). Both DlNaLV and FaNaLV belong to the Narnaviriade family, and phylogenetic analysis revealed that the two new viruses formed a clade with their closest relatives. It is speculated that the same narnavirus may be capable of cross-infection between the two populations of parasitoids. It is worth noting that DlNaLV exclusively infects female individuals of *D. longicaudata*, potentially due to the virus-induced lethality in males. This phenomenon has been observed in *Drosophila*, where the male-killing partitivirus 1 encodes a protein, known as the partitivirus male-killing protein 1, that leads to the death of male *Drosophila* (18). In terms of genome structure and phylogeny, DlRLV exhibits typical characteristics of a rhabdovirus. Currently, two studies have been conducted on rhabdoviruses in *D. longicaudata* (19, 20). However, based on the genome structure of the virus, it is different from the virus described by J. Simmonds et al., but it is unclear if it corresponds to the virus described by O. Lawrence et al. Additionally, we have discovered a tombus-like virus (DlTLV) that infects the entire population of *D. longicaudata*. Despite tombusviruses traditionally being considered plant viruses, there have been numerous reports, particularly in arthropods (10), demonstrating their presence in animals. Therefore, further research is needed to investigate the impact of the virus on the parasitoid and its potential to enhance parasitic abilities.

Among the three known RNA viruses, ZcNLV belongs to the Negevirus group, a positive-sense single-stranded RNA virus. Negeviruses were initially isolated from mosquitoes and whiteflies in the Americas, Africa, Europe, and Asia, and currently lack a definitive viral family classification (21). Interestingly, the replicative form of ZcNLV was detected in both *Zeugodacus cucurbitae* and *D. longicaudata*, indicating the ability of this virus to infect both the parasitoid wasp and its pest host, the melon fly. This suggests the existence of certain interactions among the melon fly, the parasitoid wasp, and ZcNLV. Similar to the role of Rondani’s wasp virus 1 between *D. melanogaster* and *P. vindemmiae* (9), further research could investigate the biological impact of ZcNLV on the melon fly and the parasitoid wasp, assessing whether the virus can enhance the parasitic capability of the wasp and improve biological control capabilities. VvAALV, which infects *Vespa velutina*, is found in the brain, muscles, and intestines of the hornet. However, there are variations in the viral sequences among different tissues, indicating a high degree of sequence recombination that may influence various physiological responses in the host (22). This study also identified the infection of the parasitoid wasp *S. endius* by the VvAALV, indicating cross-species transmission between hornets and parasitoid wasps. Furthermore, sequence analysis using the NCBI database revealed the presence of the VvAALV virus sequences in other insects of the order Hymenoptera (data not shown), suggesting a potential widespread infection of Hymenoptera insects by this virus. ZcDV, a member of the family Dicistroviridae, exhibits a high infection frequency within the population of *Zeugodacus cucurbitae* (23), suggesting its potential importance in population expansion and individual growth. However, further investigation is required to determine whether it represents a genuine infection in *F. arisanus*.

VsRNAs could serve as valuable indicators to assess whether a virus infects hosts by inducing the RNAi antiviral response (24). Following the viral invasion, dsRNA originating from the virus activates the host’s RNAi pathway. The viral dsRNA is processed by Dicer2 into 19–25 nt siRNAs (15). Consequently, small RNA sequencing provides a readily adjustable method to investigate viral replication in hosts and the activation of RNAi immunity against viruses (25). In this study, we analyzed the VsRNAs data obtained from four viruses in *D. longicaudata*. The data revealed a prominent peak at 22 nt for DlTLV, DlNaLV, and DlRLV (Fig. 6), while ZcNLV showed a weak peak at 22 nt in the second round of small RNA sequencing following an initial absence of a clear peak (Fig. S1). These findings are consistent with previous reports in honeybees (26) and bumblebees (27), suggesting that these distinct viruses can infect parasitoids and trigger RNAi responses. This indicates a close interaction between these viruses and the parasitoid host.

In conclusion, we have increased the diversity of RNA viruses found in parasitoid wasps, and these viruses may play a crucial role in the biocontrol of tephritid flies. Specifically, we identified sex-specific viruses, viruses with a 100% infection rate, and viruses co-infected with both parasitoids and tephritid flies. These findings are important for the mass production of parasitoids and the enhancement of their parasitic abilities.

## MATERIALS AND METHODS

### Insect rearing

*D. longicaudata* was initially collected from the pupae of oriental fruit fly *Bactrocera dorsalis* (Hendel) in Guangxi Province, China, in 2021. These cultures were subsequently maintained in a constant temperature incubator at 25 ± 1°C and 65 ± 5% relative humidity, with a photoperiod of 14 h light: 10 h dark (L:D). After emergence, the adult wasps were provided with honey and sterile water. Mated adult females were used to parasitize larvae of a laboratory strain of *B. dorsalis*. The rearing of *B. dorsalis* and *Zeugodacus cucurbitae* followed the methods we previously reported (28).

### RNA and small-RNA library construction

To characterize the RNA virome and VsRNAs in *D. longicaudata*, total RNA was extracted from 10 female and 10 male adults using TRIzol reagent (Invitrogen Life Technologies, Carlsbad, CA, USA). The quality of the extracted RNA was assessed using Agilent 2100 (Agilent Technologies, Santa Clara, CA, USA) and Nanodrop (NanoDrop Technologies, Wilmington, DE, USA) to ensure that the sample passed the quality control for the next-generation sequencing library. The Epicentre Ribo-ZeroTM Kit (Epicentre, Madison, WI, USA) was employed to remove host rRNA, and the Illumina TruSeq Total RNA Sample Prep Kit (San Diego, CA, USA) was used for preparing RNA-seq libraries. Subsequently, RNA libraries were sequenced using paired-end sequencing (150 bp) on the NovaSeq 6000 platform (Illumina, San Diego, CA, USA). Additionally, a small RNA library was constructed using a small RNA Sample Prep Kit (Illumina, San Diego, CA, USA) and sequenced on the NovaSeq 6000 platform (Illumina, San Diego, CA, USA) with PE50,using the same total RNA sample.

RNA-seq raw data for the other two parasitoid wasps, *S. endius* (SRR1038395) and *F. arisanus* (SRR1560649, SRR1560650, SRR1560651, and SRR1560653), were retrieved from the National Center for Biotechnology Information.

### Viral sequence assembly

In order to obtain potential viral sequences, we trimmed the RNA-seq raw data of three parasitoid wasps using Trim Galore. This step aimed to remove reads of low quality, contaminated with adaptors, and with a high content of unknown bases (N). Subsequently, *de novo* assembly was performed using CLC Genomics Workbench (29), Trinity (30), and Spades (31). Redundant sequences were then removed using CD-HIT (32). The remaining contigs were annotated with the Diamond software (33) using the non-redundant protein (NR) database. Virus-related contigs were manually identified. To verify the existence of novel viral species, viral sequences sharing >85% amino acid similarity with known viruses were considered to belong to the same species.

### VsRNAs analysis

Following quality control, the clean reads of small RNAs were aligned to the potential viral sequences using the Bowtie alignment software (34). The resulting SAM files were then converted to a sorted BAM format using SAMTools. Finally, the “viRome” R package was utilized to perform further analysis on the distributions of VsRNAs lengths and viral genomic positions.

### Viral bioinformatic analysis

Viral open-reading frames (ORFs) were predicted using the online software SoftBerry (http://linux1.softberry.com/berry.phtml?topic=virus0&group=programs&subgroup=gfindv). The conserved and functional domains of viral proteins were assigned using the SMART online tool (35).

Phylogenetic trees were constructed based on the conserved domain (RNA-dependent RNA polymerase, RdRp) of RNA viruses. First, the MAFFT software was used to align the RdRp amino acid sequences of each virus. Then, IQ-TREE (36) was employed to build the phylogenetic trees using the automatically selected best-fit model and the maximum likelihood method with a 1,000-fold bootstrap. Finally, the evolutionary trees were adjusted and enhanced using FigTree software (http://tree.bio.ed.ac.uk/software/figtree/).

### Virus and viral RNA replication assay

To investigate the prevalence of each virus in *D. longicaudata*, viral-specific primers targeting the RdRp region were designed using Primer3web (https://primer3.ut.ee/). Total RNA was extracted from 10 female and 10 male specimens following the previously described methods. Subsequently, cDNA was synthesized using TaKaRa’s PrimeScript 1st Strand cDNA Synthesis System (TaKaRa, Dalian, China) after DNaseI digestion (Invitrogen). To ensure sample quality, *β-Actin* was selected as the internal reference gene. A returned sample from transcriptome sequencing served as the positive control, while water was used as a negative control. Positive PCR products were validated through Sanger sequencing. For viral replication detection, the method of Nguyen et al. (37) was primarily followed. Unlike conventional reverse transcription reactions, Random six mers and Oligo dT Primer were not added. Instead, a specific primer, NS1, designed according to the negative strand RNA of the virus, was utilized. NS1 includes the Tag sequence (CGTATGCCGTCTTCTGCTTG) and the virus-specific sequence, allowing for specific binding to the negative-strand RNA produced during positive-strand RNA virus replication. Finally, the reverse transcription product was used as a template for PCR amplification with NS1-F and NS1-R primers (Table S1).

## SUPPLEMENTAL MATERIAL

Table S1, Fig. S1-2 spectrum.03139-23-s0001.docx Data.

ASM does not own the copyrights to Supplemental Material that may be linked to, or accessed through, an article. The authors have granted ASM a non-exclusive, world-wide license to publish the Supplemental Material files. Please contact the corresponding author directly for reuse.
