# Supplementary material for: RNA virus diversity in three parasitoid wasps of tephritid flies: insights from novel and known species
Source: Microbiol Spectr. 2023 Nov 6;11(6):e03139-23. doi: 10.1128/spectrum.03139-23 (PMC10714968; doi:10.1128/spectrum.03139-23)
Supplement: Table S1, Fig. S1-2 — Data. [file spectrum.03139-23-s0001.docx]

**Table S1** All primers used in this study.

| Primer name | Sequence (5’-3’) | F/R |
| --- | --- | --- |
| DlTLV-F | TTCCCGAGCATTTACGACCC | F |
| DlTLV-R | ACTGGTATCCACATCACCGC | R |
| DlNaLV-F | GATGAGGTCCTCGTAAGCCG | F |
| DlNaLV-R | ACGTGGCTGAGGGAGACTAT | R |
| ZcNLV-F | TCACCAATCCCCGCATCAAA | F |
| ZcNLV-R | TTGAGATGAATGCAGCGGGT | R |
| DlRLV-F | TGACCGGACTCGTCCATTTG | F |
| DlRLV-R | CCAGGAACGGGTTGCATTTG | R |
| Pv-Re-S | CCTTTCCGTTTTCTAGCCTC | F |
| Pv-Re-A | TTGCTAACAACACCCTTCAG | R |
| NS_ZcNLV | CGTATGCCGTCTTCTGCTTGTCACCAATCCCCGCATCAAA |  |
| NS1 | CGTATGCCGTCTTCTGCTTG | F |

**Fig. S1 Distribution of small RNAs derived from** **Zeugodacus cucurbitae negev-like virus in *D. longicaudata*.** Size distribution analysis and schematic representation distribution of RNA virus-derived vsRNAs, red bars represent the vsRNAs from the positive genome, while the green bar represents the vsRNAs from the negative strand.

**Fig. S2 Detection of ZcNLV replication in *D. longicaudata* and *Z. cucurbitae*.** M: maker 2000; + : positive control; - : negative control. The target electrophoresis band is indicated by the white arrow.

>Diachasmimorpha longicaudata tombus-like virus

CCCCCCCCCGCCCTACCCTGACATCTCTCCCAATGAGATTTCTAAGGAAGTTTTGCAAACATATATTGACGCGTACGATACCTACAAGCGACTGCAAATCAGTATGCGTGCCGACAAACAGATGGCAATCGACTTCATCAATTCTAAAGCTTTCGGGAAAACGTTCGATCGTAATTACATTAACTACATTACAGCCTTGTTTAACGAATTCTGCGACACACACAATCTCGATCACTCATTCAGGTCCGCCAATATTCATGAAGTCATACAAAAACACATCGAATGGAAATTCAAAACGCCCAATATTTGGAATGCTGAGACGATTAAGACAGCTAATGACTTCAATGAAATGATTACCGGACAGGATAATAATGATGTGTGGTATAAACCATGGACATGGGGAAATAAAGAACGATTGCGGACTCCGGAAAACGAGCAAACCCCCTTCAGCAGACGCTCATCATACAATTGGACATCACTCCTGGAGCAAGCGGCCTTAGTTACAGTCACGGGTATAGGAATTTACGGCCTTTACCGCCTACTTTCCCGAGCATTTACGACCCCACAAGATATTACTATTACCCGATCTCTACCACCACTACAGCAGCCGCCTACGCCAAAAGACATGCTGCCGTCATTTTGGCAGATTATGAAAATAAGATTATTAAAAACCTGTATCGACACATCAAGAGATTTAGATATTCAGGTCTCCGCAAATGGACTAGAGCTGAGTACGTGGAGTCGATGGAATCTACCAATAAGCGACTGGCTTATCGTGAGGCCGTTAACAAGATTGAAGAATATGGTCGTATCTCCACCGTAGTCACTCCTTTTACAAAAATTGAGAAGTTAAGCACCTCTAAGTATAAAGCCCCTCGTATGATACAAGCTAGACATATAACGTTTAATGTAGAATATGGTACGTATATTAAACCACTAGAACGACATGTTACTAAGTATTCTAAATATACATATAATTTTGGTAAAGGTAACTTGGATGAAGTAGCACAGAAAATTGAAAAACTTAAAAAAAAATATAAATATTACACAGAAATGGATCATAAAACATTTGATGCCCATGTTACGACAGAGCATCTTAAATTAACACACACCTTTTACCTAGCGTGTTATAGACATGATCCATATCTAAGAAAACTTACAAAAAAAACATTAACCAATAAATGTATTAGTCGCGCTGGTGACAGCTATGTTGTTAAAGGCACTAGAATGAGCGGTGATGTGGATACCAGTTTTGGCAATTCACTTATTAATTATGCTATCCTCAAAGAAATGCTCCATCAGCTCAACTTAAAAGGAGACGCCATAGTAAATGGCGATGACAGCATAGTGTTCACAGACAAACCAATAAATATACCAGCAGCAGTTAAAGTATTCCGCACAATGAACATGGAGACTGAAATAAAACCTAGTCAGACGAATATAACAGATGTTGAATTTTGTCGATCAAAATATGTCATAAATGATATGGGAAAACCAACAATGATAAATGACCCCGAACGCGCATTAACTACTTACGGTATGACTTATAAACAGATATCTAACTATCGTACTTATCTCACAGAGACGGCGTATTGTAATGCGTGTTTTTCAAAATCCACTATATTAGGGGACTTGTGGCTTAAATTTGCACAACAGATAGCCACGGGTCCCCTTCAAAACCTCACCATCCCAATGGTTGAAAAATTGGTAGTTACAGAACAATTTCGACATATCGACAAAGATACTATCTACAAAGTTAAAATGAATATATCGTCTGACTCTTCTGGCGGGACAACTGTTAGCGACTATTATCGAGCTTATCCATATACTTACGATTTTCCTCATAAGCTATCTATAATTAGAGATAAGGTGCTTAAACTTTTCGACGATGTATCAGAGGTGATGTCCTCACAATGGTTGACGAACAATGCTTACTATAAACGGTTTATCGCTATTTCACATACGAATCAGACATTGACACTGGCATAAACACAGGAATTCTTGCTGAAGGGGGACCTATCCAG

>Diachasmimorpha longicaudata narna-like virus

CATGGCTGCTGCACGACTCGGCTGGACGGGGAAGTGGAAACTTCCTCGCCTACCGGGGCCCGTTGCCGCGGCTGCGGCTCCCCCGGCTCTGGCGGCGGCCTATCGGCCGTCCCCAGGCGGGGTTACGGACATAATGTCCTATCTCGAGAGACTCGGCATCCCTGCCGGGTCCCTCGCTCCTCCCTCTGCCGACTCGGTTCGTCTGGGTCAGCAGATTCCCAAGGGGATTCATCCCCTCCGGTCGCAAGATCTTCATGACCGGTTCCTTCCTGCCTTGGCTGAACACCGCCGAGGCACCACCACCACCACACACTCCCCCACCAAGATCCGGGGGAAGGCAAAGGGTGCAAAACATCGCACCCATTCCCGAGACAGAGGCGGGGGCTGTCCCATCGGGGCGGGTTCGCCGGCTGAATGCCGCGATCTCCGCCGCGTCTGGGACAGCTTCCGCCTCTGTTTGCTGGCACTGGTCCCTCGCCTCCAAAAGGAGGGTCTCCGTCTCTCTTCCCGTCGGGAGAGGACAACAGCCGTCTCGATTGAGACTGCCCGTCGATGGATTAGCCGGTCGTGGGTGGCATCGGGTCGCAACGAGACCATTCGGCAGATCAAGATCTATGCCGAATACTGTCGAGCACGGGCCCTGCGTGCGGAGCAGGCGGCGGCTGGACCCCCCCGTGGGTTCCCGGTTCGCGCTTTTGAGCGCGGTTTCCGGGCACTCCTCGGGGATCCAGCTCTCGCCGCTACTGCCTTCGCGCAGTTGGCCCGGCTCGGCAGGTCGATGCCTCCGGCGGATAAACCGCTCCTGCGGGCGGCGCTTGAAAAGCACCGCGCCGTTCTGACTACCCCTCCGCCACCCGTGGCGCGGGAGCTGTTGGACTCCCTGTTCTTCTGGGCGGAGGGCTGGGCTAAGACCCGGCCCGCCGCTCTGAGGAACTCGGTGGTCGCCAGCTTCTCCTCCTCCGCTTCGGAGGGCATGGGAAGGGCGAAGGGGGGTCAGAGGGCCGAGCTGGAAGAGCTCGCTCGTCAGGAGATCGATGAAATCCGCGCCGACCTGGCCGCTCACCCGGATACTCCGGAGGGCTTCTTCGACGTGGCTGAGGGAGACTATGAGGGACTGTACTTTGTGGAGTGGGACCTGACCATGGTCGCCGACTCGGCGATGCAGCGAGTCCGCCAAGAGGCAAGCGATCCAGCATACATGATCCCGGTGCGGGCGGCAGCCCTCCCTGAACTTGGTAACAAGTCCAGGGTGGTAACTGCCCCGCCCGCACACTGGGGGATATGCGCGGATGCCATGAGAAAGACTCTCTGGCCTCTGTTGGAGACGGACTCGCGCGTCGACCTGAGTGGTCGCCGATCCCTCGCAGGTGCTGCAGCTCAGTTCCACGACCAGGTTGTTAAATCTCTCCGTGGAGCCGCAGGCCAGTGGATGTATTCGGCTGATTTGACAGCCGCAACGGACCTGATGCCTGAGCCGGTTATTACCGCGCTCTGGCTCGGTCTGTTGAGCGGTCTCGGCATCCCCGAGGATTCCTTCTTCGCTCGCGCGGGAGGGAAGATCCTCGGGAATGTCCGGGTCCGCTATCCTGACCTGTCGGACTCCGACATGGGAGAGGGTCCGTTCAACTCCGTAAGGGGTTGTATGATGGGCCTCAACCTTTCGTGGTTCGTTCTCAACCTCTACAACCTCGCGGTGGTGGACCTCGCCTGTATAGGCGGCCACCAAGCCGTCGTGGATGCGGAGGGAGAAGTCGATGTATTCGAGCTTGCGCGCTGCATTGGGATCGCGCCCGCGATAGTCCGCGGCGACGACTTGGCTGCGGCCCTGACAGAGCGTCAGGCCTCGGCTTACGAGGACCTCATCTCCCTTACGGGGGGAGAGGCGAATCGCGCCAAGTCGTATCGGTCTACCACTTCCTTTGTGCTGGCGGAGAAGTCGTTCGTCGTCGAGCGCGAGGTGCGTCCGTTGACCGGGGGGCAGAAGAGAGGCTTTATCAGCTTCCCGCCCTCGCTGGCCGTTGACGCTCCGGCTCTCGCTCGTGACCCGCGGGCCCTTGAGGCCTTGGGCTTCGAGACCCCCTTGGTGCGACTGGGACTCGGAGGGGCGGGATGGACTGACGCCGCTGTAAGCGTTGTCAGCACCCTGACCGTCCTCGACGATATCCCGGTCCGCCACCTGATGCCGGGGCCTTCCTCGGAAGGTCTCCCCGCATACGTCACGCTCCCAGCTGCTGCAGCCGATGTGCTGTACGCAGCTATGGAGGAAGGGAGTGGAGTCGCGAGTCGGATGTGCTCCGCGGTCTTGAGTGTAAACTCGGACCTCGTTGAGCGTTACCGTTCTTACGCAGTTCCTCTCTTTCTCCCCCGTGATGTTGGCGGTGTGGGCTTCCCACACCCCAAGGGGTACTCTCGCGCCCTCGCCTCGGGCGGTCGCGGTCACTGGCGTCGCGCCACGATGCAACTGACAACTTACGGTGTCGACCAACGGTCCAAACGAAAACTGGACCAAGACCCATGGCGGGCTGACAACCCTGCGGTCGATCGCTCGGCCGCGGTTCGTCGCCTCCGCCGACAGGAGCAGGACGCCGAAGCGCTGGGTCGTCTGGGCGAGATGGTCCCTTCTACACTGGACGAAGAGGTCACCCGCGAGGTGGCCTTCGAATCCTTGTGGAGGGACCTGTTTTGCCCGGCGGACCGCGCTGAACGGTGCGCTCCTCGTCGCAACCGTGGGTCGAGGCTCTCGCGGGTCTCGTCGGAGCTCAAGAGGCTGTCCTCCGTCGCTTTGCGTCTGCCATTTAACGACCGGTTCCTGGTTCGTTCCGTTAACCGGAGCGAATTCCTGGAGCGGGTCGGGGCAGCTCGCGGCGCGGAGACAATCTATGTGCCTCTGGCTGAACGAGATCCTCGCAGGTTGATGCCTCTCAGCGACGACCATCCGTCGCTCGTTGGTCTTCACCGACTCCGCCGTAAACAGCAATTCTCGGGGGCCGCTGTTCTGGCCTGTACTGCTCCCCTTGCCGAGGCAAGGGGTGGTGGTGCGGCCCGAGATGTGGGTGGGCCGGTGGTGGGTGATGTTATGGAAGGGGAAGAAGGGATTAGGGAGGATGAGATAGGATCTCATGTTCTCACACGGGGAACCCCGAAAGGGGCTGCCCGTCATGAGGGGCAGGGGGCGGTCGCTGTCCGAACCACTAGGTCCGGACTGGCGCTCTCCGACGTGGCTCAAATTCGTGAGCCGCGAAAGAAGCGGCGCCGCGGCCGTTCCCCGACCACTTCCTTTCTAGTCAAAGGAGGCAACGATATTTCTAGGAGTCTCGTGAGTCGAAAGGCTCAATACAATGTGACGTAA

>Zeugodacus cucurbitae negev-like virus isolated from *Diachasmimorpha longicaudata*

CATAACACTAACAATAAACAAAAGTATATTTGTGGAATATACTATAAACCTTTCCACAACACCATGTACACAAACGCAAACAACGCAAATGCTTTTCCTCCCGTTGCCGACCTTAGCAAAGTTTTTGGATCTATCGATGCTGATTACTCTAATAAAATCATTGAATCCCTTATATCATCTCGCAACGAAAATTCACTTAAAAATGTCTTCTCTCAACATTACACCGATCTTTATCATAGTCACGTCAACAACAAGGCATACACTCGTAGTGTCTGCCTTACTAATTCACTCAACACTGATCAACTCACCACATTATCCAATGCATACCCTGGTTTCAAATTATCATGTTCCAACATCCAGATCAATTCCCATGCAATGGCCGCATCATCTCGCCATTTGGAATATATCGAATTGCTTTATGAACGCATTCGTTATGATGTAGATAATCAAGACAAGTACTCCAACTTGCTTGCTACTACTCCTTGGGATACCTGGGTTAAGGATGTCGGCGGTGACCCAACGAAAATTTACACCAATGACACCAAGTACATGCATAGTTGTTCACCAGTACTCATTAATAATGCATATGATTCAGTCCGCCATACTTCAAGACTCATTAACGCATACACCCATCCGTCTATGTCTGATCAACACATTAACGTCATGAAATCATTAATTCATAATGAGTCAAACATCACCACATGTCGCAATTTAGCTCAAAAATGTACCGTTAAAGCCCCATTTCTCATGTTTGTTCAATCCATTTGGGATATGTCTCTGACTGACGTAGCTGATGCTATGGATAGCGCTTCAGCCTTGATTGGTTATGGTACCTTCATCTACTCTGATGAGATCCTTATTCACCAAAGTGGTTCACTTAAGGAGCTCGGAGTGTGGTGGGAATACACTAAACCAATTAAGAATCGCCAATGGAGTCCAGAAGAAATTCTTCATTACTCTGATGGAGATATCATCATGGGATTTGAAGGAGATGATGGATACAACTATGTTCATAACCTCGCCAACTACAAATCTTTCTTAACGACATCCAGATTTTGTTCATCTAAAAAGCAATTCTATAACTTGGAGCTGTTGGAGAACCGTATTGGTGTTCAATATTTCAAGATCACTAGAGAATGCATTAAGGGCGTGCGTGCATCGGTCCCGCACACTGTTCATTTAAAAAGCTTGAAAGACAAATATATTTTGTCTTTCTATAAGGACAGTGCCCCTGGTAGACCGTGTGTCGCCCCTTCTCCAGCGTACTTGGGAGATGACATCACTTGGATTAAAGATTTGCAGCCTAATCTCACCCCCATCCGATTTCCTGTCGATAGCAAGTTGGTTGATTCAGCTCAAGATTGGGTTTCCGCTGTTAAGGATAATTTGAAACCGCACGAAATTCTGAATTGGTTACGTAATTCTTCGCGCAAGGCAGTACATAATGGTGCTATAGTGCAGAAAAATTCTGAGCTGAATTCTGAACAGCAGAATGCTCTAGCTCGAGTGCTCTATATCATCACTTATGAGAAGAATTATCAACTCGGGAAGATTATACAACAACTTCAATCGGATTTGGATACGAACAGGTCTTACATGTATTCACCATTCAATCAATTTTTCGCCCCACGTAATTACATGAACATTAACCAACCCCGTTCTACATCAATAGCAAAAACAGTCTATCGCACCTTTGTAAACAATAGACGGTGCATACTCACCACCATTCTTGCACCAATTCAAAGTTCCATCGTCGCGTACTGGTCTCCTCGTCTAGCATTGTATTCATTACTCCCAACCGCTCTGATCACTTACTTTTGTTTGCCCCGCAATAATCTATTACGCATGATCGACGTGGACAAATTCCTCATCACCAATCCCCGCATCAAATACACGTATAAGTCCGTCAGCTATACCAACCATAAAGGAACAGTTTTTAATCTCGGTATCCCATCAAAATATACCGATACCGAAGCCGACAATTTTGTCCGAAAAATTGCAAAAGAAGTTTACAATTGCGATTTGGAAGGTCCACTGAAGGATCACATCATTAACCTCACCTGCAAGGACAAGAATGCCGAAAACTCTCCTTTGTTGATGGAGATTCCAAAAGATATAGGTGAATTTCAGCCTTTGACCATAGATATTCCAACATCCTCGCCTGGTCATGACACAGTTGATCCCAGTCCATCGTCCGCATCTTGTGATACTCTTGTCACCTGTCTCTCTCCCATTTCGTCTGTCTGTTCTGAAGCCCCAACGCAAATCACCGAACTGTCCGTCGAAGCCCCACGATCTCCATCTGTCTGTTCACCAATGTCCCCAACTCAAATGAGCGTAGTAGATGAATCTGAATCTTTTACTGTTGTCGACATTCCTCCTTATGGTAATTGTTGTTTCATCGCAGCCAATGAAAGCAGAGTCTTCGATATCGAAAAATTTAAGAAAGACCTATTGGCATTAAAGTTGAATGACGATCGCGATTATATAGAGGAATTGTCTGGAGCATGGGGAGGACGCGCCTTCCTCGATACATATGGTAAACACATGGGTATCCGATACTTCATACATTGCCCTATGGTCGAAGAAGTCGGACACGACAATCCTACCCGCTGCATTCATCTCAAGTACAACGGCTCTCACTACGATTTGTACGTCCCAAAACACTCAGGCACACCATCTTCTACCATACATTATTTGCATTCCCCTATCCCACCATCAATATTCGATCGTCTCAATCGCTACATTCAATATCTGGACATAACCGATCACTCCCCTTCTGGTTACTATTACTCAGGTAATCTTCAACATTATTGTCATTTTCACATAGAATGCGAGAACAACTCATTCCAACGCACTCTCGATGTCGTTATTAACCATCCTCATTTCCAGAAGTCCTCATTCATATTTCTATTCGAGATTAAGAACTTTTCGACGGTTCTGTTCAACAAGGTTTGTCAGCAGTTCAATCTCATTCACACACTTCACTTCATTCCTATTCATGGATACATAGTCATATCAATCATGTCCGGGTCTCCAAAGAAACTCAGCGATATGGACATAAAAACCGTTACCGATCACTTAGCTAAATGCCCGTGCGGTAAAGACAACTTCGTTCCAAGACCTCCCGTGTTTAATAACAATGCTCTACGGTATTGTCAGCAGCACGTAGACGAATGGAGCCAGATATGTCCATCTGAAACAGACCTTACCATTAAAATTTTTGACATCAAGGACACCATTGACGCTCATCAATCATTTGGTCATCGCCACCCTGACAATAAGACATTTTTGTTTTATTTCCAGAGTGATGGTCATTCAGTCAAATATCAAGATGATACTATTGATCTTCTTGAAAAACATCTATCATTAACCAACAGGGCCAACACCACCGCAATTCATGTCATTTCGAGGTTGTCTAATGTCAGTCGCATACTTAAAGCCATCTCTAGGCAGTACGAATTTGTCGCAACAAAAACTATAGAGTATTTGGCTAGTTCGCGCGACACCACCATAGGGAGGACCAATGATCTTACACCATTGGATGTTATTGTCTCTACCATGGAAGAGAGGCGTGAAATTTGGCGTAGACACCCACAGTATGTGACTGAATCACTTAGGAAGTTTCATCAAACAGCGATTGATCGGTACGAGAAAGGATTACCTGCCACTGCTATTGATCTTCGATTTCATCTTTATGACGTCAAGAAAGGAAAAATAGTTTGTGGTACAGACGTTGACACTAGGACACTCCATTGGGTCTATGATGGTACCCGATTGGTGGAGATAGATAAACTTGCTCGAGAATCAATCATGAGCAAGACTAGTTCTCATTACGTCTCATTCAATCAACACTCATTACTCATCCCTGCAGTTTACATTTACCAACTCGTCAAAGACATCGATATCACCACCATGCAGTTTAATACAGCCATAACCGAAATCAAAGGCGTCCCTGGTGCGGGAAAAACAGAATACATTCTCAACAATTGTGATAAGAATGACACTGTTCTTCTTCTAACAGTCAGTAGAGAGGCGAAGGACGACATGATAGCTAGGCTGCATAAACGAAACGTTGATCATAACATTCAGGTCGCTACGGTAGACTCATTTTTCATTCATTATCACAAACATCACAGTTCCAAGGATTACAAGCAAGTATGGTTTGACGAAGCGTTACTGACTCATGCTGGTGATTGGTTGTGGGTGGCGTATTTAACACGTACTCCGAAACTTATCATCGCTGGTGATCGCGCTCAAATCCCGTACATCGAAAGAACAGGGTATACCCCTCGCTTTGCTTGTCCTCAGCTTCCCATTAAGAACTGCATTAGACTCTATACATCCCACCGGTGCCCAATAGACGTCGTTAACTGGTTGAACACTAGTAATAAAGGTGTTCCTTTTTATGATTCTCCAGTTACAACCAAATCATCCATTTCGAATTCATTGTCTATTGTCCATATATCATCCATTGCTGACGTCCCATGTGATATGGAGGCCCATTATTTGGTATTTACCCAGTCCGAACTCAATCATGTCAAAAATTCAGGGTTCAAAGGTAAGATTTGCACCGTTCATCAATTCCAAGGCAACCAGAATTCCAAGATTTGTCTTATCAGAACTGAGATAAAGGATGCCTACGAAGTTCATCGATCAGTACCCCATATACTCGTAGCTCTCACACGTCATACACACCAACTCATTTATTACACTGTCATCCGTGGTAAACATGCAGTAGCCGACATCATAACAACCATTAATTCAAAACGTGGTGGTGGTGCCTACTTTGACACTTATCGTGAAGTAGTCACTGAAAAATCATCAACTCGAGTTGTTCATTCATACACTCCTACTATTAGGAATCTGAACGACAAAATGGGATTTGGTGCTTACATACCCATGCGTCCACGTATTTCATACATCATCGATCAAAAACCACGACAGCCCAGTGTTTATCCTGATTTGGTCTCCAACGCACGTCATGTGTTGCAAGACTTTCATGATAAATACTTCCATTCATCATTGACCACCGACCCAACATTTGATCATGATCTCTATCAACAATCCGACAAATCATTCTTTGGCGACTACACTATAACTGAGAATTACAAATATTCTACAAAGTTGAACAATTGTTCACCTACGTTGAATACTTGTATTCGTCAGAAAGTACCGAACACGCAAGCTCAGGTTATCAAGGCATTTTGTGAAAGAAATGGTGCTGTCCCTGAACTAAAAGGTTTCGTTGACGATGAAAGCATGGCTGATCGTCTTGTGGATAGTCTGCTTAAAGTTTGCAATCGTGAGCTTTTAGCGGAATATGCAGAAGATCCTCTGCCGGCAAATTGTTTTTCGTTGTCTCAATGGCTTGCCAAACAACCTTTAGCAGTTCAGAAACTCATTGACTCTGACCCCGTTGATCTATGTGATAAAGACCTGTCAGTTTATTCATTTACCCTCAAAGGTAATGCTAAACCAGACTTGGATCCGGGCCCAGACGAAAGATATAAGAGTTCACAAACCATCGCATTTCCCGAGAAACTTGTTAACGCCATTTTTTGTCCCATATTCTCAGATATCACCACTCGCCTTAATGCCATCCTTCCACCAAACATTGTTCTGTTTAATCGAATGTCCATTGAAGAATACTGTTCCGCTGTCGATACCGTATGCCCTTTTGAGAGATTTCAAAAACTCGACAATTTCATCGAAGTTGATTTCTCTAAGTACGATAAGAGTCAAGACTTGACCACTTTGTATTTTGAGATAAAAATGATGAAATTGTTTGGTGTTCCCGACGAACTCATCAGCATGTGGGTGGTGCTTCATCGTACTACTCATCTTGTTGATCATCGTAACCTGTTTAGAGCCACTGTGCACTACCAACGTAAATCAGGTGACGGAGCAACATACATTGGGAATACTATGTTTAATATGGCGGTTCTTCTTTACACATTGGACATACATTCACTCATCAACAATCAATTGGCTTTTTGTACTTTTAGCGGGGACGACTCTCTCATTTTTACACCTAATGTGTCCATAAATCTCGCCGAAGCATCATATTTGTGCAGCAATCTATTTAACTTGGAAGTTAAATTGTTGAAGTACAGAACACCATATTTTTGTTCGAAATTTTTCATTCCAACTCCTGACGGTCTGTTACTGGTTCCCGACGTCATAAAGACTATTGTCAAACTTGGTCGTTGCGATCTCGTCAATAGGGATCATGTGAAAGAATATTTTATCAGTTTTTCTGATAATAATAAACTTTTTAATAATCCGTATTTGTGGGACTATATCGCGCATTCCATGAATGACCGTTATGGATTTGTAGGTAACCATTATCTCACACTGTCAGCCATTTCCACCCTTACTCGCGATTTTAATCAGTTCAATTCCTTATGGGATTTTTCCATTGATCATCAATACTCCATTCTTCCATCTTTGGAGATTTAACGCGTTCATTATGTTATTGGATTCACATTATCATGCTTCTCATTATAATTCTCGCTATTCTTCATTACTCGTCTGCTGTCGTTTATACTGACGGCGAAACACTGCAAAGCATGTGCTGCAAGTGCCGTGATAACAATCTTAACTGTGCTTATTGCAAGTTAACCATGTTACAAGGCACTCATGCATGCGACATTCGCGACGACATCTTATTCGCCAATTGGGTACTTTATGTCAATACCTTCATCGCCAATCATAAACTCGATGTTCCATATAGGTCGCAATTATCATCTTGTGGTGGTTACATGTATTTCTATGCAGGTAACAACTACCCGTCATTTATCACATCAACATACAACTGTCAGAGGGTCACCAAATCTGCTTCATGTGTTTTTGGTAGTGGTGTTTATAATGTTTTTGGATGTTACTACACTGCTGTTTCTCCAGACTTAGGTGTCATGCCAGCGAACTGCGGCACCAAAATCTGTGAGGAGACGAACCCTTATTTTTGTTATGCCAATGGTTACTATCGCAATGCAGTCTGGTATACCAATGTCAATTACGAAAATAAAGTTGTGTCCGATCCATCATATAAGTGTTTTGAAATGGATGCCAGCGCTATGCTCACTGCCTTGAAATCCATGGGTTCACCGTGCACCGCGTTGTCCAATCCTACCTTCACTAACACTTCTGACACTGCATTTATCACCACATACGAATCAGATACATATGCCAACTTGCTCAATTATCTTAATTCAGGTAAAATCACTGCGAAGAGACATGTTAACTCTGCCATTTACACTCATTGCTCAAATAGCACCTCCATCCTCACTATGCTCGACGGACTTCCATATACGTACTGGTCAAACGTGCTCATTATTCCACGCGGTGCTTGCAGCGCTTACATACCTTTCAACTCCACCCACGACATCTGCGGTGATGACATTTTTGGAAATTTTTATACTTCCGGATGTCCTGACACCTTTCGAACAGTAGTCAAATATGGTACCAAAGTCGTCTTGTCATACCCTGTCCTCACTCCACCTCCTCCTGATGTACCGAACTACAATGTCTCCTGCGATACACTCAATAGTCATTTTGCATGTGCCTATAAAGATTGGATTGATTACTTCATCAAATCTTTTGGGACCGTGGATTTTGACTTTGACCTCGATAGGTATGCTCAATTGCAACATGAATATGCTTCTGAACGTCAAGCACAAACTCGTGACTGGCTCACTTGGTGGTACTCTGATGTTATCCAGTTCCTCTGGGACACTTGGCAGAAGATATTTTTGGAATGCATTGAACCATTCCTCGAGGCTGTTATTGAGGCCACATCTCAAGTAGTTCTAGCCATACTTAACACATACCTCGACCTCATTAAGAAATCACAACACTTCATTGACGTTGTCACTGACTTCATTACGAAAATATTGGACGTTTTGTTGCAACTTTTGGCCCTTATTTTGAAAATATTTCTCGGGCTTCTTTTAAAGTTGGAACAACACTTCCTTATTTTTGAATATCTCATTCTTTTTGTTGTTGTTAATTATTACTTGATTAATAACAATATCGTCTCATTAGTTGTGGTCTTTTTGGTGATGGTTATCTTCGGTATTGATCGACATTCTCCTTCTGTGCTGTTGTATTTTTACAATTCTCAGTACAATTACGTGAATCTTACTCAGTATCAGGATACCCGATTCAATTGGTCATATTCACTCACTTATCATCCTCACCCCGGTAATTCATCAGTGTCTGTCACTCTACCTAAACCTGGGTTGAACAACAGTTACGAGCTCATTCCTCTTCCTAATCGTACTCTCACATTACCGCCTCCGTATCACATTTCGCCTTCACCCATCGCGTGCTCTGGCAGTTTATATTAATTGGTAATCATTATCAAAATGCCCGCTGCTACCACAAAAACCACTGATGCCGCTGCAGTTCCTGCTGCCGCCAATTCTGTCATTCAGAACCAAAATAACTCTCCTTACAAACCTTCTACACCTAAACCTCGCGTATCTAATGCGGCAGGTGTTCAGACCATTAAGGGAGGTACTTACATCGGTCAAGGATTCTTTGATCAATGTCAGAATGCCTACACTAATCTGGTAAATAATCAACTCGCGTTGATAATGTTTGTAATTGGTTGTTTAGGTTGTATATCACTATACCATTCCACTCTCACCCCCATTGATCTTACTCATAATGCATTGTTGTCATCTGCCAACGACACCACCAATTCATTCGCCCTTCGATCAATTTCAGCCTTTCTCGCATACTTATTCAATCTGCTTCTCACCTATCAAACCATGATCTTCCCTATGTTGGTGTTTGGCGGTTGCTACGTTGCGAAACCATCTACTAACAATGCATACTCTGCTTCACTTCTCATTCTGACATCATGGTTGGGTTCATTCACAGGTATACAGGTCTTTGCCATTGGTCAACTATACCTTTTGTTCACCCAACTTCGCGACCCAACATACAGGTACTTGATCGCTCTTGCCGCTCTGGTCTCTGGTATCCTAGGCTTCAAACACACCGCTCAACTGGTTAACTTGTCCGGACTTACTGCTGTTACAGCTGGTGGTTCTGGAGGTTCCGGTGGTGGTGGATCTGGAGGTGGATCAGGAGGTGGATCCGGAGGTGGTGCTACTCAGACCCCTGAATTATATTATTAATCATTGATTAATCAATATGGATCAGAACAAGGTCAGAAAGAGTCTGCCACAAGCTGCATTCGACTCCTACTCGGTGTTGGTCGACAACCCATACGCATTGTTGACATTCGTCGTCGCTCTGTTTTGCATAATAGCAGAGGCCAATAATTCCTATGGTCCTTTGGAACTCATACACAACGCTCTCGTTGAGTATTGTCAAGGAGACGGTAAGTTAAAAGCCTTCGCTTCCATCATGCTTTACATAGTAGATCTCATCATTCCCATTAAGCTGCAATTCTTTATTTCACTCATGTTTCTCGTTCCTGCATTCATTAAGAACGACACATCCACATGGTTGTGTAGTTTAGCATTTGTAGCACTAACCATATTTACATCCATACCCAGTTGGCAACTATTTTTGTTCTCTCAAGCTTATTATATGTATTGTTTTGTGGAAAGTTCATTTTACAAATTTTTAATCTTCTTTGTAGCATTTATAGTTTTGGTTTTGGGTTTTCAGCATTTTACTTCTATTGTTGGTATCAGTTAATCTGTTATATTATTTAATCCGCATTAATCATATTATACTTATTCTAACTCAATCATGGAAGTATCACCGCAAACACTTATTGCCTTCGCAATTCAATCAGTCCGATCGCAGTTGTCCATCGCCAAGAACGTCCGACTCGTTCAAAACAGGCCTTATGTCTTGTTTGACAAGTCGTTCCAGAGGATAAATACCATCCGCATCAAGACCTATCGTGTCCAAGTTGATGCTCATGGTAGATTTGGTGTGCTAAGTGAGTCAGCTCAGAGATGTTTTGAATATCAAGATTTTCTCGAGGTCTTCAATGCCGCCAATGATATATTCGAACCCTCTGAATTGTCTTTTGATGGTAACTCCGTCGTGTTGCGCGCTATTCCGAAGGAAACCTTCCAGCCTTAGTAACTTCCCTCGCGGTTTATAATTTTTTCGCGT

>Diachasmimorpha longicaudata rhabdo-like virus

AAAAAGATGAATAGGACCCCCCTCCAAGAAGAGAAAACCAGGATGATAGAGGATGGAGAGCTGGATCTTTCTAAGAGGAAAACACATATCTCCTCCGATTAAAAAACCAAAGTAAGGAGAAACATATCCTTAAGACTCAGATTGTAAAATCGGTATTAAGATATTTTTCTCAAATTTTTCTGCGTGATTGTCCATTTGGCCATTCGTTTTCCTTTTCCATCATGGCAAATTTAGCTGATATTCGAGCTCTTCAACTTGAGCTTCAAAGAGATTTGGATCTTCAAGCTGTGGACGATGCAACCTTGCTCCCTGAGATTCCTATGTCCTCTTCTGCCACATTGACAGGGTGGAACGACCGGACATGTGCTGCTTTTTGTGAAGGCAAGACCCTAAATTCTGCTCCTAGGGCCTCAATCCCAAATCGAGTTTTGACTCTTAAGAGATTCTTCTCCTTTTATTTCACTGGCAATGTCCATCGCGAGGATAATCTTGTGGCAATATTAGGAGACACTATCTCTCTTCTCACTGTGTCCGGTAATCAATACCTTTTTCCTGATGACTTAGTTAGTGAGGTTCCATTTTACCCACTGAATAGGTCAACACCAGAAAATCTAACCATTGCTGGAACTGGAAAACAGTGTGAAGTAGTCTATCCTCCTGAGGTTGTTACACCGGAGGATCAAGCAGCATATAAACAAAAGTTAAAAGAATATACTCCTGATAATCATAGAGATTTTCTGAAGTTGGTTCAAGAGGCATTGGATCCCCGACATGTCGATCGTTTACCTCAACTCAGCCGTTTAGCAGGATATTTGGCACTGGTGTTTTGGAGAGGTGCCACAAAGGACAAAGGACAATTGGGAGATGGATTTCTCAAGGCGAATTTTGCTGAAAATTTGGTCCATCTAGTGGAGTGGAAAGCCGAGGATGCATACTCTCCCCCTTGTGAGGAGGCCATAGAGACCGCGTGCCGAGCGCTAGTAAAAGGACTTAAAACAACCTCTTACTTCTTTGCTCTTGTGGTAACCTATTATGCTTCATTGCTTCGGGATCCAGTCCCAGATGCGCAAAGAATTGGCTTGTTGGCAGCCTCCTTTCTCACTCATACAGCCAGGAACGGGTTGCATTTGATTGCAGCAATCATGAATGTGGGAAGTATTCTGAAGATCTCATGGCAGGAGTTGAGAAACAACACATACACATCTCTAACTAGAGCATCATGGAACCGTGTTCATGAATTTTTCAAGAGTCAAATGCAGATCCGCAACATTCAGTACACATTTCACTGGTCTAGAGTCATCAATGATGGATATCACAAAGATTTCACCGCAAAAAACAATCTTCAATTGTGCGCAGTTCTTGGAGGCATAATAGAGAAAGAAGAGGGGCCTGGTTACTTCAAGGCAGAGTGGGCTCGTCACAAGCTTGAGGCGTTGCAAGCATCCAGACAACTGGGTCATGCTGTGTACGCTGCCTATCAGCCCGACATCACTTCTCATGTAGCTGTTGGCCCTACCGCACGCCACGTTGCTGCTGCTCGAGTCTTGATTGAAGAAGCAGATGAAGAAGAAGGCAATCTGGGTGCTCTGGAGCAGGGATTTTAAACGGTTCTTTATGACCGGCTTAATCATGTTTAGAAAAAACCATAGTAAGGACATATATGTCTTTACTCCTACTGTACTTTTTGTTCTTGTTGAGTCTCAAATTCCAGTGCCGATTTTTCTTCTACATCTGGGCCTTTATAGCCGTCATCATTTTTTCATTCAATTTGTCTGACGTTGGTTTCTCTATGATCATGGCAGCCAAACAAGCATTGAAAAATAACTTTGGTGTCCTTTCCGTTCCGGAAGACCAAATGGACGAGTCCGGTCAGCTAGTTAGCCAAATGTCTGACGCTTATCCGGCTCACCTCTTTGGATCTAATCCTGATGGGGAAGGAGACCAAGATGAAATGGTGTATGAGGATAAAGTGGAAAGAGAGTCAGAAGGGGTTGCAACATCACCTCTATCTGCAGGAACGGAGTGGGATGAAGATGAATATGTCGATCCACCTAAGGAATCCTGGCGCGAGCAGGAGAAGGAGACTTATTATGAGTACGTGCAAAGCAATCTATTCAAAATAGAATATGCTCCAGTGGCTTTGAACAAATGTCTTGAATATGCGGCTGCCAGCAAAATCTTGATGATAAGAACTTGCTGGGCTACATATGCAGAATTTTTGCTAGAACTAGTGGAGAAAGATGAGATTGCTGTTCTAAGAAGTATTCTTAATGAGGCTCCTGAAGGAATTGAAGTTTTATCCGCAGTTTTCAGAGGGGTTGCGTTAGGGAAACAGCTCAAACGGAATGACAAGACAGCACAGATCGTAAGAGAAGTAGCCCATCTGCATGAGTCAATTCTAATCGCCGCAGACAGGATGGTTGTGAATCAGAAGGCACATAATGCCCAGATTTCTGAAGTCCTGAAGCAGGCGGAGAATGCATCAGTAAAACTAGCGGATTTCAAGTCCCAGGTTGAAAAAGTTGAACTGGTGGCTCTGCAGGCTGATTTAATGAGGAGATCACTTCCTCGTGAGCCAATTTCCATTCCAACGGTGAAACGGGGAAAAGATTTAAATCAGTCTAGCAGATACAGAAATGACTCAAGAGACTCAACCTCATCAGAGAGTCAAATAGAGGCTATTCCTCCAAAGATTGCACAGCTGAAACCGAGGACCGCAATTTTATCGGAAGGATATTATGAACATTATACCCTTCCATTAAAGTTGAAATTCGGTGGAGGGCTGCTAGTGTCAGTTGATTCGTTTGATCCTCCTCTGGCGCAACTTTCAGTCCTGATCGGAAAGCCCTTTAATAAGATGAAAGTGTTCTTTGAGAGAGATGTGCAAGCCATGCGGTCCAGACTAACGGCGGATCCAGATTGGGTGAACAAACTGCTGGCAGACGTCTCATTTTTAAAGGCTGAGCTTCACAAAATTCCCGTAGACAAGCTTCAATGGCGTCTGGTTTAAATCCATAAATCCTTTTAAGGACATCACATCAGGTTAATAAAAAACCATGACAAGGACATACATACTTGTACCTAAAAAGAGTTAGATCAGCATCTAAGAACCGTTTCGGCATTATGAAACCCACCACCTATATTATGGATAAATTTAAGGAAGTGATGAAACGCCCTTCTAGGCAAGATCCGCCATCTTCTCCCACTGCCCCGCCTGCCCCGGTAGTCAAAGGAGTTCTGGGGATCACAGGTCACATTGAAGTAGACTTAGAAGGCCCTTTTCAGTATAGAAGTCTCCCTTCTATTGTGAAGAAATTGTCAGTGAGAATTGTGAAACAGTTGTATATACAGTATCCACAACTTACGGAGGCTCTTCCTTCAATTTATTCATATATTAAGTTGTGCGGAATTAGAAGTTCTCCTTATTATGAGAACAATGGAAACGAATATGGCAATAGACTTGCTTTTTATGTCTCTCTTCGAGGATATACAAGTTCTGTATGTGTCACTATTGAAATGGAACCCATTTCCCATGTAGATGAGTGGTCTCTCCATCTTACAGGAGACAGGCCCTATCCAGTTATAATAACAGTCAGAGTCAATCTACTTCTTAGAGTCAAGACTGTAACATCAAGGGATGATTTTAGACTCGAAGGGTGTGTGCCGATTGCTATACCAAGACCTCATACTATTTCTAACCTACAAAGGAGACTTCTACGATCGGAAGAGGAAGGAATGCTAATGGATGGACCAGATGCTGGACCATCCTCATCACGAGTCTAATCCACTCTGGAATTAAAAATACAGGCTTCATATTTTATGGAGAACATCCGCATGCAACTGGACATAATCAATTTTGCGTTTAGAAAAAACCATAGTAAGGAAAGGTTATCCTTTTCAAGTGTCAATATGTATTCATCCATATTTTTCTCTGTTTTAACAGTTGCATCATCTATTGTCGTGAGATCTCCAGTACTTCCCTGGAACGTAACAATTACGAAACCTCCACAATGCGGATCCATTCCAGATATGTCAGAAATATCAGATTGCAAGCTCCAAATGAAAATGTGGACGGTGGACACTCCTCGCACACTTGATCACGGTATAATCTTTTGGAAAGAGAGGGTAGAAACATCCTGTACTATTTATTTTCCATATTCGAGAGAACTGAATGTAATATCCAGAACTCAATTACACTGTCCTGAGTGTGTTTCAATGAAGAAATCTCAGATTCTTCTGGAATCAACCTCCTCGGTAGACCTATATGATTGTATTTGGCCAGTCACTACGAAAAAGACAACCACAACCCTCCAGTATGTCAAGACAGAATATTGGGTGAGTTCACAAGGACAAATCACCACTCAATCAGGAATCTGGCATCCTACAAACAACACGAGAATATTTCAGCAGAATCTTCAGTTCTTAATCATATTTTCCTCTCCTTGGGAGTCATGTAATGTTGCACTTCTAAAAATGTTTGATGTGTGCAAAGTTCACGAAGATAAAGGGGCTCTTCAATTGTACTCTCCTGATGAGAATCTCTTTTTCACAATGATTAAAAAACAGTCTCCTATTTGTTCTAGAAAACAAGAACTCATCTATGTCTCGTCACTAGGATATCTCATCTCTTTTGACCAGAGCAATGATACATTGAATCCATTTTTCCATCCTACATCCATTAAGCTGCGGGATACAACTCAAGATGCTCAACTAAATGCCAATTTTTTCTTGCTAGATGACAGCGTTTTAAGACTTTCTAGAATGCTCATGAATCAACAATGTGAACTATCAAGATTGGAATGGTCCTCAATCAAGTCTTCTTTGTCTCCTCAATTGGCAGCATACTATTACACCAAAGACCCTTTCGGAACTGCAATATTAAAATCAAAACACCCTCCTATATTTGAAATTCACTCATTCGTACGTCAAACAATATCATCTTTTTCTTATCAGGGATGTACTAAAACTGGTTGTATTCAGTTACTGGTGAGAGGAGAGTCAATTTATCTCGAGCCATTCTCAGGTATTCATTGTCGCCCCTCGTTCAATACTGCAAACTGTCATCCGTGGTTACAGTTTAATGACACACATTCCATGAATGTTCAGAATTTCTCAACTGCCAAGACCCGAGAACTCCCATATCTTGACGTTCACTTGGCACATCCAATTGTTAACGGATCATTCCATGATTCTCCCATTGCGGGATCAGTATGGGAATTGAATAGAGCTACAGTACATTTTACAGGCCCGATAGATCATATCTTGAATTATTGGGCGTCTCTGTCTTCATGGGTTCAAGGGATAATCTACATTTGCCTCTTCCTGTTCCTGGCATGGGTGGTAATAAAAATAATTCCTAATTGCAGAAGGTGTAAGAGAGACTCTCTTTATCGCATAGTAGCTCACTAATTTTTTGCTTGTTTAGAAAAAACCATGGTAAGGGAAGATATGGCGTCAGACGATATCTTCTACGGTGACGACGATGGAAAGAAAGGAGAGGGAGATCCAGGTCCTCTCCCTACTCATTGTAATGTTCCACTAAAATATTCGAAACAGAGAGCCGCACTAGGAATCCCAGCCCCAGGTCAGTATGTAGGGAAAAGAGACTTTAATAAGATAGAATGGGAAGCCATCAAGAGACTGACAGGAGATCATGAAGTTAGGTGGATAGATTCAGCTCATATCCAAGGTTATCTTTTGGGTGAATTAGAATTAGAGGACAATCTGATCGTGAATCTTGAATTTGAACAGCAGCTTCGTATCGGAGGAGAGCAATTTTTTCTTCAGTCCCAGCCAGTGAGTCAGCTGGTCAACCCTCCACTTGATCCTTCTTTTGTCGAATTCTCAGAGGAAGAAAAAACATTCTTGAAGAAACATTATCTATTGCGGACATTATTTGAGGAAGGCGTTCTTACTTCGGGAAGTCGGAATTTTAAACCTACACTTTGGTGGAAAAAGACAGGAGATGTTTCCTATTTCAAAATGATGGGAGTAAATATGTATGTGACAAAGAATCTCTGGTTTGTTCAAACTCCATTAGGGAATGCCTTATTATCTAGGGATCACTTGCTAATTTTTTCAGATATGGTATCTCAGCGATATTTATTAAGACAGATCGCTATTCTAGAAGAGAAAAATGATAGAGTCAATTTTCTGTCCCTTCATGATCTTGAACTTCTATTAGCAAATGGGGACAGCATGCTTGAATTCGCAGGAAATGCAGCCTACAAGGTAGTCTATTCTTTGGAGCCTAGTTGCCTCAGCAGACTGATAGGGGACATCCCTGTCGGAACTGCTACGAGTCGAACCTTTCGAGAAATCCTCATGAGTGACATCACAGAACACGCAGAGAACATGAAAATTCTTCCATTACTGAGACAGAGAGAATCCATCCTTGATTCTCTAGGCAATGCCACTCAGATCACCCAGGCATTTGGCCTTTATCGTATTTGGGGGCATCCCACTATAGAACCACTAGCAGGAGTTTCAGCCCTTAAGAAGATTGCCACAAAAGTCCGAGGTTTCAAGCCAAAGTTTGTTGAGCAGATTGAGCTAAAATTCAAGGAGGAATTCATCATGCGATACATCGCAAAAGAGGGGAGATGGCCCACATTAGATGTCTCCAGACTATCCAATCTAAACCCTATCAAGACAGCTTATGAGTTCAATTCTTTATATCCAATTTCTCACAAATTCTATGATAGAGTTTCTCTGAAATTGGTAGATTTTGGCAAAACTTTTCTGATTGATCCTAAATTTGATATTGTGGAGATGCTTGCTGACAAGTCTTTATCATTATTTACTCCTGAATTGAAGAAAAAACTTGTATCGGGGAAAGGAACTGGAAAATCAAAAGAAAGATCTGTTCTGATCGAATGGATGAAGTCTAATCTCCATGATCCTGAAGAATTTCTAAGAAAAATTGACACCGAGGGATTTAAAGAAAACGAAGTATCTCTTGGAGTGAGAGAAAAAGAAAGAGAGGGGAAATTGATGGCTAGGTTGTTTGGGTTGATGACTTTGACAAAACGGACTTATATTGTATTGACAGAAGCACTTTTAGCTGAACATATCATGGAATATTTTCCAGAAATTACAATGACTGATGACGAGCTTTCTCTAGATAAAAAGAGATTAGATTTTACGGATGTATCATTAGCCTCCAGATCACTCTTCACAAGTCTGGATTTTTCAAAATGGAATTCAAATATGAGAGAGGAAGAAACAAAACCTATCTTTCAATGTTTTGATCATCTATTCGGACTGACAAATTGTTATCAAAGAACCCACGAGATGTTTAAAAATTCATTTATCTATCTTCTAAATGGATCATACACTCCCAAACTCGTTGGGGGTCAATTCGTGGCAGATATCGGATCATGGTACGGTCATCTCGGAGGAATTGAAGGCCTTAGACAGAAAGGGTGGACCATTTGGACAGTGATTCTTATCTTACTAGCTGCTGAAGATTTTCCTATCAAGCTTCGACTCATGGGTCAGGGGGACAATCAAGTTCTCAGAGAAATATTTCCAGAGGAAATTTCACAAGAGGAGCAAATGAATCTTCATTACAAATTCTTGGCTCGGATGAACGAAATTCTCTCTGAAGTAGGCCCACCATTAAAGATGGAGGAAACATGGACATCTCGAGACCTCTTCATTTACGGGAAATACTTGATCTACAAAGGAAGCCCTATCTCTATGTTTGGCAAACGCATGTGTCGGATGTTTCGATTGTCTAATGAAGATTATCCCACGCTGGAATCTACTATCTCCTCATTGACAGCAAATTATTCCGCAGCTCTCTCCTGCAGCTATGATCACAGTTATCTCTATTATCTCTATACTACTGAGGTCATAGGAGCCTTGCAACTCTTCTTCCGCACCTCCTATCTTCAGCCTGCTTCCCCGTCAAGGGTTTTGAGTTCTCCATTCACTGTAATCATTAAAGGGATGAAGCGGATATATGAAAGAAGAGTATCTAAATTGCTAGATAAGAGGAATATTCAATCAGATTGGATTTATCACGGGTTGATGTTGTTCCCTCGCTGTCTTGGCGGTTTCCCTATCACCTTGCTAGGGTCTTCACTAATTCGAGGTTTTCCTGATGAAGTTTCTTTAAATGTGAGCATCTTGAGAATGATTTATCCTCACTCAAACTCTAAGATCCAAGAGATGATCACCAACTTTATCTGTCCACCGAAGCATAATGACTTCAATTATGTTCTGATCTTAGAGCACCCCACTGCGTTGAATCTTGATGCTCCTTCTGCACCCAGCGAAGCTAGAAGGTCTCTCGTTGTTGATTATCTAAAACATTGTCCTTACGTAAAAAATCCGTCTTTATCTTCCTTTATATCAGTTCTGGACGATCCTCGAGCAAAGAAAATCTATGAATACCTCGCTAGTGCTACCCCTTTCGCACCAAGGGTATTGTCGTCTATTGTCGGATCTACGGTGGACGCCAGGGCAAGACAAGTAGCAGGAAAACTTCAAAAATCGGCAACTATCTCTGAACTTGCAAGAACTGAAGGTGAGAAAGATCTGTATACAACCATTGCAGAATCTGAATTAAACCATGCTGCCAACGTGCTCAGGCTTCTGGTCACCAAAACTCCTTCCCTAGTAACATGGAATCCCAACAAATGCTCTGTTCAGCATTCTGAAGATTTGAGAAAGTATAGTTGGGGGAAGACTGTGGTGGGTGTTGATTGCGTGCCTCCATTCGAGTTTATGCTAATTGAGAACAAACTTCCCTCTAGTCCTTGTCTGTCCTCAGTGGAAAGTGATAAAGGCAAAATCCTCATTCGGATATCCGGAGCTTCAACCCACGCAGACCTCCAAGATCCTATGGTGAGAGGGCAATATCGAGCTTACCGGGGATCTGTTACGAGACAAAAGGTTTCAGGTTATGGCGACAAGTTAGCCACAGTGTCGAATCCTGTAGTAAGAAATGCTCTGAAATTGTTCACCCTCATTGGCTGGAGTATAAAAGCCGGGGGAAATCTGCATTCCCTTGTCGAAATGATCAAGTCTTCAGTAACTGATTTGGACAGTGAACTTCTATACACGTGTCATTACCTAGGATCTGGAAGCCCACAACATCGTCACCAAGATATGAGAACCGCACACGGAGGAGCTGTTCCAATCCTTCCAAATTATGGTTCCAAGTTCGGGTTTGATACCTTTCCTCTAGTTGCTTACAGTAAAGGATCTTCAAATGTGAACCTTATGTTTCAGTCAATTATGAGTCTTACAACTGTACTAATGGGACCTCTGCTATTGCAATCTCAAGAATTACGACCAGAATCATTTCATATTCATGTCAAGAATAGTTGCTGTATCCGACCAGTGAATGAAGAGCAAATTGATTGCCCCTCTATTCCTCCATTATGTATTGTGAGTCACAAGGATTCAGAATATCTTTACGTGAAAAGCGACAAAATCCTGCCCCCGAACTTCAAGCAATCCGTGAGACTATCTAAACTCAAATCGACACAAGATCCAGAGGAGTCATCCTTGCGATTGTATAATCTCATTTGTGATAATATGATGGCAATCCTAAATCCAAGTGCCTGGAGTCGGTTCTCTGATCCCTCTCATGCTCAATTGACCATCAATTGGGTGTTGAAAGTGGAATTCAGGAAAGTTATGGAGTATCTTGCATTTTATTTCCTCGTATATTTTTCTTCAGAAGCAGATTCTAGTGATCGTAAAAGTTACCTGCTAAAAATCGCAGATGCAGTGGCTCGATCCCCAAAACGAGCTTGGGAGTCCTTAGAAGCATTGATCCACTCCCCCACTATTGCGAGGGATATTTCCGGTTCTCCCTATCATGCTCAAATTTCAGGAAATCCATGCATGTCCGAGTCTATTCTTGCAGGAAATCTGCGAAGTTATTGTTATGCTGTTTTGATTACATGGGCTAATGACGTGGAAGAGAGAGCATCAGTCAATCTTCTACAGATTAGGGCACAATTGGTTGGTAGTTTCACATGTCATCCCGCACTCTGTCTTGCATTTCAAAACTGGCTGTTAAATGATGATTTGTTTGCGTTCACGAATTTGAGGAAATCTCTCATCTACGCAATTAAGAGAGATGTTTCATCCCCTTGCTCTCTAACTCCCCTGATTCAATCATGGGTATCTGCAGCTGAGAGTATCACACATCCAGATCCAATAGACAATGTTGCAAAGACCAGTCCCTCAATCACGATCTCGTCAGTTATCCCGCTCACAGAAATCCATCTTCCATCTGCCACAACAGTGCTACTTAGTTTCAATAGATCAGATTTGACCAAAACTATAATGGATTTAGGCTCAGTAGACACAGTGTCCTCACGTGTTGTTTATTCCTCTAGAGAAAGGGAAATTGCATCACCAACTACCGGCAATTATAAGGGGCTATCATTGGTAGAAAGACTGCATCTGAAAGATCCGAAACTGGTGGCTTGTCTAGGAGACGGAAGTGGAGGATTTACATGGTCTGTGCTGCGCTCGCATCTTCAATGTAAGGTATTTTTCAACACTTTAATTGATATCTCAGTTCCAATTGAACAAGCCTCTGCTATCCCATACATTCCTTCTCTAACTGGTTGGGCGTCTATGCAAGATAGAGTACACCACATGGAAGTCACAACAGATGAGATCTCGGATATTACATCTCCGCTATTTCCCAAAGTTGTAAGTGATAAATGCGGCAAACTTGATGCAATTATATGTGACGCTGAATGTAGATCAACTTTTTCTTCAAATCTACCTCAACTTTTGGTGGACAGCGTAATCAAGCTTGCCTATGAGACAGACTGTCCCTTTGTAATCCTAAAGACCTATGCTGCAAGGCCTACTATGGTAAGATATCAGATATCAGTCATGCTCTCCTATTATCAGAATGTGTCGGTCTTAAGAAGCTGGTTCTCAGAATCAGGAAATACAGAATTGTATTTGCTCGCTTCAAATCCAGGCTGTCCAAGAGTTATCTCTATTATGGATAACAAGGCCCACGGGTTAATCTTGGCAGAAACTCACATCGCAACACTTCATCACCTTTCAGAATCCTTGCAGAAAGCTCACAATCGAACTCCAATTGAAATCTCGACCACATATACGAGAGTTATAGCTCCGGATGCAGAACTCCACACGCTCTCTCAAATCCAGCAAATTCTACCTTTCCTTGATGAAGGGAAAGAACTCACATTCCCTCATTCATTAGACAAATGGATAAAAATAAATGCCAAGGTGAGAGACATGCATATGATCAAAGAAGGAGCAGATAGTCGAATGGCTTCGTTTAAACATAATGACCTTCAACGATGGTTGTCTGCATGGTTGATTGTGTGGATTTTGGCAGATTTCCCATCAGAACAAGAAATTGAGTCAGTTTATACATCTGCGTATCTGGTATGGTTTCCTACAATGACAGGATCCTGGGAATTTTCTGTGAGCACATTGCTTCCTCCTACAGAAAGTCAAGGAAAAGAATTATTATGTTGGAAAATTGATCACTTACTTTCATCTATGCACATGAAAATTATCTCGAGACTTGTCTTCTGTGTATCCCAAAGAGGCTACAAAATTCGAACCCTATCAAATGGAGTTATGAGAGGAGGGCTAGACATTGCAGGAAGGAAAATACCTATTGCAGATCAAGCGAGGTCAATATGGGTAAGACAGAACATTCAGTGCATCACTCGCTCCAACCAGGAGCCAAGGCGTATTGGACAAACGGGATTAACTCTCCTTCCCCTTTGGGAACAAGTAAAAAAGAGACACCGCATTCATCACTGATCTCCATTTCGACAATTCTCATTTTATTTTTCTGGACTATCCTTATTCAGAGGATCCATAATTTGTTCGTTTATAAAAAACCATCCCTTTGTTCCCTACGTTCCTGGTCTTCATTTTTTTAAGGGAGGTAGTACGGCAAACGGGGCTCGTG

>Vespa velutina associated acypi-like virus isolated from *Spalangia endius*

GATGCTCCGGGGCGCTGAATATATCGTGCATAAGTACCTATATTCAGCGCCCCGGAGCATCAGTTCCGAAGAATCGATAACGATTGTCCAGAGCATTAGCTACAAAACGCCTTACAGAGTAAGAAGACACGATACGATAACGTTGAGTGTATGCGAATACAAAGAGACGAATACTTGTGGCACCTAAAGCTGATAACAGCTTTACGTGGCAAGATTCCGCATGAGGAGTTGAGGGAGATAATTATAAAATATCAAGTACATTTTAAAATAACCCCTTCAATATATAGTAGTCATAATAAGAATATAGTTAGACCTACATTAGAAGAATACTATAGGATAGCATATTAGGATAAGTTAGATTATAATTAGTGGAACTTTCCACCTAATCTACCTCCGCTATGGGAGTGTTAAGTAAGGATATGCCTGCACAGGCGTGGAATGGCATAACAACAGCCATAGAAACTGGAATGGATTATCTAGGGAAGATAATACGAGACATGATCATGAAGTTGGGGAGTGAGCAATTCATTAAACCACTTCTTGAACATGTAGGCACATTCGCCGAACTAATCTTTGATATAGTTCTGGCCAGCTATTATATTTTCACAAATGCAGAAGTATATGTTGCTGCCAGAATTGCATCAATGGGACAGATTACAGTGAAATTACTACGCTTGATCAACACTATTGATCGCATTTGCTGTAATAATAAAATTAAATTTTGCGTAAAATCTGCAAAAGAAGCTATGGAACACAGTTGTAAGCAACTGTTTTGTTCCTTAAATAATATAACACCCAACCAAGTGCGCAATAAGGAGGACAGTATGATGTTCTTTATAAACACAGCTATCAAGATGACATCCCTCATTCTTATGGGAGGATTGTTTGCAACAACTGGATTCAAGGCTATTGAGTTGCGTACAAATTTTGGTGCTACAAAAGAGGCATTACAGACTGGAGCAGAGTTCTCTAAGATCATAGAGGGCTTTGTAAAAGAATTCATTACCAACGAAATCGACCCTGATATTAGGATTGCAGAAGATCTTATTGCAAAGAATAATGAGTGGATGAAGATCTCTTCATTATCTTTTTATGATATCATCACAGATTCGACAAAACTGAGTTACATCGAAGAAGCCTTAAAAGAGCAGAGTGCTATTATTGGATCTGCTAGTTACAACAAACCTGTGTTTGCTGGAGCTAGGAGCCTGATAGTAGATCAGAAGAGGAAATTCGAGGAAATGCTTAAAGCTATTAACGATGTATCTAAGACAACATCTCGTCAAGCGACAGTGGGCGTTCTTATTCATGGTAAAGAAGGTATTGGTAAGAGTTCTATCTGCACCGAGTATCTTAACAACGAGATTGGTAAGATTTTTGGTTGGAATGGACAGATCTACAATGTAGACATGTTGAATTATCACGATCCATATCGTGGAGAAAGTTTTGGAATAAAGAACGAAATGTTTGCCATTAAAGAAGACCCATTCGTGCCTAAATTTACCAAGATATGTTCTTCGGACCCTTACAATTTCGAAGGAGCATCTCTATCAAATAAAACAGCACCATGCCATCTTAAAGTGCTTTTTATGACCACCAATGTCAAAAACATCGAATTGGGAGAATATCACAAAGATTGCAGAAGTGCTTTTTGGGACCGCCTCATACATTATGAAGTGGATGACCCCCAATTAGATCCGAAAGGAGGACGAGAAGGAGAAAACCCTCACAGGAAAAGAGATTTCTCTCACTTGAAATGGCGTATCAGAACACATAAGACCGAAAACAAAATAGAGTATTTATGCGATGAAGCAAACGTGCCTCTTGAGTTTACTACCCAAGAAGTCATGTGGAATATAATTCGAAGAATTATTAAGGCAGAGAAATCCTACTTGGGAAGCTTGTTAAACATCAAACCAGAGATGCAAGTTGAGATTCAGGAACGCTTAGATCGATTGAACACCCTCGGTGATAGACCTAATACAGGCGGTAGTGATTTCTTTGCTATTCGCTTTCAAGGCACCCCTGGATCAGGGAAAACAACAATAGCAAGGAAATTAGCTAGGAAATTGCATATTGCATATGCTTTTGAGATACAAGAATCAACATCTGTAGCAGAGTTCGAGCCTGTTAGAGACCGTCCAATGATTTATTTACTGGATGATTGGGTTAATAAGACCATTGAACCCCTCAAGGCAGAATTATTCATTGAAAAGACAAACCGAATTCATAAGAACTCAATATTGATTTTAACATCCAATGTTGAGTTTCAGCTTGAGAGTAGGATTTATAATAAACAACGATATCTAACTTTCGCTATCACTACTGGAGGAGACTATTCGAGTGTGTTACCATATGATTCAAGACCATACAAGCATTTACCAACTGGTATTTTGCGACGTATTGGATTGGAACTTATGATACGTGACCCTTCGAATAATAATATCATCATTCGACCACCACATGTAAGTAGAACTTACACTGTGAATCGTGTTGGTGAATTTATCAATCGAAGTAACAAGAGTATGATCTTCAACCAAATTCTAGATCAATCAGTCTCGGATTTTAATCAACATCTCTCCCGATCTGGAGACTTCCAAATTATCAAAACACGACCAGAAATAGTCCCCACTAATGTGGATGTGGATTTTAAGAGCAAGGATGCCAGAAGTCTGAAGAACAGCCTTAAGAATATGGCAAACATCATTAAAATGTATCGGGGATACGATCCTGATACTACAGTCTCCCATGCTCCTGATTTTTATAGCAACGAAGCATTTTGTGCTTCTGATTATGCTATAGACATGGATGCGATCCAAGGAGAGAATGATGAAGAACTGCTAGAAGCCGTAGCCTCTAGACTAGGACTTAATTTCCATCGGTGTTTCCCGGGGAAAACGTTCAGGGCTCGTTTAGAGACAGGAAAATCTGCATACATAGAAAGAGGACGATTGTACCTATATGATCAGGAGAGCACCAACCGAGTTAAATTCAACCATAATACAATGCAATATACTGGTTCAGGTCGAGTAGTATATGATATCACACCAGAAGATTTCGCACGAGCTCTTGAGAGCAGGAAGGATATGCCAACGCACGAGAGATTCATCGGATCTATGATAAATCTCAAATATCACGAATTTGATGAAATTCTCGAATACTATGAAAGTATGCGAGCTGATGGTGAAGAACAAGCTAGATCTTTTGTGGCCGAATGTGAACTCATGCGTTTTAAAAACCGAATACGTATTAGTCCTAAATGGGCTCTTATGTATGCCGAGATAGTAGATAATCCCGTAAAATACTTTGTATTTGGGTTACTTGCTATGACAGCAGTGGGAGGTACAGCTTTCGGTTTTTACGAGTTATATCAATACCTCCGTAAGCAGAAAGAAGAATCAACCACTGTAGCAAATGCGATGTACTACACTGAAACAACTCCATCGAAGAGACAGCAAGGACGTGACAAAGCTAGGAATGTGCGTGAAACATATGTTAATCAGATGTATAGCGCGCACTCTAGCCCTACCGGACGAATCGCAAAGCACGAGCGACGTAGAAATCTCAACGAGACGTACGTAAACACCTCGTCTCATGTTAATCAATTTACTCATATACAATCCAATCCTTCCAATAGGATTGCCAAACGTGAGCGCGAACGTAATCTGCGGGAAACTTATGTCAATACATTCAATTATGCTGGCATACATGAACATCAGTGTAAGGATTGTAATCAATGGTATAATCACACTCACCGTGGCGCTTCTCTCAACCATCGACAAATGAAATACGAGTGCCCTTATGAGAAATGCCGTAATTATTATGGCAATCATAAGAATGGGGAAAATTTCACTCAAGCAATCAAGATAGGAAGTCCAGCAATGGAGAATCCTACTATCGTAGCTATTCAGGATCGCTACAAGATAACTCAGACTGAGTTGTTGAATAGAATCCTGTATGAAGCTGATGATGATCATATGGATGAAATGTTCCACGAATTGGCAACAGGTGATTGTAATCTGGAGGAATTCAAGAAGGCAATACCAGAGTGTTACACCAATGTTTTTGAGTTTGGTATCAAACCAAATATGTTAGGTGTAGGAGATATGATTCGCCAGTACAAGGAGAGTCCGTATAAACAGTTCCATAAGTCAGTGCAACCACTGTATGCCCGAATGGATAATGCAAATGGGTGCAACTACGCTCAACATCTGGAACAAGGGCTTTTCCTGACTGTAGGACACGCTGTGACGGAAGTTGGAGAACTTTCAACTCTCCGACATTTGGAAAAGAAATACAACGCTCGTTGTGTTTATGTAGACAGGAACAAGGACGAAGCTATAATTTATTGTAGTGCTCTCGCTAATGTTATTCCGGCTGGGTTGAAGCGAATTGTTAAGACAAGCGAAGATATGCAAGCAACGTGTGGAGCTTTTATGCGTTGTGGACCTACGTATGAACTTCTTGTAGGTAAGATAATGCCATCATATGGCATCAGGATCTGTCAAGATAATCCACGTTATAGGCCCAACGAGTACCAAATCAAACTACATGCAGTAGGGCTAACCGATGTGACACACATTATACAACAGGGAGATTGTGGATTTCCTTTGGTTGCTATGATAGATGGACAAGTTCAAATAATCGGCTTTCACAATGCATATAACACATCAGTCAACGCGTATTTCACAGCAGTGGATCAGGAAAGATTACGTCAAGTTATAAGCAATGTTGTCATTACTAACAACTGTAAAATGGGAACACCCATGGGGAAAGTTCTTTTCCCGGAAACAAAGTTCCGACATGAACCTAGTAGTTTTGCATTACCAACCCCCTATGTTGATGTTTTCACATCTGAAGCAGCACCTACTGAAAAGTTTCAGGATGATAAACTTGATTGGCTTGGCTACTTCCGACCACTCCATTTTAAGAACAACTACAAAATTCCAGTGCATACGCACGATCTTGAACTTGAAAACCCGAATGGAACCCTACCAGCAGCATATACCAAACAATTTATAACGGATTATTCTGATATCAAACGTAACGCTTTTGGGCATTATGATCCGGGTTACACTCAAATTGTAAAGATAACACCAGGAAGGAGCGATTACGACCCAGAGTTGTTAGATCTTTCAGCCGATTTCGTACAAGAACACATGTACAGTTGGTATGGAACGTGTTCGTTTTTGCGCGATCATGAAGTGCTCAATGGATCACCCGATGGATTCCTTAATAAGGTGGATTTGTCAACAAGTGCCGGACCCCTTATGAAACTCATGTACAAGGTGACCGATAAGAAACCTTTATTTGACGTTCGTGAAGAGAAAGGTCTCCACTTTATTCACTGGAAAAAAGAGTGTGAAATGAGCCAAACTGTACGATCTTTGGCGCAACAGACATTCGACATGCTATTAGATGAGGATCCTACTAAGGTTCCATTAATAGTAGCACAGGATTGTATGAAAGTTGAGAACATTTCAGCAGAGAAAGCCAAGAAAGGTAAAGTTCGAATTTTTAACACTGTGGATCTTCATCACAATCTTGTGATGAGGAAACTCTTCGGAGATCTAGTGAAGAAATGCAACAAAGAACACGAAACAGCATGGTTTGCTATAGGACAGGATCCATACAAGACATCAACCAAAATTTACAAAGATTTCCAATTGATAGACGGTACACTAGTCAATACCGACTATAAGAACTACGACAAATCGATTCATGCTATACTTATCAGGAAATTTGTGGAGATATTCGTGGGACTCCTTGATACAGAAACGAGGAAGAAGATACCTAACATCGAAGTCTTCAAAGACAAGTTGGCGAAATATCTCACCAATACCGTGCATTTGTATGATGGGAACGCGTTTTTCACTACGCAAGGCAACAACAGTGGAGTATATATAACAACTTTACTCAACTGCGTTGTTAACCACATCCTTACAATGTACAGTGTTATCCGCAAATATCGACAGATGTATAACATGACACCGGTGCTTGTAGAGGTGCTGCGACAGTTTGTTGCAAGATATATGGGTGATGATCGCACGCTTAAGGTTTCTCACAACTTACCTCTCACAATGGAGGAGATTATCCGTGATACTGCAGAGTTTGGGATGTTGTGCACGCCAACCAAGACACGAGACGACGGGACGAATAGCGAAGTAATAGACTTTTGCTCACGGGAGTTCTTCTGGGATCCAATCAAACAGGTTGTTTTCCCGGGACTGAAGAAGTCGAGCATCTGTGGCCTCTTGTACTGGTTTAAGCACAATGACAGACTCCAAGTAATTCAAAATTTGACATCTTGTCTGTATGAAGCTGCCCTACATAATGACAAAAGCTTCTTCATGTCTATTTTACGAGATGTGAGGAAAGTCGCTGATCATTATGGTGTGAGTTTGACGGAGTTTGGATCCGGTATACCGTACACAAACTTTGAACTCGTCACACGAAGGCACGCTGATGCAGCCCTCGGAATTTCTATATTATCGCAGTATAGTACGCGTGAGTTGGATATACAAATCGAAAACGATAATATATACAAGCGCAAGCTAGATATTTCTAGTATCACAGAGACTCACAGAGAGATCGAATACAGTTTGAAAAACGCAGAAAACGATAAATCAGAACTAGACAGATACAAGCGAAAACTTGCACAAGACTATCTTCATGCAATTGAGGCCGAGATGGCTTTATCTATTAGCGGCAATCCTATTTCTAGGACTCTTGAGCTTTTGTCTAAGCTTAAAATCACTAAATCAGAAGACAGTTGTTGTCAAGTCGGAGGAACCCCCGATAGCCCGACTTGGGAGTATTCTCTTACCATCGGAAGATAGCAATTTACAGGGCGAGGCTCAAGTAAGAGTCTGGCG

>Spalangia endius iflavirus

TGAACTCCAAGATGCAGCCGATTTTGTCGATGCACTAAAGAAGGGTACTGCTCAATCATATTTGAATAATCATCCGAGGGCTAGGTATGCAAAAGACTCAGATTGGTCTGAAACTAAAAACCCAAGATCCACTGAATGGACTAAAGCTGTAAATGATTCGACAGACCATCCTATATGGATTAAAGAAACATCTCAGCTTATGAATTTCGAAGTAGTTGGTCAGATGGACACAGAAGGTATTTCAGTGCGAATCGAACAACCAGCACCTACAACCAATTTTGGTGAACATGTGTATGGTGAGGCCATCCCCGATTTAAAAGGGATGGGTAGGCGCTGGCAGCACTATTCCTCTGTTACTGTGAATACTTGCACGAAACAATATCCTCGTGATTGCGGGTATGCTGTTAAAATACCTATTCGGCCAAATCGCCAGTTAACATCTTCTGGATCATATTCATATGATAACCGAGTTCGTGATGGTGTTATACCATTGATATCTTCAGCGTTTTATCTAACTGATGGAGGTATGAGATATCGTATCATTCCTATTAAACAAGTTCCGGATGATACCATAATATATGTACAGCACCGTTTTGATGATACCGAGAATGTCCAAGGTCCTATTATAGGGAAAGGGGCAGTAAGTGCTCGAGATATGTTGGATACACATTATGCTACATATGTTCAAGCCTTGTCTGTTAATCCCGTAGTCTCTGTTGAAGTGCCGTATTATCGAGAGACAGAACGCTTGTTAACCACATTTAATGAATCAGGGAGACCATCCTCGAACGGTTTTCTGTATATTTGGTTACATTCAGCGCAGAAATCTAAAGTTCAACTTGAGATATATTATTCTTTAGCTGACGATTTTAATTGGTCTGTGTTTCAGGGATTTCCTCCCTTGATGGATATAACCCAAATATCAGATGAGCCTACTCCTCAAATGGATGTAGAACCTGCTGAAGAAGCTCTCCCACAAAGTTGGTTCCAAAAAGAACGGGATAATTTGATACATTCAGCCGATGTTATCCGAGAATCTTCTGTTAAAATAACGACTTCTATAGAGGATGCCACAGCAGTCTTTACAAACATTGCGAAGAAGTTCGATAGTTTTATTGATTCTGCAACCTCGTTACCTAGTCGAATTTCTCGGTCTCATAATGATATGATGAAAGATCCAACCTATAATAAATCTTGTACAATATTGCAAGAGTATTTGAACGTGGGTTTTAATTTTATATCCCATATAGTACCTTGCATAATATCACCTACACCCGGAATAATATCCTGGGCTTTAGTGTGTTTGTATAGAGAGATCTTTGGATTTTCCTTAGAAGGATTTCAACCATTGATAAATCTTTTTTCCAGTTTATGGACACTCGTTACTGGCCCCTCACAACATCCGCAGGGTCAGATGGACGACGATATAGCTGGACAGGTTTGTAGTGTCATGTTCACGTCTTTATGTTCAATGGCTAAACTTTCTGTTTCACCTCCTACATCGTGGAAAGATGTTACAAAAGGTTTATTTGAATTTTCTAAAGACTGCAGAGCAGGCTCAATGGTTGGTCGATTCTTTAAGGATAATCTGGAATTATTACGTCGGGTGTGGAAACGCATTCTTCGTGTTTTCTCAATTGGAACTCCGCACTATAAGATTATAGCGGGAATTGAAGATGAGAGATTGCGAGAGTGGGTTGCCCATGCTTCAGCTATAACATCACCTACTGTGAAAGAACAAGTCTATACAAATCCTTATTGGGCTGAGAAAGTTTTTGAATTAGCTGTGATGGGACGAGCTTTCCGTATATCAATGGCCAAGGAACAAACTAATTTACCAGTGGCTTTGACTCGTTTGGTAATTTCATTAATAGAAGCTTTACTTAAAATGGAAAGAGATTTGGCTAATAGGAAAGTGTTTTGTGGGGAACGATATGAGCCATGTTGCATTTGGATATCTGGAGCAGCAGGAACGGGTAAAACCCGATATCTTCAACATCTTGCTAATATTTTAGCTCAAGAATCCGGATCCAAGTGTGCTCATTCGTACCACACAATAACTTTTAACCAACAATATTTTGATGGGTTTATTGGTCAAGATAACATTTTAATGGATGATTTGTTGACAACTTCCCCAACATCAGATCCGGCTGTACATATGTTCATCCAAATGAAATCATCTGCTTTATTTAATCCTCCCTTTTCGGATGTTAAGGATAAATCAACAATGGTTAATTTTAAAAATTTGTTGGTTTCTTCTAACTTTTCGGGAGTTAAGAATATGGCGGGTATTCATGACGACGCAGCGTATAATCGGCGAAGGGATATACTTCTTCGGTTTGAAAAATCTGGTTTAAAAGAAACATATACGGAACAAGAATATGTGGATTGTCTTCATATAAAAGCTTACCACCTACCTGATCCATTAGATCACACATCTAAAGGTGTGCTTATTCCACGCCAAGAAGGAGTTCACTATAGAAAGACTCTAGATGACTTTATATGCCAGCGTACTCAAGAGTATCACCTCAAGGAATCTGTCAGTTTTTCTCGACGAGTTCGAGAGAAGATGGAAAGATTAGATTCCATAGGGGCAAATGCTCACTCTCTTGATGATTATCTGGAGAAGTGTACAAAGTTATTGAATTTACCTAATCGTGAACAAGGTTTGAGAGAATATTATTCAGGTTTACAAGCTTGGATGGGTGATCATAAGTTACCGTCTGTATCAGGACAAATGGACGATCAAGAAGTTTATTGGCCAAATATGTTTACAGGTCAGTGGTCTCAATTGGCTAGAGTAATAGGTGACAAAACTTTAGAAACCTGGGCTGAAGCTAATTGGGTTGTTAATCCAGCTGAAAGTGAAATCCTGTGTGATCCTATCGAACCGTTATCCCGAAAAGCCATTGGATGGGAGCTGAGGCCTACTCGAGCAGATAATAAATGTCTGCATGAGGTTGATAACATAGACTGTTATGCATATGATGATATGTTAAGATTTTATCGTATCCTAACACCATCTATGTATGTTAATATGAAGGAACCGACATCGTTCCCGGTTGGCCAGTGCCTATGGAAGAATGTTAAAGGTGAATTAGAACCTTTACCTAATTGCATTATGAGAGATCCATGCAAGCGAGATATGTTTATTAGTCGCTTGGCAGCTCATTATCTTCAGGATAATCCTCAATTGAAACAACATATTATTGAGAATAAAGGATTAGGGGATATGATACCCGAAGATTTCCCTAAAGAGTATTGGCCTAAGTTAGAAGCCTCTGTTCGACATTTTAAATCTTATGTCGATTATGTTGATTCTTATGTCACTAGGAGATTTGCTAAAACACAAGCATCTATGAGTGAGAATGTAACAAATGTTGGGGATATCTTAGTGCCTAAACAATCTAAATGGAAAACTATCCCGCAAGTTGTGTGGAAAGCATTCTTAGTGGTTGCTAAAGGAACTTTGAAAGTCCTCGATGTTGTGGCGACTATAGTTAATACTCTTCTGGGAATATCTTTGCTAGGAGCCGTTGGGGCGGGGGTATATGGAGCGATTCAAGCCAATAGACAACAAAAATTGGTTAATCATTCAATACCCATCCCTAACTTACATCCTTCAGGGGATTATAAGACTGTCAAGAATCGTTCCTCAGCCCGGCAAATGGCTTTATCCCTTAACGCACCTAATAATGGTGGTCCTACAGATGAGGAGCTAATTAAAGCAGCCGATACACAAATTCTCGGTCCCTCGGAAGATGGAAGGCTAAAGAAAATTCTCCGAAACACGTTCACTCTGGTTGGTTATGTGGCAGATGGTAATGAAGATGAAGTTACTTATACCCGTTGGCCTGTGAGATGTATAGGATTGGAACAAAATAAATTTCTGGTTCTAAAACATTACATAGAACATTTTCAATCTCAAAACGTTCAAAATGTTGCTGTCATCTATAGGAAAAACTCAGGTTGTCAACGTTTTCGTTTGGACGAGATAGATTTTAAATGGACAGACCAAGGGTATGGAGTTGGTGAATTTCCTAGTTTAGCTCAACCTTTTGCTAATATTACGAAATTCATACCATCTGAATCTTTTGATGGTAACTATCCTTCAACTATGATTATGGTAGAACCTAAAACAACTTCGGTTCAATTATTTGAGTTGGAAGTTACCAAATTGCAACAACCTAAATTTGTGCCGCCGTGTGGGTCCCAATCACCTTGGACTATATCTCAAGGTTTTAGTTATGAATGGGGAGGAAAAGGCAGATGCATGTCATTCTTATTTGCCCCCACTCTAGCCTGTCCTTTGGTAGGTATCCACACAGCAGGTGTCTATGATCGTATAGGTTTTTCCGAAATTCTATTGAAGGAAACCTTTGTCGATCCAGATATACCAGCTTTGGATTATGTAACACCTCATATGGCTATATCAGAGGATGGATTTGACTTACCAGGGGATGGTGTGGTTCTGGGACACGTTGACAAATCGGAAGCTGTCCAAATGTCTAGGGACACTAAGATTCGTCCCTCAGATATATCGGGTGTCTTTCCTGAGAAAACGGAGCCGGCTCCATTAAGAGCTTCTGACCCTCGGCTTGAATATCCAGCTGATCCATTAGTTGAAGGTATGAAAAGACGTTGTGATAAACCTTTAGAGTTACCCAAAGGAGATGTGCAGGCAGCTTATTTAGATTATGTTGATCTATTACGGCTGAATGCTAGACCTCTAAGAACTCTAACTCCTCTAACAATAACGGAGGCTATTGAAGGTTTAACATTACCAGGTTATGAACCTATGACTATAACAACCTCAGAAGGATACCCATGGGTTTTGGATCGACCAAAGGGAGAATCAAATAAATCTTGGATGTTTAAGTTTTCTAATTATCCGGATGGTCGTCGGAAATTGGATGGTATTTATACACCACTATCAGATGTCATTGATTTGAAGAATGACATGTGTAAGAGGGATATAGTTCCGTTCACATATTTTACGATGGCATTAAAGGACGCTAGAATCCTTAAAGAAAAGGTTTCAATACCTGGTAAAACTAGAGTATTTGAGATGTCCCCGGTCGATTTAACAATCCGTTCCCGACAACATACTTTGGATTTTAATGCAGCTTATACTAGCCATAATCGTTTAAGATGTGAGAATACTATAGGAATTAATCCCGATGGTGAGGAGTGGACAATGTTCGCTGATGCACTCCGGGAGTTTTCCCCTTATATTCTAACAGCTGACTATTCTGGCTACGGACCACGTTTATTGCATTCTGTTTTATGGAAACATGAACAGGCTGTAATGGCGTGGTATTTGCATTATCAAACTAGACAAGGTATTGATGAGGAGGTTATAGACGAAGGCTTCCATATTCGTTATGCCTTAGCTAAGGAAGCTCTAACACCAGCCCACATAGCTAAAGATGCAATAGTCAAATTTCCAACTGGACAAGATTCAGGTCATCCTAGTACAATTCAACGGAATTCGGGAACCAATTCAATTATGATAAGAACAGCTTATTTAGGCATTATGCGTGAAAAGAAACCGTGTTATGCCGATATGTATTGGTTCAAGAAATTCGTTTTAATGTTCACGAATGGAGATGATTTTATAGCAGCAGTTAAACCTGAAATAATAGATTATTTTAATAATAATACTTTGATAGAGTATTTTGCAAAGTTTAATATTAAAATGACTGATGCTTTAAAATCAGGAGTACAGCGTCCCTATTGCTCTTTAGAAGAAGCAACATATTTAAAGAGAGGTTTTTTACGACATCCAACTAGACCTGGTCAATGGTTAGCTCCATTGGATAAGAATTCAGTTACCGATACAGCAAATTGGATTTGGTTATCAGCTGATTCCAAGCAAGCTTCTCTGGTGAATTCAGAAATGTGTGCTCGTCTAGCGTATACTCATGGTCCAGAATTTTATGACTATGTGGTTACAAAGCTTAAGAACGCTTGGCTAGATAAGGGTGTTGCGTTTTCGGCTCCATCTTGGGATTCACTAGATCAGCATGTGTTTGAAGGGGCTCCAGGCCCAGTGTTTAGCTTCTGATGTCTTCCTCTCTCCCTGGATTAGACGTTCTCGGGTGTTTCCGGCCACTAACATCAGTGGCGCTGTTGATCCCCCGATTCTTCCGTTATCTCCGTGGTACCTCGCGACCATCCCCCCCCCCCCAATGCACTTCCCTGGTGCATTTTTCGCGGGGGGAGAATGGTATTACGATCTACCACCCAACAACTTGGGAGCTCTGCTGCGAACGGAAGAGC

>Spalangia endius hurwu-like virus Segment 1

CTTTCATGTGTCAGGACTTAAATTTAGAGAAATTGAAACAGATAGGAAAACAGCAATGTCTGATTTTACAATTGCTATTCCTTTAAATGATGGAACTATTTTTCAATTACCTCCTAACACTCATAGGTGCAGATTTCATTCAGATCCTATTACTGTACCAGGTGATGGTGATTGCTTTTATCATAGTATATTAAGATCAGTTACTTTACAAGATAAAATATTAACAACTAATCTGAGACACAAAATCTTTAATACTGGCTATAATAGTGGGGTGTCAGGATCAAGTTTAAGAGAATTGGAGCAGGAAACCATACTAGGGGTACATGATGGGAAGAAACCATATGCTGGTAGTATGGCAGTATGGTGTTGTGCATATACTCTTCGAATGAATATCTGCGTCCATTGTGAAAAAGAATCTTACTGTCTAGATTATAAAATAAATGATAGTTATAATACAATACATATATCATATAATGGTAGTCTTAGTGAATCAGGAAACCATTATAATGGTTTCAAACAAAGTCATCTGCGACAACAGTTATTAAAACATTTCATGGTCAATTTCACTAATGATACATTAAATATAAACAGGAGGAATTTTAATCATGACTTGAAGAGGTATTATGAAGTTCCTAAAACTGAGGACTTAGTATGGTATGATAATCTAATAGATTTGATAGATGTTAAATTACGCAATAGATTATATCATGTTGAATCTCAAAATGATCTCAGATTTACATACTTTTTAGAAAAGCTAAAAAATAGAAATTTAAACACTTATGATTTGGTAGAGACCTTAAATCTGAGGTTGGGTTATTATAAATATTATGGAATCCAAAATAATGTTGTTAAATACAAGTTACATGATAGTGAAGAATCTTCTTATGTTAGTAGCAATATGAAAAAAATAACACTGATAATATTGAATGATGAGACAGGTACTAGATGGGCTTGCAATCAGATAGAGCATCCAGATGAGGCCTTAACTTTCCAGAGTAGTTCCAATTTATATACATTATTTTATGAGGCAATAATTGCTTCAATAGTATCAATAAACCAAGAATTGTCAGCTGAATCATTGACTCATGAATTTTTATCTTATGTTAGAGAACATTCTCTTGATTATGATATTGTGGGAACACAATTTTATTTGGAATCATTAATATTATCCCATTTATCTCAAATGCCAATATTAGTTTATAAGAAAATACCCAATGTCAAATATAATGTGAAGATGTTTGATGTGAATAGGCAGTTTAATACAAATCCAAGTGATTGTGTATTTATATTGTGTGAGGGATCTAATTTCAGATCTGGTACTAAATTATCTTCATACATAATGGAAGATAAAAGAATCACAATTTATGATTATATACCTGGCCATGGGAAATCTGAACAGACTGTATTGAATTATACAAGTCCTAAACTAGTAAGTTGTGTGCTTGATAAATATTTAAAACCTATGGGGTCAGATTTGATTAATATACCACCTTTCCTTACTCACATTGTTAAATCAAATCATGAAGTTTGTGATTTTGATTACAATGGGTTAAATTCTAAAACACAGCAATATAGTTTTACCTTACATCCATTAAATGAGCATCCCATTTCCATAATAGAGCCTGAGTTTACATGGAATGTTGTAAATTTTAATTCTTTTGTGCATGACTTCACTTTTGGCCATTTAGTTTCAACTACTGATGTATCTTTCAGAGATATGAATTTGGTCCTAGGAGATGAGGACGATAATAAAACTCCAGATTATATGACTATTAAAGGTGATCATTTAATGGTTGTGGAGTTCACTACTAGAGGGTTCTCATCAGAGAGCATTAGGAAAAAGGCATTACAGGACAAATGTCACAAATATAAGCATATAATAATGGAAAGATGTTCAAGATACAACCTAAAAGCAACATTTTTTATTATATGTGTTACCCCTACATCGATTGAAACCAATATTCCTAATAATTATTTAGATGATAAATTTAAAGATGAGTTAGTTTATAGATACAGAGTCTCCAGTGCAATCTATAACACTGTTATTGAGGCAAATGATTATCAAAAATCATCTGATGAATTAACTAAATCAATGACTTATGTCAAGCAAATTTTATCACAAATCAAGTTACAAAGAGATTTAGTCACTAATAAGATCAGTGACAAATGTTTAGAGAACATCACTAGACCCCCTAATGAATTTGATAAAATACAAACTGAGTCCTTAATTGAGTGGGCAATAAAAAAGGGAAAAGAAGATTTATGCAATCAATACCCGGAGAATATGGATGAATCAGCCAATTTTAAAGATGTTTTATATACCATAAAAGCTGTGAATCATGATAATGATGTTCAAAATTATTATGGCAACTTCAATTTTAATGAGGTTAGAATGGATCCCAAAGCAATCATCCAAATTCCTTGGCCTCATTTAATTAGTAACTACAATAAGGATAGTAGTTTAGGTGAATTGCAAGAAGCCTTTAAATATACTGCCTTATATGGTGATGAGACTGTTCAATTATGGAAGACTGCTATTATGGATCCAGTAAAAGTGACAGTTACTCCAGATAAGGAAGTAGAAGAGAATGATGATTACATTAGTGAAATTGAGAGAATAGGAAAATCTCCAGAAGAACAACAAGCTATAGATGAAGAGCAAAAAAAGTATAGAAAATATCATGGTAGGCATAGATTTACTCTTAAAGATCAAGAAATATTAGAATTGTCAAAAATAGGTTTCTTAGGTAAGTCTAATAAAGACAACCCAACTGTTATAGCATATCGTAACCAAAAGAAGAAAAGCTTCTGCCCTAAAAATTCACCCACTGATGACATTGAACACTTTCTTGGGAATCCTAAGGCAATAATTGAAAGCATGAATTTTGATAACAATATTGATGAAAGATCTTATTCTATCTTACAATTGATAAAACAGGCCAGTTCAATACATTCCAATGTGGAATCAGTAACCATGTTAGAGATTGTTATGCAGTATATTAAGTGTGATTTTGGAGTTTATCTACGCTTTGTGAGTGATCTAGCAACAGAGTTAAGTATATCATTAAAACAACATTGCAACCACAAGCAAGTCATTATTAAGAAAATCTCCTATTTTAATGTCTATGTGTTGATCAAACCCTCATCTCTAACTGGTAAGTTATTTTATTCATTAGCATGGTTGAAAGAAAAGTCATCTTTACATAAATCATTATACGGTAATATTTTTAAGGAAATCCGTGAAGTTAATGGCATCTATTATACTGAATTAGTGTCTACTAATATTAGTAAATTAACTAATTTATGTAGAGCTGAGTCATTAGGTCTAGCAACATTAGGATTTTGGTTGGAATTTTATAACGTACCACTATGGAGTTCTAAGTCTCTCAAAGAGTCATTTTTAGAAAAGCCTGAAGTTGTCAGTATGTGGTTCTTTAGTATGTTTGTGGCTCTAAATGATAAACATACTACTGAAGAATTAATCACTATGACAAGATATGTTCATATGGAGTCATTAGTATTATTACCAGAGATTAGCAGACCCTCAAAAATGATGGAGAAAATGCCTAAAGTATTTAGGTCTAGATTTGAGGTGTATATTTTTAAGAATATTATACGATTGATGAAGAATTATTGTATGAATCAACCTACCTTATCTTCTGCTAGTATAGATAAAAGGAGATGGATTGGATTTGAGAATCCATTTCTACCATATGATAGTTATTGTAGTAATTCATTGGATAGTCAAGATCAAGTTCTTAATCTATGCTATTTAGGTTATACTAAGAATAAGGATGAAGATCCAGAAGCTAATTCATCTGGGGCTATGTTAGCAAAGATTCTAGGATTAGAGGATCAGTTTCCGAGGAAATCAGATGGATCTAGAGATAGACAATTCTTAGGAGAGAATAATCCTAATCCTGATGATATTAGGAAACATGAGTATAATACTTCATTCCTAAAAATTATATGTAAAACAGCCTTAGATAGAATATCTAAGGAGAAAGGTGTTTCAAGGCCCCAAGAGTATTTGGCTGATAGAATACTAAAATTCATGACACATCAGAGCTTGGATGCCTTTGCTACATTGAAAGCATCATCTAACTTTTCTGAAGCATTTTATTTGTATCCTTATGCATCTGGTGTGAAATATCATAGATCTAAGGTATTAGAGAAACTACTAGATGTCATTTCTGGAACAAATGAGGATGAAAAGAAAGTTTTGGTAGTAGATTATCTACAAGAGAGTTTAGATATAATAGAAGAGCAGGGGTGTTTGCACATTTGTATATTCAAAAAGAATCAACATGGTGGTCTACGTGAAATTTATGTACTAGATATTTTTGGTAGAATAATCCAAAAGTGTCTAGAAGATTGTGCCAGATCTATACTTGGAGAATTTCCAACAGAGATCATGACTCATCCAAAAAATAAATTTATCATTCCAGAAATGCATAATTCTCAAGCTAGAAAGGCCTTTGGTAATTTATATACTACATACTCGACTAGTGATGATGCAGCAAAGTGGAATCAAGGTCATTATGTTGTTAAATTTATGACAATGTTATGCACCATGTTACCATCATATATGCATGGATTTATAATAAGAGGACTTCAACCATGGTTAAATAAAAGAATCAAAATTGGTGATGATCTACTGGAGCTATTCACCAAGGTGAATGAAATGACATCTCAAGACAAAGTAGTACAATTAATATATGATATTTTTAAAGGAAAAAAATCAACAGAATGGATGCAAGAGTATAACACATTCATTAGTACTGAAACTGGTATGATGCAAGGAATCCTTCATTATATCAGCAGTTTATTACATGCTTTGTTGCTAAATTATGTTTCTTCATTGAGTGTACAATTATTATCAGCTTGTTTAACCAATCATACTGGCAAGTATGTCAGTCCCATTGTGGATGACATGGAGAGTTCTGATGACAGTTCTTTGATCATTAGTGTCCCAAAATTGGATGATAAATTTGATTGTTCCATTGCATTTAGTGTAGCTTTATGTTTCCGCATCAAAACTCTTCTATCAGAACAATTAGCTATATATAGATCAATAAAGTCAACTTCCATGACCAAATTCATTATGGAATTTAATTCTGAATTCTTTTTTTATGGAGATGTTCATAGACCAACATTTAGATGGGTCAATGCTAGTGTGTTAATCACTGAACAAGAGAACCTGATGGCAAGACAAGAAGAGATGTATAATTTAATCACCAACATAATACAAGGAGGAGGAACATTGATTTTATCATCTATCTGCCATTTATCACAGTGTCTACTACATTATAGATTACTTGGTTCATCAGTGAGTGATATATGGCCTCTATATGCTATTAAATTGAAAGATTCTTGTGATCCTGCAATGGGCTTTTTCTTAATCGATAACCCATGTGCTACAGGGTTAGCAGGATTGAATTACTTAGTATGGTTGGCCTGTAAGAAAAGTATATTAGGACACAAATATAAATTGATGTATCTGACAGAATTAAAAAGTACCGATGAGAACCTGGAAAAGATTAAAGATATAACTATAGAGACAACTAACATTGGATTATTTAGTAGGGCAGCTCAAGTCTCATATGGACAAAGTACAAAATGGAAGTCTATTATGTCAAAATTTGGAGAAGTGGATGATTATATGGATGAAATTGATGAAAACCCAGAAATCTTATATGTAAAACCAACAACCATAAAGGAGTTGAAGTTGAAATTATTGGTTAAAATGAAATCACCTGGAGTTATTGCTAGTTTAGGGAAAACTAATACTTTGCCCAGATTAATTGCTAGTAGTGTATATATATTGTCAAGACCAATTTTAAAAACTAATTCAGCCTATTATAAAACAATGCATGATAAATCATCTCAGAAATTAACTCTGATTTCAGACTTATTACTAAGTTCTAATATAGAAAATAGTTATTATTCTACATTTCTGAACAAGGATACATTTACTTATGACCAGGAGTTGAAGGTGACCCTTATTGATTCAGAAATCACAAATGATACAATATACTCCTTGTTAAGAATTATCACAAATGTTATACCAAGCATGCAAATGGTTGATTCAAGACTAATTATAAATGGTTTCAAAGCATACACAAGATGCATGATAACCAACAGCAGTAGTATATCAGATATCCTATCCTCATTATCTGGTTATTTATGCTTAAATGTAGTTGAGTGTATTAGAACTTTAAACCATGATGTGATCAAATATCGAGATGAATTTCAGGTGGTGAATCCACATTTGAGAATTGCTTGTGTGGTTGAATCAGACAGAGTATTTGTGTATACAATTAATTCTAATCCTATTTATGTTAATACAGATTCCATTCCAAAATTCACTGATTCTGAAGAGATCACATTGTTCCCATATAAAAAAGATTATGAACAATTATATTCCATATTAGCTCACATTAACCTGAGGAATGGAACTGCTGCAATTAATTACAGAAGGAGACAAAGAGCTGATTTAGTAATAACTGGCCAGGATGATGTGGGTCAAGTTAAACTGGAACATGTTCTAGTGTGGAAATGGTTTGGACATAATCCTAATAATTATTCAATAAAAACATTGGAGATAATGTTTGAAGAGTATTGCCAAAGGATACCATGGATACATCCTGATATACAGACCACTTTAGCATCATCACCTTTTACTAATCACATTCAATTGCAAAATTTTATTTCTAGACAGGATAAAAGCCAAAGAATAGTCCATTTAGTTGGTTCCTGTGGTAGGAACAAGTTTGGTCTAACAAGTCTCATGATGGTAATTAAGCATAATTACACAAATGGATGTGTATTCAAAGATGTTGTTGACAAAGACTCAGGGGTGAGATTTAGTAATTATAATACATATATGCACATATTATCAGAAATGAGTCAATTGCCTATGACAGATGTCTATAAACAAACATGTGTTCATAAGCTATTACTTAAAGGACCAACTATTCCATCATCTTTAGCTTATAATAGTAGGATGAGTAGAATGAAAGTTATTCAAGATTTTGTCACTTCTTGTCCAGATTTACAACTCTCTTCTCTCATGGATAGGTCAATCTCAGATATAAAGAAACAGAGAGATCAAAGATTATATAAGATACCAACAAAGAAGGAATCAGATGATATTGCAGATTCATTTTATGAATTACTTATTCAGAAAGATCTTGATCTAGAAACAAATCCAACTGAGGTTATGGAACTTCTTAATAAGTCCTATACTGGAATTTACAATTTACTTATTAAATTTTATTTACATGACACTAGGGAGGAATATTATGAAAATGCATTGAAATATTATTATGATATAAACAATATTCCTGAGGATGAGAGGAATTTATTATCTACAATTAGAGGGAAAAATGCCATGCTCCTTTACAAAGAAGGAATCATATCAAATATAAGAGATTATGAGTCTAAGCAATATGAATTTGAGAAGAAGAAAGCAGAATTATTTAATATGATAAGAACCATGAAATTGGGAATGTATGGTTTTTATCTTAAAAGTCAGAAGTTTGACCCTAAATCTCATTCCTATTATGGTGAGGGCATCTGGAGAGGTTATTTTGATGATATTGAAGTAGAAATAATAATTTATAGTAAAAAAACTGATAGTTCAGTAGAACTCAGGGAAGTCACCTTAAGTAGCTCAAAGAGAATAAACACTAGTCTAGCTTATCTAAAGAGATGGTGCTCTGACAATAATGTCATTAATACAGGAAAAGTACCAAAATCATTAATCAAGAGTAATGTTTATGGTTACTTATTAGATTATAAAATAGCCAGTATGTCAAGAGTTCCTTTATGTATAAATTCTCAGATGATACCAGTAAGCTTTAGAAATATCACTCAATTCAAAATTGAATTATCAAATAGAAGCTTGAGATTACTGGGATTTTATAAATTAGATGATGTTAACATGGTTGGATCTAGATGTGTGACAATTGTTTTTGTAAATTTATCCTATCATGATATCATACCTAATATTAATCACACCAAACTGGACGGTTTTGAAGTATTGGATCAGTTTAGACCAATAACTAAGGACTGGGTTAAACACTCAGTAGGTAACCAATCAATTCCTTTAATAATAGAAGATCTGAATAAGAAAGATTTTGAGACAAGCTTACCCATACAAAGGTACATGTTACCAAAACAATATTTGGAATTATTACAATTGCTATGGACTTTTACTACTACGAGATATTTTGGTAATCCCAATTATAGATTAAATTATGAATCAAAACAGGATCCTGAGAAGAAGGATAATAAACCTATGACCACTGATGACATAAGGAATTTATTTGTTTCAATTAATGAAGGTGTGTTAGATGAATTCAGAAATAGTGGTTCATTGATTCAGACCATGTTAAATGAAAACTTATTATTTAGTACATTAGTAGCAAATATACCATTTGGTCAAGAGGAAACAGAGCAAGAAGTGAGTAAAGTTATCAGTATTCACCCACTATTAGAATCTTTAATTACCATGATTGGAGCTGAGAATATTCGGTTTATATCAACTTCTATTAAATCAAAAAAGTACTTGTTACCAGCTGAATTAATAAATTATAAGATATTATTTTCCAATTTATATTCAATTCCTGAAAATGAATGGAAGATAATGGAAAAAACTCTCAATAAACCTGCAGTTATTGAGACTATAACTTTTGATGATGATGATTGAAATATATCCTCAATCATTCTGATCTTACAACATTTAAAAGAAAATAAGATCACAATTAGTTTAAGATCACTATTAGTTTATATGTAAAACAGTTCATTAATATATTTATGGTTAGCAAAGTATTAGACATCTGGATTCTGTTTATATTAATTCTAATTGTCCTGGAC

>Spalangia endius hurwu-like virus Segment 2

GGATAAACCATATATACATGTTAAAACAGAATCCATTCCTGTAGGACTTTTACTAGACTTCTTTTTCCTCTTTCCCATATCCTTATTTACATATTTCCATTTGTATCTTATGAATATAGTATTTTGGAGATGGCTTTAAATCTATTCTCTCGAAAGAGCTTTCCACGACGTGAGTACAGGTTGAATCCTAATCATGGCTTACCAGAAGATTCCAAAATACCTGAGATTTCAACAATCTTAGAACTTAGTGGGACTTCTCAGTTAAGACGTCCTGATCACATAACATCAACTTATCAAACTCAACAGTTCTCAATAGATACTGATGAAAAGCAAGCTTATGCCATAGAACTTGGTAAGATGAAAATGCTTAGAGAGTATTTTAGTATTTCAAGAGTTTCCATAGTATTCTTTGGTTTATATCCAAAAGACACAGGTATGATCACTTTGAGATTGAAAAACACTGCCTATAAGGAAGAGATGAACCAAGTTGAATTCTCATGCACTGGTGGTACCAACCGTATGTGGGGAATGGTGGCTTCAATGAATCATGCAATTCACAAATCAGACATTGATAACTTATATATTGAAGTTATTCTTACTGGCACTGGATTGCATGGAGTGATTATAGGTGAGATTTATGCTTTATGGGAGTTCACTTTTACTGATGTTCCAATGTTGTATAAACCTGAAGAGACTGTCTCATTTGCATTTTCACCTTTGGATAATGGTGAGAATAATTCTACTATGGCTAAGAAGACTTGGGACCTTTATAAGGCTATTGCTGATAAAAAGAAAGCTATGAGTTCATTACAGTTAATCCTTAATAATCAGAATGTAACAGTGGAAAAGGAGTATTCATTAAATTCTTCTGAACCATCGGCTCCATCACTGTTCAGAGATTACAATAAATTGTATTAGTTGTAACGACCATGATATTATCCTTCCCCTAATTCCCATTTCCTTGGGTTTCAACTTAAGTTGTAACCTGCTCCTCTCCCCACAAACAGCTACACTGTCATTTTTGTGGTTTAAAATGATTTTGTAACAAAATCATGAACTATTATAGACTAAATGGAAAAATAATAAAATATAATAATAATAATAAAAGGAAATTCAAATAAAATTTAAATGTATAAACTATTGCTTGAGGTCCAGGAAACTATTATGAGATGGTTGTATTAATTATAATAGAAAACAAATTAAACACCATGCTCCAATTTTAGTAATTCTTCACAGACATGGCTTATCCATTTAGGTAAGTTTAAGACTACCTTAGCATAATCAGGATCAGTTGATTTTGAGTCTCTACAGATTCTAGTAGCTAAATTACATATGATGACCATCTGATTATGAGTCATCCTATCTGGCTTCTTTAACATGTTGATTGTTAACCATGTACCTGCATCTTTTTGTGCACGGGAAAAGAATGCCAGATAATCCATTGGTAGAATTTCTGATTCATCTAAACCGACTAAGATGTTATGTGCCTTTTCAACATAATTAGGCAAGTGTCTATGGACTTCATCATATAAAGGATCATTAAGTCCTTTACTTTTGGAGATAATATCATAGATGTTTTCAAACAACTCAGTAAGATCATCTTTACTGGTTATCTTGTCAGGATTCTCTAAAGCCACCAATGTAAGCCAACCCTGAACCTTAGGATTCAAAATTGAATAGAATTTTAAGTAATCCAAGGTGGAGTTAGCACTACGCTCAGAAGTCATATTGATTTAATATCAAATTTCACAAGAGTGGAATCAGCCTCGAACACACAAAGTCCAGG

>Spalangia endius hurwu-like virus Segment 3

CACACACAAAGTCAGTACCAACATTTAGAATAAACCAAGCAGATTATTACTAGGAGATATCCGACTTGTTTGTATATCATTTATCCTACTCTCTGTTCACAAGTTATCCTAAGTGAGTGCGAATAACACATATTAGGCAAGATGATCGATTCAAACGTTATTAAGTACAAAGACTTTTTAGCTTTGATGAAATCAGAGTTGAATTCTTCCATAGATCAGCTAGAGATCAAGTTCATGGCATTTTCCTATCAAGGATTCTCTCCTCTTAAACTATTTAATGAGATGACTAAGAATCTAACTACTGATGAATTAAAGAGATCATTCATAAAAGATATGTTCATGCTTCTGGTTACTTATTTTGTAAGAGGACCAAATGTAGCAAAAATGATTGCTAGCAATACTTCAGGTGGTGACAGTGCTGCAAAAGAAATTCAAGATCTAGTAAAGAAGTACAAAATAGTGTCAAAGAAGCCAGGTCCTCTAGACGTGACCCTACCAAGATTGTCAATGATTTTTGCAACAATCTCAGAACAGATTTACACAAAATATGAAAGTAAAATTCCCACCAAGCCTGTAACTGAAGCTGAAGTAGGAATAGGCAAATCAATCCATCTAGCGCCTAATTATATCCCTTGTTTGATACCTGCTCCTTCATTGAACAGTAAGCATATCCATGCTTATTTATTTACCATTCATTGCATATACCAGAGCTTTTTATCTAAAAAAACAGACATGACTCCAGTTAAGAAAACCTCAGTAGTAGAATATTTTGACACATCCTCTAAATTTGCTATGATGGCATGTACTGGAACATTGTTGGACTCCACTACCTTGGTTGCAACTGCTAAGAAATTATCTGATGAAGTTACAGAAGAAATTCTGGAAGGTTACATTAAAGGAGTTCAAGCTTTCTTTTCAGTTCTACCAAAAAATACTATACATATTCATACCCATATCCATCATATTGTTAATTACTGCATTCTTAAGGGTAAGACTATGACTGAAATACCACATACTGCATTTAAATCTAACCTTGATACCATAGCAACTCTTCTGCCACTGAATTAGTTGATTCTTATAATATTAAAGGGCACTGTCCTGGCACTATCCATTAGTGCCGAAAAGCACAGCCTATTTCACCTAAAGGTGATTTTAAGGCTGTTTAGGTGGTGTTTTGCTC

>Fopius arisanus dicistrovirus

TTATCTGGTTTAGTAGCATAATGCGAACGACTGGAAACCACAAATATGATGACTTCTTACAAAACAAATGAATTTGAAAAATACGTGCAAGGTTGCCACTCCGTAACCATAACTATTTATGCCGACTTTTGCTTTTCCACGGAATATTCTTATTTGGACACTATTGATAGTTTGAAACAAGCTTTTCCGCTCGAAGAAAATGATATTTACTTCTCATTAGCTTTCAAAAAGACTAGGCGAGAATGCTTTAAAGTCTTGTCTCAATTATGTTGGGACGAAATTTTAATCGATAGTATAATTAAAATGCCATTCGAGATGCGAGGTATCTTCGATGAGTTAGAACCAACTGAGTATTCTTTTATTGAAAGATTACTAATGTTATCTGGTGATGTTGAACTCAATCCCGGTCCAGTGCACTCAAAGTCGACAATTCCAGAACGGGAATTTGAGGTGAACCCTAAATTTCTTCGTAAATTTTTGAAACTCAGAAAGTTGTTCTTAAGATTACAAAAGAACAATATTCCTAGTAGACAAATTCGACACAATTTGGACAAAATTAGTCAAATAATTGCCCAAGGTTCTACTGTGAGTCAGTGCTTAAGCGATAGAATCGATGAAGTAATATTTGAAAACAATAGAGTCGCTCAAATTGGAGTACCATTATCTCACTCTTTATCAAAAGACACTAAAGATTACTTAGATGATAAAATGCAGCAGGCTAGATCTATTTCAGATAAAACAATGGAAACACTAAAAGATCTGTTTACAACGTTCAAATCTAATCTGAATGACCAAGTTGGTTCGTTATTAGAGTCTATTAATTTAACACAGAAAATATTAATATTTTTAGCTTTTTCAATGTGCATCTTTTATTTAATTAGTAAATTTACTGGCCTCCCGGATATTACAAATACGACAGTTTTTGGTTTGCTTATTGCTTTCGTATCTATTACCGTAACGTGTCCCATTTTGAAGGAAACATTATCTAAATTAATAGAGAAGTTCAAATTTAAGAGAAATGGGAATGAACGATTTGCACAGGGCGACGATGATAGTTTTGATTTTTCGTGTTTGATACCCCCTTTCTTTTTAAATGCAATAGACGATGGCCCTTCAGCTACACTGTTGAATATGTGGAATTCCAGACACGTTGATCTAGCGTGCAAGCGAGTGAGTTATTTCGGTGACCATAAGCTTGAAGCTGGAATAAACCGCATTGGAACATGGCTGAAAGAGATCATAGAGGATACTATAAATGCTTTCAAAATTAAAGTTTTAGGTAGAGATCCTGCTGAGTTTGTGAAGGAGAAAAATCCTCTAGAGAAATGGCAAGAAGAATGCAGTAAGTATTTCGAAGCTGATATATCTAGACTAGTGACATATACTGATTCTACACTATCAGACTTGCAGCGCTTATATAAAACGGGCATGGATTATTTGAGACACCCTTTGTACAAAAGCGATGAGCGAATCATTAGGGATGCAATAAATCAAATAATGAGATTTGCGGAAAAAATTAAAAGTAAAGTAGGATCAACATCTTCCGTGAGAAATCCTCCTATAACTCTCTACCTATATGGAGAGACTGGAGTAGGAAAATCCACTTTAACTTACCCGTTGATGTGCCTTTTATTAAAGCGTATATTTGAAAAAGAAGGAAACAAAATTATGTTGGATGATTTGTCGAAGACATATAAGGAAATGATTTATGTTCGAGCTTCTGAACAAGAATATTGGGACAACTACAAAGGACAGTTAGTTACAGTGTTTGATGACTTTAATCAAATGAAAGATTCATCTAGCAATCCTAGTATAGAGTTATTTGAGATAATTCGATCGTCGAATATATTTCCTTATCCTTTACACATGGCGGCTATTGACGAAAAAGCCAACACGAATTTTAGTTCTAGAGTTATACTGTGTACTTCTAATAATAAAATACCGAAAACTGAGTCACTGAATTATCCCGTAGCTATGTTGAGGAGATATTGTAAATTCCTTGAAGTTAAGCGAACGCCAACAGAGGACGGAAAGTTTAGTTTAGATAGCTACACTTTTGAGGAATATGATCCATTAACTTCTCCTATTCAGGGTGCTCCTGTGAAATCTTTGACGCATAGTGAATTGATTGATGATCTTGTTAACAGATATTTTAATGACGGAAACTTTGTTAAGTCTGTAGATGATTTCATTCAAGGAACTATTTTCGCTCAAGATGGAGAAGATAAAAATACTTATATTTCATTAGGAGACGCTAGTTGTGGCATTAATTCAGCGATAAACACGGTATTAGAACAACAGGAGTGCGAAAAAACATTCTCTTATCGGTTTAGACAGTATATGGACGTGAAACGCAAAGATTTGACGGCAAAATTCTTGAAATATAAAGAAGACAATGACTATCATGTCTGGAACAGCTTTAGTAAAAAAGCAGCTATAGTTCTTGGAATAGTTGGTGTAATTTCTTTTGGCGTTGGTCTGTACAAATACTTTAAAGGCAATAAGCCCAGTATGAACCGAAGATCCTTTAGAAAGTCCCCACCGCCCGATTCCGATACTGAAGTTCCAATTGTAGAGGGATACAATCAGAAGGAAGTAAAGAAGCGAGTTGAAGGTTACAATCAAAAGGATGTTAAAAAGAGAGTAGAATCAGTAAAAATATCCAATGAGGAAGCTGCCATTCTGTTAAAGCAGGAAGGTTTGAACATTGAAAATCACGGTGAAGCTTGCTTAGACGTAAATGCGAAAGAACAGCTAAATAAAGTAGTAACTAAAAATTACTACCTTATGACTGTAGTTTGCGGAGCTAAAGAAATAGTTATTGGGCATTGTCTTTTTATAAAGGGAACAGTAGCTATAGCTCCTTTCCACTTCGCGAGAGTTTTTGATAAATGTTACAAGGAGAACAATAACTCAACTCTCAATTTTAGAAGTGCCTATGGTTCAGCAGGATTCACCACATTTTTGTCCGATATTACATTTTACGATTTCACGCCCAAAAATTTACACCCGATTAAATCTTACTCAAGAGATATTATGCATTTCTCAATCAGAAAAGCTCATGTTCATTCCGATATAACTAACTTCTTTGCTGATAGAGCAGAGCTGAGAGCTGTCGGCACAACACAAATAATACTGCCATTGATTAAGAAAGAGTCAAGTGGAAAGGTATATCTCTCAATTTCACAAACATCGGGATCGTCCTGTATTGCGAATGTACAAGATATTGATTACAGAGTAGGAACGGATTCTATGGAAACCATACTACTTAGAGAATCATGGGAGTATTCTCTAGATACAGTCGCGGGAGATTGTGGAGCTCCCCTATTTGTGCGAAATACAAAAGTAGGGCCGGGGCAAAATAATGGGCATCCATGTAGCTGGAGGTTATACAAAGTAGGAGTGAATGCCAACTATGCTACCCCTATCTATAAGGACGATGTTATAGAAATTATGAAACACCCTGATTACAAGAATACAGCGCAATGTTTCGGTCAAGTATTTAGGGAGAAAATAATTCCTATTGGAGTTCCCGAGTGTTATCAAGATTGTGAATTTTTGGTTCATGGCAAGGCGTCTAGAACCGTTCGTCATGCGACTAGAACTCAGGTTACTCCAACTCCTTTAAACGAAAAGATCAGAGAATGTACTATGAAACCGGCTTGGTTAAGGCCCAAATTGCTTGGAGATGAGGAATTCGATCCTTACATGTATCGGACTCTGAAATTCGGAAAGAAAGGAACACCAATAAAGCAACAACACGTCAGATTTGCTAGAGATGCACTAGTCAATGATTTATATAATAAATACTTACAAAATAGAAATCGATTGGAGGGTAAGTTTCCTGCTAAGTTGACATTTGAACAAGCTATTATTGGTATACCTGGTGAGGAGTTCGTGAATGCGATCAAGAGAGATACATCGTGCGGATATCCATTAACTCAAGAAAAATGGACAAGAGCAAAAATTTTCGGAAATGATCAAGAATACAATTTGGATACGCCTGGAGTTGAGATGCTTAGAGAAAGGGTTTCTCTTTATGAGAAAGGAGCTAGTGAGGGAGTGGTTTATGATCATTACTTCACTGACTGTTTAAAGGATGAAAAGAAACCAATTGAGAAAGCACATAAAGCCAGAATGTTCTCCAACGGACCAGTTGATTATCTTATCTGGTCCAAAATGTATTATAATCCAATCGTAGCTATACTCAGCGAGTTAAGAAACGATGATCATATCTCAGTAGGCACTAACGTTTATTCACAAGATTGGGATCATATTGCTAGATTGATGAAAGCTAAAAGCAATCACATTATCGCGGGTGATTTTGAGGGATTTGATTCCTCAGAGCAATCTGAAATATTGAACGAAGTGCTTGAAGTTCTAATTGAATTAGCGAAGAAAATTTTCAAAATTGACCTGGAACATGAAATTCAAATGCGTGCGATATTTGTTTCATTAGTAAATTCATTACATATTAACGGAGATCATGTAATCCAGTGGCTAAAATCACTACCCTCGGGACACTATTTAACAGCCATAGTGAATTCTATTTACGTCGCTCTTTTAATGTGTCTTGTGTTTATGGATTCCGTCAACAACTTTTCATACACTATTGCTTTATTGTTCTTCATGTTGTTTGCTCTCGTGGCATATGGCGATGATCATTTGGTGGCTGTGCCCGAGAGATTTCTCGAGCACTTCAACCAGATTACTTTACCTAAAATCATGGAGCAATACGGTATGTCGTATACGTTGGAATCGAAAGAAACTGACGTAAATTTCAAATCTAGAACAATAGAACAAGTAACATATTTAAAGAGAAGGTTTCTTTTTGATGAGGCTAGACAGATTTATATAGCGCCTCTAGATTTAAACGTTGTCTTGGAAATGTCTATGTGGACGAAATCAACAAAAGACATAGCTTTAAACACTCGCGCAATATTGGACAAAGCTCTATTGGAACTGTCTCTTCATGAAGAAGAAACCTGGAATGAATGGAAGTCGAAGTTGATCGAGGCAGGGGGAAGAGCATATCTCCTATTCATCACCTTATATCCATTTCGACGACACTCGAGAGGAAGCTCTTGGGTGTAGTATGCAAGATGATTTTATTGCTCAATCATCACAACCATCAGATGTGATCTTTGGAAATACAGACAAATTACTCCGGTCTCAATGTAACTCCAACTGCTATCTGATGCAGGCTCGTTCCTATTTAGGATTACCCGCCAAGATCGAGCGAAAGCAGCCCTTTCAATATCTAGGCAAACCGGAGTCGGTTGACTCGTTTAAGTGGACGAACAACTCAAAAAACTCACTTGCTGAAACAAAAGACATAAAATATCAAGAAAACCCCCAAACTGACGATGTGGAAGAAATCACCACATTTGAAAATGACTTGAACGTAATTGAAGAAGGCCCTTCTCGCGAAAAACCTTTAGATTTCATGTTTAGGTCGCAGCACACTGACGAACGATCCCATACAGTTGTAGACTTTCTCTCTCGACCTCAAGTTATTTTGGAAACGACTTGGGATGCCTCTAAGAAGAGAGATCATACGCTGTTTGAATTAACAATTCCGGACACTATCGTTAAAAATGAAATGTTCATGGAAAAGTTGAAAGGATTTTCTTCTTTAAGAGCTACAGCTGTTATAAAGGTTCAGTTCCAAACTCAACCTTTCCAAGCAGGAAGGTCATTGATGTATTCTCTACCTTTACCTACTTTGAACATTGACAGAATTGGCTTTACTACTAACAACACCAGTAGAGCTATGCTTTTAAATCATGTTCAGTGTGATATTGCAAAACAAACAGAAATATCTTTAAGAGTACCATTCATTTCACCTTACAATTCTTATGATTTAATATCTGGAAAATTTTCCTGGTCCAAGGTGGGAATGCTTGTGTATTCCCCCCTGAATACCGTAGGAGATAGCACCGTGGACGTAGTAGTCTACGCACACTTCGAGAATATTCAGCTAGGGTGCCCAACATCTGGCATATTAGCTCAAGGAATATTTGCTCAAGCTGGAAAGGAAATGCCATCTTTTTCTGACTCTCGCCAGCGATCTCAAGAGACTCAGAACAAGGATCATTCGAAGACCGGAGGTGCAGTAAAATCATCATTAAGGTCTTTGGGTGAACATATTCCTTTTTTGGCTAAAGGCGCTTCGTTTGTTACTGACAAAATAATACAGCCACTAGACGATATTTTAGGACCTATATTGTCATTATTCGGATTTTCCAAACCGATATTGTCATTACAGAATCCTCTTTCTCTCAGGCCAGCAACTACATTTGCTACAACTGACGGAAATGATATGTCTCAGTCGCTTGCATTGAGTGATAGTACAAACGTTCCTTTTATTAAATCATTGGACGGTACCGAAAATGATGAAATGAGTTTTGATTATCTTAAAAAGATTCCACAATTCATAACATCTTTTAAATACTCGAATAGCACTACGAAGGGTGACGTTTTGTATTCCACATCAATTAGGCCACATTATTGGTTACAAGAAGGCTCATTAAAAATCAGAGGAACCTCCGGAACAACAACTACCATTGTTACCGTTCCACAACCTAATAATTTGGCGTACTTGACTGGATTATTTAAATATTGGACAGGATCATTAGTTTACACGTTTAAATTCGTAAAAACTGATTATCATTCCGGTAGGGTCGAATTTTCATTCCACCCGTTTTCTGACTATGTGAAAAATAAGACTATTTCTGATTATTGTTATCGTATGATAATTGATTTAAGGGAAAAGTCTGAAGTTTCGTTTCTTGTACCGTTTGTATCTCCTGTTCCCTTCAAAAGAATAGGAAATCCAGACGAAAAAGATTATAGTAAGATGGCTCATTCCGCCACTGGCACGCTAGTGGTTAGAGCATTAACCGGACTTAAGTCCTCTAGCGCGGTTGTTTCCGGAACGATAGAAGTTCTTGTGGAAATAAACGCGGGAGAGGACTTTAAAGTATCTTGTCCAATAGAGAGCATGTACAAGCCGTTTACCATGACAAAATCATCATTGTCTTCGCATTCTGGTATAGACTCGCAAACGTTATTTTACAATCCAAAAGATAGTAAGAGAACGCCTTATACACTTGAAGAATTGTCTCTTTTAGATAGCGACACACAGGCTGAAGTTATAAACAGATTAGAAACTCCAGTTGCCCAGTGCGGCGAAGTTTTCTCCTATTTAAAGAAAGAAGACAATCTTTTATGTATGTGCTCTTCGACACCCCCATCATTAACGCGAGTTGCGCAGTCTTTCAGTGGAGCGCAACAAGAAAATCCACAGAGTAAATCGACAACTCAAGACCCAAAGTCGATAACTGTCTCTGGCAACCCATATAACCCGGACTTATCATTAGTAATACACGGTGAGATGTTTAGTAATTTTCGTAACTTTATCAAGCGAGCGAATTTTGGTTTGTACAGAGATACATCAAGGTCAAATAGTGTAGTTGACACGATTAATCCTGAAGAGTACATTAGACCACCGACCCTCTATCTAAATACATATAGAGATGGTTCAACACCACCTAAATATCAATTTGGCTTCTATATTTACAGAGCAGCTCCTACTCCTTTGAGTTACGTAGCTGCTCAATATACGTTGTATAGAGGGGGACTTCGCGTGAAAGTTTATTCTGACAGTGAACATAAAACTGTTTCCGCCAGATATAGACCAGCTGAGAGTTACGGGTATGGAAAGAATTCCAATTCCTCCCCCTCACCATTAGCGATAGAACAATCTCCGATAAAGGGAGTCGCCGAATTTCAAGTACCGTATTACTCACCATGTATGGTGTCTTCATTTAAGAAAAATGAAGAAAGGTTTTACTATAGTACAGGAAGGAATCTTGTCGATGTTTCAATGTCACCTAAGACGAATGAAAGGTATTATGCTTTTTCTGCAGCAGATGACATGGACTTTTCCATCTATATCGGTACACCACCTGTAATTCCGAGTTTTTACACCACATCGTTGGAAACGATTGGTGGAAAGACTTATGCGAAACTATTAATGGACAATTATGATCCATATATAGTTCCGGTTACCAGTGATGCAGTCCCATTGAGTAGCACTCTTGTTACTCTGGAACGATTGGCGTGATCCCCTTAGAGGCAGGGTCACCTTGAATGTCGCTATTAGAATATATTAATAACATTCCAGTAAGAGTTTCTTCACAGTTCTTCTTTTACTGCGTAATCGAACTGAAGTATATTTTAATGTTTCTTATTGCAAAAAAAAAAAAAAAAAAAAAAAAAAAAAAAAAAAAA

>Fopius arisanus Nora virus

ACAGAAAGTAAGTGGTCGGAGTTGATGAGGACATCTCTTCTCCCGTTTACGTTCCCAAATTTATGGTAAACCCAAGCCAACCATCAGGTGATATAGTAAAATCACCTTTAACAAAATTTTACTCTTTAGCAGATACTGTAGATTACTACCCGTATATAATATAGGCTCTAATCTTTTTATTTTGCTATCGTATGGTCAAACGAATTGTCACACGAATTTATCAGTCGCCATGATTTTAGTTTTAAGTCTTTCGGATCAAATAGGTATATTATCTATAACGCGATCTAAGAGTCCTATTTCGAATATTAATTTCTTAATAGTGTCTAATCTCTAGCAGAGATTACAACTCAGTGAAAAAGAGTATAAATTTCACAACATAAATAATTATGAATTTAAGCAAAAACAAAGCCTCGGATAAGATCGGATTTAACGTTGACGAACCAATTTGTTTACAGACAGAATCACGGATTGATAACACAAAAGAGTTTTCATTCTTTGATTTTGCTCTCGGTATAAACAAATCGCCTGTACCCCTGAATTTCTCTTTTAATAAACGAACGGAACAGACGCCGAAAGTTAGAAGTACTAAACGCGGTTCTGGAAGAAGGGATTTATTCCTTTTCTGCAAAGAACTAGTTAAAAGTAATTCTCTTTCACAGGAGTGGGTTGTAGATAAAACTCCACAACCCGAAGAAGATCTCCTTCTCGATCAAAGTGATGACGAAGAAGATTTTCCTATGGTAAATCTTAGAGATTACCTAAAACCTTGTGTTTTACCTACCAAAATAAAGAAAAAGAAGGTAGGTTTCACAAGAGCCAATGTGGATTTTCCATATTGTGTAACAAGTGTTACTCAAGTAACACCTCATCCTCAGGATGAACAAAAAGTGAGTGCTCCACTTAAACTAGCACAGCAGCATAAGTCTGTAACTAAAAAGCAAACAATGGCGAGTTCTACTAAATCAAGTGTACCCGCTTCTGGATTAGATTGCGAATTAGCATCTACTCCTGCTTTCAGCATTCCAATGCTAACCCCCTCGGATTACAATAACAACATCACCATATTGAAATCCGTTAATATTGAAGCTGATTTAGCTTCAGTTGCACATAAATTTGTGAGTGTTAATGTTTTACAACATGAAGAATATGCTTTCAAGCAATTCTGTGTTAATAATCATTTAACAATTTTAAATTCATGTGCACTTAGGGTATTCGTGCCTACAGTTATTGACAAAATTCAATTTCTGGTGACAAGAAAAGGTCGAGCACCAAGCCCACCTAAAGCAGTTGAACATAACGAAAAGCCAGCTGCCATTGAGAAGATTCAAGTGAAAACCGCTTCTCAACAGGCTTCCGATTTCAAGCCAAAATCTAAAAGGAAATCAAACCGTAAAGGTCCTACTGTGAGTCACGTATCACAGAAAGCGCAATTAGCGCTACGCGGAGAAAATGGTCCCCGTACTGCTCAAAACAAGAAAGTAGAAAACTCATCACCTTATAAAGGTAAGATTGACCATTCAATACTTCAAGAGTGGGATAAATTATCCCCTGAACAAGTATTGATTGCCCGCGAGACACGCTTGCGAGAAAAACAAAACCATCGGGAAGAAGTTTTAGCAAGTGTCGTTAAGGACAACAAATCCTTTGCGGATTGTCTTGAAGGTGTTAATAAAGTTATGACAACTTTTAGTTACCTTCAGATAAAACGTGAAGAAGCAGCTAAAAAGCTGAAAGGCATTCCCCATAAGGCATGGACTGTCATTAGAAATAACCAAGATAAAATAAGAGCATATTACAAACAGCTCTTCCGCTCATCATTTTATACTTTGAGAGCGCGCTGGATTAAAATCCAAGTAAAAGGTGAGGACATGAATCCTTACATAACCGATTTTGGTTATTTCTGGCGACAGATTAATGTCGATGATGTATCAAAGATTTTTGATAACATCAAGACTTGGGAACAGAAAGTGATTGCCTTCTACCCAAAGTCTTCCGCTGCCAGCGAATGGTTCAAACAACCTAAATATGATTAAGCTCCCAAATTTCATATATAATACCAGATTCAACATATTAGCTTTTCTATTATGTTGGCTCTATCTCTCCTCTGCAGAGCTGAGACAGAAATGGAATCTTTGGATTAAGTTTGCACAAACTATTGATCCATATTTAGGGACACCTTTGAGTGGACCTTTCACAACCTTAATGTTAAGTATAGTTGTAAGCTATACAGCAGTATGGTTGATATCCAAAACACTACTCTACGCAGTAGATAAGGTAACTGAAGTTATTCACGATTTTCGTGAAAAGTTAACTACTCGAGTTATAACAGGAAATACTCGATCAACCCGCTCATTGCTCCGTCGTCTATATGATAGTAAGGTCGGAGTCGCAGGAATCCTGTACACACTTTCACAAGTACGAACAACAAGTGTGATTATAGCTCAAATGACTGTGTTGAGTAGTCTTTTGGATATAAAAGAAATGGTCTGGTCAAGTCTCGTCAACAATTTAGTCAATCGTGGACGAGATATAACCATGCCAGCTGGTGCTGAAGAAGCAACCGAACATGGGATAGAAGAAATTATTCCTTCAGCAGCATTGTTTGCTACAGTGACGAATTTTGAAGTTGGAGATGTTAAAATCGAAAACTTTATGAGTCGCTTAGCATCCAATCAGCGAAATGCTGACACCATCTGGAAGAGCTTAAAACCAACGCTCATCAGCTTAGGCTTACTTAAAAATTCGCAGTATGAAGCAGTTATTGAAATTTCTAATACTGTGGCTACTTTAAGCGAAGAAGAAGCCTGGTTGAAATTCAACTTGAGTCATTCTCCAGTTACTTTGCTCTCTGGTAATAGCCAAGTACGCATTAAGAAGTTGGAAAAAGAAGTGCCACAACTTCTAAAACGTCTAACTACTGTTGCTACTAAAGAGTTGCAAAATGACAAAGGTGTGATGAACTGCCTAGCTCGACTCAAAAGTATAGAAGAACTTCTCATTAGAGTTCAATCTATTCAGAATTCATGTGAATTCAGAGTTAAGCCAGTTGGTTTATGCATCCAAGGCGAGAAGCAAGTTGGAAAGACTTCTATGGTCCAACTACTACGAGAATTGATTGCCGATGAATTAATCGAGTCAGGTAATCCAGCTTTTGTAAATGCCCGTAGTTGGGGTATATGGAGTCGTCAATGTCGCGATGATTTCGATACTAATTATCGAGGTCAAGAAATTACCTACAGCGACGATGCCTTCCAGCAAAAGGATAATGCTGATCACTTGCTATGGTACAGCTTTATAAGCGGTACAGCAGTTGGCACAAACCAAGCTGATCTCCACAATAAGGGAATGCCTTATACGTCGAAATTAGTTATAACAACATGTAACAACTTGCCGACGACGAGCATAACTGTTAATGACATCACAGCTTTACATGCTCGATTTCCGTTTACTGTACGAGCTTATCGTAATAATCGATCAATGCCATCGTTTGGTTTGTTTGACAAATCTTACAATTGGTTAGATCTTTACGTGGGCCCAATGGAAAAAATGATTGATTTCGAACCTCCAATGCATACACGGATTCAAGGGTTGTTACCAGCAGGTATAACTAAAGTCACAGTTAAAGAACTCGCACAAATGGTTGCGAAGGCACTTATTGCCGAAGAAAAGTTCTTTAACAGTCGCTTACCACCTCAACCAGCACCAGCGGCAGAGCATATTGAGACAATCGATAAGGATGTTAAACCATCAAGTTTAATCTTTAGCAATCAGAGATATAGTGATTTTCTCGGAATGCAACAAGATCGTGTGTTATCTAACGGAATGATTTCGATTAACATTCCACGTAATGAAATCTTTTCAGGACTATACCATGGTTTTGAAAGCAAATCTAGTTGGTTAGAGATCCTCAGGAGAGATCTTCCGGAAACACGTGCTGTACGTGAAAACAAAGACATGATGCAAATGATAGGAGATTATGTTGAACGTAACTCTGATAGCTTTTTAAGCTTTTTAAATTTGCAAGTGTTGAAACGTAGTAATGGTGATCTTGAATTATTCAAGAAGAAGTTAATACGTTCCCTAACAGCGGTCCAGTCCTTATCTGGTGATAATGAGACTGAAAACACGGAAGATATTCCAGAGTCTTTAATAGACTACATGATTGCAAAAATTTATCTTGAGCAATGTACGGCCATTCAAAGTGCACGGTGGGATTCAGTCTCAAATTGGGTTAGTTGTATTAGAAGAGTGGATTCAAAGCAACCATTCTTTCAACATTTTCAAGCTAACTACCAGTCTGTATCTGAATTCATCACAAACCTTGATGACTGGGAAATAATTGAAGGCAAAGAAGAAGAATTTCGTATGAAATACAGTCATCAACTGCCGATTGCTCTTGATAACAACATCTGGTCTCCCCTTTTCAAGGGTGGTAAAGTGGTTATCCCTGCCATGTATATCAAACGAGGTGCCACAACACTAACTGGAGCGACAACATCAACTGGTGAAGTTAAATGTGAAATTCTCCTCCAGGGAGGATTACAAGTTGATCAAATCATTCATGACCGTTTAATACATAACATCAATAATGGACATGAACTGAATGACTCAGCTTTATTCCTCCCTCCATTGGAAGTTTTAGTGAGGGCTATCCAACCAATGAAGCAACCAATAGTTTATCCAACTATTACTTCATTAGCTCAAAAAGAGCAAGCTGTCAAGAAAAACTCTATCAACATGACAGTGTTTAAGAAAGAAGTTGAAAAGAAATACGAAAATATAAAAGCTTTTAATAAAGCGTTATCAACTTCTGGAATGGGTAAATTTATATCATTACTTGATAGTTTAGGTTTACCTGTTAACGAATACTGGCGTGAAACTTTCATGGAGAATGCTGACATCATCGTCCCAGCAGCCATCACATTGGCAACATGTGCTATCATATTTGGCATTGTTAGATTGTTACAATCTACACAAGCCACTGAACATAGTGCCAATGAAAAACAGCCTGGAAAGAAACAGCTAAAGCGTAAGGAAGTCATGCGCTTAACACGCAAACTTCCAAAGGCCGCAGAACATAATGATCTGAGAAAACAAAAAGTTTTTAACACACCAAAAATTGATGTGGATGCTTTTATGGAGCAACTCGAAGAAGCTCTAGAGGAACAATCAAAAGCAACCGTGTTGGTACTTAATGTTGATGGTGTTCAAGCTTCAGGTCATTATGAAGCTGAAGGATATCTCAAATACGAAAATAATCGTATTGTTAAAGAATTGCATGTATCACGCGAACGTGAACAAGGACTTTATTATATTAAAGTTGTTGTCGCAGATGAATGCAAATTCGATGATTTGGAACATAAAATCGCAGATATCCTGAAAACATGCGAAATGTATCCAACGGCCGACTCCACATTGGAGATCGAAGTTGAAAAAATCGGAGAAGATCAAGTCTACTTCTACCTGGAATACTACTCACTAAACGCAAGATTGAATGGGAGTATTAGGCCATGGACGAGGCGAGAACTTGCTAATCTCCAGAATCTTCAAACCGCTTTAGCTAAAGGCGAAGTCCAAGATTTGGAAGCAATTGTCCTGGGAGGAGTTGAACATGGTGAAAAGATCAACCAGGAAATAGCAACAGCATCAAGTCTGATCAAGCACAATTTAGTGCAAATCGAAGTAGCTAATGCAGATCAGTGGGAAATTCCTTCAGGTTTACGAACACTAGGTCTAGCTCATCGCAATTTGATAATTTTCCCTGCACATATCGCAAAGCATGGAAAATATTTCAAATTTTATCTAGCAAAATCAACTAATAATAGAAATTATTATGTTGCTAGAAAAACTCTCGAAGATATCGAGCGCGATGTAGCAATTGCAGTTATTGTTAGCTACGACGAGGTCATTGCTATGCTAGGTCCTCGAAGGAATCACAATGTTCCTACATTCATGTCGAGAGAAAAGCGCACTTTTCCATCTATTGACAATCTGTTGCTAGACAGAGAGACAATGAGTGATGCTTTCCTCGGTCATGATAGTATGCACTACTTCCACAAAGGTAATTGCTTCATGTTTGGACGAACTACCGGATATGACAAAGTCACATATAAGATCAATGAATCTTATATTGACAAACAACTGATCAGTACAACACCAAAACTAATCGCACATCTCTCACACGCGGTGGCAGGTGATTGTGGAGGTCCAACTTTCCTCGTCAGTGGTCCAAAACGCGGAGCACTATTAGGTTTTTATGTCCAACAGACAGATACTAATTGGTTTTCCGCATATTTGGTCAAAGAAGATCTTGCTGAAAATGCTGTAGTTCAGAGCTACGAAGATCCTTGGAGAAAATTAATAATAAACGCACCTCCAGAAGATCTACCCAATGGTCCAGAGCTAAAATATGTAGGTCGACTTGTTCGTCCTACATTACCAGCGTCAAGCGTGAGTTTAGATCATTGGAAAAAGAGCCCTTTCAGCAAAGTCTTTAAAGAACAACTGGCTCCCGGTCGATTAGATCCGAATGATCCATTCATAGAAATGGAAGTTCCTACGAATCAAATTGGGCGAAAATCATTATTGATGAAACCTAATTCTCTTATGGGATTAACTCTTCCAGAATTGGATCAACAAGTGTTAGACATTTGCGTAAGCAAAATAACAGAAGAAATGACTCTAACATTTGAAGCAAATGGTTTTCTAAAACCAGTGGCGAAGGATATCCCGAGTATGCTTCAGTATGCTCTTAACGGATCACCCGATAACAAATTTGTTCGCGGGATGGAAGTTAAGAAAGCAGCTGGAGTACCTTGGAGTTTAATCGGGTGCCAACGTAAATGTGACATGATCGACATTGACGAGCAAGGAGTAAGATCGTTTAAGAAAAATCCATACGGTCAAGCACTTCTTAATAGAGTAACTGAAAAGTTGAATCAAGCTAAGTTGGGCAATAGAATCTTGAGTTTTAGTAATTCTAAACTTAAAGATCAATGTATCAAAATTGCTCAAGCAAAAGCAGGCAGAACACGTGTATTTCAATGTGTTCCCGTTGATTCAATCCTCTTTTCAGCGGCTTTATTTGGACCTTTCAAAGAGGCCTTCACTCGTAGCGGACTGAAATGCTACCATGCTGTGGGAATTAATCCAAAAAGCAGAGACTGGGAATATCTAGTCAATTATCTGTGTCAACATCAGAATTTCTTTGATGCTGATTATTCTAATTACGACAAGTATTTACATCGTCAAATACTTCTTGCAGTTAGAAAAATCCAACGAAAAGTTATTCAGAATTTGGCACCTGATGACTGGGATATTGCTCGACAAGTTGAAGAACTTGACACAACCGACACTCTTATTGTCGATTATGCCACTGTTTACCAAACGAATAGAGCTAACAAGAGCGGTAGCTATATGACTACAATTGATAATTGTATAGCTAACGATCTTTACGGTTTCTACGCTTGGATCAAAATCACTGGCAACGATAGCTTAACTGAGTATCGAGAAAATGTTTGCACAGTTTCCTTTGGTGATGACATCATCAAAAGTGTTTCCGATAAATATAAGGATAAGTACAATTACTTAACTTATAAAGTTGAATTGGAAAAATTGGGGCATATTATCACCCCTGGTGCAAAAGATGGAGTCGAACGAGCGATAACATCGCTAGATCAACTCCAATTCTTGAAACGAGGTTTCGCTCAGCACGATGGATACTGGATTGCTCCTCTATTAAAAAGATCAATAGAGGGACCTTTTCATTGGACTACTATTGATAATAGTAGTTATGCTGAATGGAAAAACTTGTGTCAGGAACAGCTTATTGAAGCAGCTTTACACGGCAAGGAATATTACGAACAATTCCGTTATGGTTTGAAGCAGTGTGAAGACAAGGGTCTCTTAGAGGCTATCAAGGGAGTGTTACTCGATACGTGGGAAAATACCCTTGAGAAACTATTTACCGAGCGCTATGTCTAGAGATATCAAAGACATAATATTTAATGAGAACTCAACGTTATTCCAAGTTCTCGATCAGTTGGATATAACTGATATAATAGATATTAAAGCGCAGACAAATAGTCTCCAACGGGAGGTATCGACAAACAAAATCCAACTTGACAGCCTCGCTATCAAACAGGACAACTTTGAAGGAACCGCCAATGCACATTTTGTAGCAATAAACACTCAGTTCCAAAACCAATCGGAACAACTCGCAGATATAGAATCGAATATAGTCGTTGTTAAAGACGACATAGGTATTCTAAGCGAGAGAGTTAAAGATCATCAAAATCAAATTGATGATTTTTATGCAAGTTTTGAACTTTTGGATCAACAATTGTCTAACATCGAACAATCTATTACAGATTTAGGTGATTCATTTGAGGGTCTAGAGATACGACAAAACGAAGTAGACATAAAAGTTAATACCATGTCTACTAAAGTTGAATCTTTAAAAGTTTCTATTGATGCTACAAATAAAAGAGTATCTGAACTTAATTCAAAAGTTTCAACTATGCAAAGCGACATCGTTACTATACAAAAGCAAATTGTAACAGGAGTTCGGGTATTGCAAGGAACTAAATTGTATGTTTGGCAATATCAGTTTGGTCGAATTTCCGAATTCACATACGCTGGCAGCGATGCTTGGATGTTGCTTGGAAAAACCTATGCAGTCAAAATGACTGGTTGGTTAAATGGCGACTTGAAGATCGAAGTTGGTCCTAACGACCCAGATGTAAACAATAAGTACATCATTCCCAGCAGCCACTCTGTCCCAGTGGTATCAGGAAATATTTACCGGTTCTATTATGGATCTGGTAATCTCAATTTCACTGCAATGATTTATACATAATGGGTGGCCCACAAGAGATGACAATTAGGGCTTATGATAGACCAATGCGTATATTAGCTGGTTTATCAACATTATCAAAAACTCCCGCAGAAAGCGATGACATCGACCTTGGCTTGGTCGTCTCAGAGGTAGGTAATCCAACAACTCTGACACATCCAGCATGGCCTGATCCCTTTATAGGGTATCACCTCCGTGCTCCACGTGAAAATTGCGTGCCGGATTTTATTTTCGGATCAATGGAATTAGGAGACGCATTCTCAGCGTTTCTTCCACGAAAATTTCCGGCTCCAGCAGTAGGTACACCTTTGGTTATAGATCCTATTATAACACCAGCTCAAAAAGTGTTCTTCGATCTATACAAAAACATATCTGCTGATGTGATCATGATTATTCATGTGCCAGCTCCACTTGGCGTAGGTATATACCTAGAGGTTTATGCTCCTGAGTTTGATATAACAACTGTCACTCGAGGTATAAGGTTCAAACCTTCAGGTCAACCTACCGTAGCTGTAAAGCTACCATGGAGTAATGATTTGAGTCTTGTGAATTCTCCAAGACCAGGTCAAAGCGGTGGATCTATCGTTATAAAGACAGTCGAAGATAATAGTTCTGAAACTGTCAATACACCGATAGCTATCACCGTGTGGTGTTGCGTGACCAACGTTAATCTCACTGGTTGGGCACTTAACGCCACTACCGGAATTGACTATCCCAAAATCAACTTTCAACGCCTTACAGCGGAAGATGATGAAATATCATTAGATGAGTATATAGATGATTCTCAATCTGAAACCAATTCATCTACTGGTAGTTTCATACAGGAGGATAATAGTTTAACTATCAATCCGAAAACTGGTGAAATTTACCCCGTTGCCTTCGAACATGGTGAAGAAGATGATAACGAAGTATCTGCTGAAGGAGGCAAATCGGTTGAAGACTTAGAATATTCGAAAGTCGGAGTACCTGTTGCCCCTCTTGTAGAGACTCAGGCGGAGAAAGTTGACCTCCCCCTTCCAGGAATGGCCGTTGAGAAGAAAGATACCGGCCATGTTGCTACTAAGTGGTATGCTTTTGCAACCATTGAAATTAGCGACACTGTTACAAGTTGGCAACGTGTAGTGATCAATCCCTACACAAATGCTAACTTACAACAGCTAGGAGAATCACTTTCTCTAGCCTGGAAACGCAATGTCTGGACGACAGGAGCGATGGATATTGGATATATGAGATCTCTCGTAGTCCAAATCAATATCCCTCGGCCTCCTCAAATTTCCGGTGTATTGGAAGTCAAAGACAGTGCGAACTCATCTTCCATCCATTTGATTGAATTTGGAGGAAAAGTTGAGATTCCGCTCATTCCGCAAAATTGGAATGGGTTGGCAACATCTTTACCGAGGTATTGGTTGAATCCTTGGTTAAGAACTGATGAGAGCGAGGTAGCTTTTTACTACCGAGTGGCTGCGTTCAACCGCACATCAGAAATAGCAAATCTGAACATCCGCGTACTTGTACGTCCAGGTTATGCTAATTTCGAGGTTCCATTGAAACCTCGTCCCCGAGCAGCAGGGTCATTAACTGAACTAGCTCAATTAATGCGACAAGCAGTTGTTGCATATGAGCATGGTTCGTTACCCGAGCAATCCAGCTCATATGTGGCTAGTCCACATCAATTCATCACACCTCAGCAAGCACATTCAGCTGAGCAGTTCAATCTCCATCACCAACTTGGAGATGATGAAAACATTGAACTTGATGAATTTCCAGTTCTCGTATATAAAGGAGAACTAAAAGTGGGAGAAGTTACTCCCATTCCACTTAATCTAGCTGAGATCGTAGATCTTGATTGGGAGAGTGGAGAAAATGCAATATCGCAAAAGTTTGAGCGATTTGCACATATTATCCCGAAGAGCGCGGGAGGTTTTGGTCCAGTAGTAGGTAATTACACTATTGGATTAAGACTTCCAACCACCATTGCTGGTCAAATAGTTCACAACTGTTTACCAGGTGATATGGTAGATGAAACAGTAGCGCGTATTTTCGGGTTATCATCATTGCTTGGCATAGCTGGTACAGCTATTTCAAGCGTTGGTGGACCGTTGGTGAACGGTATAATAAACACCACAGCACCGATATTAAGCGGTGCTGCCCATGCAATTGGCGGTGACGTCATTGGCGGAGTAGCAGATGCAGCTCTTGGAGTTGCGAGTTCCATCTTCGGAAAATCGAAGAAACCTAACGTGAGTGAGGAACCAAATGCTATCGCCGGTGACATACCGATATCGCGATTTGTCGAGATGATCAAGTATGTTGGAAGCAACTACTCTGAGAATCCAGTGTTTCCAACACTTTTGGTCGAACCACGCAACTTTTTCGACCAAATCGGCAATGCGCTCAAATCGATTCCAGTCGAGATTTTCGCTAATATGCGAAATGCAAAAGTGGAGAGAAGTCTGTTCGACAGATCAGTACTTCCAGAGCCTACTATCGAGCAAGATATTATTATCCCTCGAGAGGCCATCCCCCGAATCCTGGAAAGATTTGATACTCATCGCGAAACATGGGTGGATGGAAGCAAACAGAATGTCTGGCTTAAGAAATTTATGCTAGAACTGCGAGAACGTAAAGTTGGTTCTCTTAGCCTGTTAAAAATCAAAGGTCACTCTATACCTTCTGGTTTATCATTGCTTCATCTTATAGAGAAATCGAAATCCCTATACTTAGTGTAGGGCGGAGAGTCGTAGTAGTTTTACGCCTGACCTCTCTACCGGAAATGTCCGCGAAGCAGTTAGCGGAATAGTCCGGCACGTCAGGGTTGAAACCTTTTTCCTACTACGTATCAGTTACGAACAACACCTCAGTAACTTGTTTTAGAATTAAGCACTTTAGTATTTAAGAAATGAGCTATTCGCTATAAACGCGACTCATAAAAACAATATAAAAATTATAAAACAATAAAAATTACAAAAACATTTTAGATTTAAGAAATGAGCTATTCGCTATAAACGCGACTCATAAAACAACATA

>Zeugodacus cucurbitae dicistrovirus isolated from Fopius arisanus

GAAAGGATTACCTAAAACAAATTTTTGATAACCAACTTCACTACTGTTTGATTGACGCAGATTTCAACCGGAACATGTTAGTAGGCTTAGAATATACTGATATGTGGTTGGTACAATTTTATAGGAGCTTTTTGCGACCTTTATTTGCGTCAACCTATTTATCAGAAACAACTATAAGTACATATGTTCCGGTCACAAAAAGATGGATGAAATTAATGCAAAAATATAATTGGAAACGAGTGGTGGAGCAAATTGCGTATAGTGAGTTCTATCACGAAGAGATTGATTGTATTGATGGTGATGCATTGGATAGTCGACACCTAATGAAATGTGGTGATGTAGAATCTAACCCAGGCCCTGCTATTTTGAGCACTCTTGCCCGAATTAACGACCCGAAGGTCAAGAGGGCTGAAGTACAAGGCTGGTTAGATATCCCTCACTATTTGGGAAAAATTACGTCTTTCCTTTCTGAACAATTGCCTGTGTTAATGGATAAGTTCTTTTTTACTGTTGAAGAAACTCGGCAAGAATTTCATGAGGATGTTGAAACTATTAATAACACGACAATGAGTTTGACTGACAAATTAAGTAATCTTAAATATGATTTAGTTAAATGTTTCATTCTCGTATTGACAGTATTCTTATTGTGTTTATATGAACAGTACACTCTTTCGTTAATTTCTGCTATATTAGGTGTTATTGGCTTGTTTTCCGATTTACCACGTGAGTTAGTTCGTTGGTTAAGAGATTACTTTGCTCCAATCCAAGAAGAAACAACTGCCGAAGTGCAATTAGGATCAGGTGAAGCAATAGCCTTCTGTGCACCAGCTATTATGAGTATTTTGGCTTTTTATAGTATTAGACGATTACCTGATAATAGTGATATAGAAAAGTTCTCGAAAAAGGTATTTAATATTGACAAAGGGATAACTGGATCATTACATATGTTTGACCACTTTGCACGCTTATGGTCTCATTGCCAAGATTGGATAAATGAGAAAATAGGCTTCACTGAGGAAGGTAAAATCACTACATTTGAACAAGACGTAATGAAATGGTACAAAGACATTGAATATTGGAGTATTTTGAAAAACAAGGAAGATGCAAAAAAAGATCCCAATAAAGCATTGACAATTTCCAAATTATATATAACTGGAATGGGCTTAAAGCGTCAAGCTACATTATTAAGTATGGACAGACGTATCCACGATATAATAAATAATGGGTTGAATGCTGCCAGTAAGTTATACAAATTCGTAGAAGAATCAAACGCTATTGGTGGAGGTACACGCTTCAAACCGTTAGGCATTGCTTTATTTGGTGAAAGCCAAATAGGTAAAACTACAATGGTGGAAGCGTTATCTCAAGACTTGTTATACGAAATGGGCCATAGAGATGTTGAGACATACCGCAATGAAATTTATTCGCGCCAAGCAGAAACAGAATTCTTTGATGGTTATGTGAATCAATCTATAATTATTGTGGATGACGCTTTTGCTGTAAATGATAGTCAGAGCAAACCAAATGCTGAAATTGCTGAAACCATTAGGATGATCAATGAATTTCCACATCACTTGCATATGGCTATATTACAGGACAAGAACACATACAATACTTCTAGAGTGGTATTATGGACAATCAATAATATAAATATACAGACCCCATCTCTATCATATCCTAAAGCATTTCATAATCGGTTAATGGAAAATGCTTATAAAGTAGAACCCGCTGACCATGTAGTGAAAAAGGTAAAGGATGGAAATGGTAGGGAACAAATGTTGTTGGATCAAAGTAAGATTAACACAAAAGATGGACCAATTGATCTGAGTATTTATAAGATAACGAAATATACCCGAATTGAAATTGATGGCGGTGTTGTTTATAAACCTGTGGGAGATCCAATGGATTACGAGACTTTTGCTCTTCATTGTAGAGACTTGTATCACCATAGAGTAGAACGACACGCAAATAAGACTCAATTCTTGAATTCACGATTTGAACAACTGATTAAGAATCCTAGGAAAATTGAAACTCAAATGGGAATTTTTGATGATCATAATTTCCGCAGCTTGCAAAACGAACCCACTCCATGTTCTGCTAGGCAATATAGGAGGTTCCCTACTAGTCCTCTTGCTCATGAGGAACAATTTATGGATGCGCGACCTTATTCATTGGAAGAAGCAATTGCCATGGAAAAAGCAAAAATACGCGAGGAAAATAGAAAACTGTTGAGAGCTTATCGCGCGTGGGGAACTTTGATGTCAATAGGATCAACCATTTTAGCAATATATGGTGTATATCGTCTGTGTCGGTCATTATTTAAAGATACGACATTGGAAGATATACAATCAGCTAAAGGACTTGATATCAAACAAGCATTGAAGTTAGCGCACGGTTTAAGGAATCAACCCGAAAGAGAACGGCAATATTGGTTGGGTGAAATACGTAGTAAAATGGTTCCCAACGTGGTTACGTGTGTGCTTAGTGATTTGCAGTTTTCTTATGAAGAATTGACAGATAGTTTCGAATTATTTTCGCGCATTAATATTTTATGTATCCCAGAAAATGAAGAAGACATTATATATGATTTGTATTTTGGTAATCTGTTGGACAAAGATGAGGCTAAAGTTGAAATGATTTCTTCAGGATCGCAACCTCTTAACAAGGCTAAGAAGATTGCTGTTGAGAGATTTAGAAAACGCAAAGCTCAAAGTACTGAGATTGTGTCTTCTGGATCCCAACCGCAACAGAAAGCAAAGAAAGTGATTGTGGAACAACCTCTCATGGATATAGCAACCGAAGCATGTAGTGATTTGGCAACCTTACAAGAAGGACGTAGCCTCATGGATAGAAATATCTATAAGTGCACACTAAATGGAGAGCACGTGATGGGAAACGTCATTGTTGTTAAAGGTCAAGTAATTTTGATGCCCTATCATTATGTGCATTGGTTGCAGCTAAAAGGCGTTACGAAGAAGGATCATTTAGGATTGCATTGTATGGGTGCGAATATGACATCAGCTAAATATCAGAGATGTTCTATCAATATGTCTGTGATCTTTGATGATAAAATGAAGTTGACGAAAAACGTACTTAGAATTCAAGATCATGGAGAAGATATAGATGCAGTTCTCATTTATTCAGACCCTCATCATAATATAATGCACAATCATGCTAATATCATTGACAAGTTAATAACGCAAGAGGAAATAGCCTATATCACGCAAGGAACTCAAGCTGTCATGTTTGGCTACAATTCGGATATGAAACGATTTAGCAAATGCGAGAAATTTGTTCAAGAGTTAACTCCCTTTGATACACCGTTACAATTACACATGGATGGTCGAGATTACATGCAGCTACGTTGTGGATATTCGTATAGCGCCGCTTCTGAGAAAGGAGATTGTGGAGCAGCACTATTAGTGCTCAGTCGACGCAATGCTAGGAAATGGATAGGTATGCACGTAGCGGGTAGTAATAATAATGAAGGATATTCAGTTAAGTTGACCCAAGAATTATTGTTGGACCACTTGAAAAAGCTTGAGATTCAAGTGCAAGCCCGTGTAACATCAGCGGAGGTACACAATTTAACAACTGATGGAGCTATTTGTCCTAATGGTGATTTCATTGTTGAAGGAGTCACTAATGTACCATTAAGTGCTGCATCACGCACTAAGATCGAACCATCACTTATATTTGGTGAATTTACTGAAGCCATTACAAAACCAGCATATTTGCAACCTTTTCGAAACGCAGAGGGCAATATAATAGACCCAGCTATGTTAGGTTTAGAAAAAGCTGGAGGACGCCAAGTATTATGTAATACTGACATATTGACTGAAGTTTGCAATCATATGAAACAAAAAATGGACTTGCATTATAATATGTCAATTACTCGGGATACTTACGCTAGAGTAATGACATATGAAGAAGCAGTTCAGGGTGCGAATGATGATTTTATGAAAGCAGTATGTAGGACAACTTCACCTGGTTACCCCTGGAATTCTGATCCCAAACATGCTACTAAATTACCTGGAAAAAGTGCATGGTTGGGTCGAGAAGAACAATTCGATTTCACATCTGATCGGGCTTTAGAACTACGAAAAGCTGTTGAAGTATTGGAAATGGATTGTTTAGCTGGAATTCAGAAAGGAGTAATATGTGCAGATACATTAAAGGATGAGCGACGCCCAATTGCTAAAGTTGATGCTGGAAAAACTCGTTTATTTGCAGCTTGCCCTCAACATTTTGTTATTCTTTTTCGAAAGTATTACCTTGGTTTCAGTGCTTGGGTAATGCATAATCGTATTGATAACGAAGTTGCTGTAGGAACAAACCCATTTAGTTACGATTGGGGTAAGATTGCAAGGAAACTTGCTCTAAAGGGATCAAAAGTGATTGCTGGTGATTTTTCAAATTTTGATGGTTCTCTTAATAGTATGGTGTTGTGGCACGTGTTTGACGTGATTGAATACTGGTATAAGCGCAATGACCCAACATATTGCTTAGATCACTATTTAGTACGCCAAGTTTTATGGACACATATTGTTAATTCAGTACACATATATAAGGATACTATTTATCAATGGACACATTCACAACCTTCCGGTAATCCATTTACTGTAATTATTAACAGTATTTATAACTCAATGATTTTAAGATGTGCATATTTAACCATAGTATCCAAAGGAATTAAAGATAAGAGTATAGATATGTCTTGGTACAGTTTAACTAATTTTGATAAGCATGTTTCTGTTATAACATATGGAGATGATAACTGTTTAAACATATCTGATGATTGCATCTCATTTTTTAATCAGGAAACTTTAACAGCAGCATTGTTAGAGTGGGGACATACATATACCGATGAAGGAAAAACTGGTGAAGTCGTGAAATATCGATCTCTAAAAGAAATTGCTTTCTTAAAACGGAAGTTTGTTTACGATATGGGTTTGAGTAAATGGATGGCTCCACTTGATGAAAGTGTTATTTGGGAAATGTTGAATTGGAAACGTGTGAATGAAATTTCTGCGAAGGAAGCTCTACGTGTAAGTATTACAACTGCCTTGCGTGAAATGGTGTTACATGGAGAAGAAAAATATAATAATTTCGTAAAAACACTTTTAGGAAATAAACGCTTTAGACAGTTAAGATCAAATATAGTATTTCCATCGTATCCCTCTATGTTAGCTCAAATTGAGGACATAGAATATTTTTTAGATTAGGAAGCGCACGTGTGATCTTTTTGTTTCTCAAAAATTATGAGGAGTATAAAGAGAAACAAAATTGCTGCGTGCGTAATAAGGTTTACTATTTAGTATTACTTCCAGGATGCCTTGTTGGCAGCCCCATCCAAATCCAGGAAACTCTTCTAGAATTAATTTGGTTAAGTAGCTTTTTAATTCGAAATAATTTACTTGCTGATACTATAACACAAAATAATACTGATAATGATAAAGAATATATACAAACCCATCGTGAAGAAGTTATTACTTTTCACGATCAGGGTAACACCACTTTTGATCAAGCGATTCCAGAGTTTAGTGACCTGGATGAATCTTATTTATCAATGACGATTGCTCAAGACAAACGTCATGATATTGTGTCCTTCCTTGAACGACCTGTCCGCGTGTGGAGTGGTAATATGAACACTAAAAATACAGTTGGTCAGGTCTTGTGGACTGCATCCTTTCCTAGTGTATTAATAGCAAATACCATGTATAATCAAAAATTGCAAGGTTTTACTGGTTTGCGGGCTGATTTAGAAATCAAGATCCAAGTTAATGCACAAAAATTTCAACAAGGCCGTTTACATTTACAATATATACCATATGCTGATTACTTGAGTAATAAGGTAAAGCTTATTAATGGTTCTTTAGCTGGACGTATTTCTAGTCCGGGTATAGATATTGATATTTGTGGAGGCTCAACTCCTGAATCACGTATAGCTGAAGCAGTATTCCGTGTTCCTTATGTATCACCTCATACTTATTTTAATTTGATTAATGGTGATGGTAAATACGGAATCTTCTATCTATTTGTATATTCCCCTCTGTTGACTGGGGATGGAGGTACAGATAATTGTGAGATAACTGTTTGGTCACGGTTCATTGAACCTAAGGTGGTTTTCCCTACTGGAGCCACTATTGGTTCCGCTATACCTACTAAGACTGCAGAAGTTCAAATTAGAGGTGAAGCAAAACAAATAGCTAAAGAAGGAGTAGTTTCCTCAACTTTAGGTACAGTAGCTGAAGTGTTGAAAATAGGAAAGAAATTGCCAGTTGTTGGTGAATATTTAGCCATACCAGAGTGGATCTGTGATAAAGGATCTGCTATAGCCAAATTATTTGGTTGGTCAAAACCAACTTTGGCTATGGATGTTAAATTGCGAACCAATAATTGTATGACGAATTACAATGGTAAGGATTCCTCCCACAAGATGGCTTTAAGTGCTGATAATGAAATTGATAGTCCTCCTAATATAGCTGGAACTCGTATCGATGAACTAGCTATCTCTTCAATTGTAACTATTCCTACATATTGGAAAACGTTTAGTTGGAATAGTAATGATCAGACGCAAGACCAAATTTTGTGGATTGATAAAGTTTCTGCTGGTAGATTCAGTCAAATTGATGGTACAACTAATGGATATGCCACCACTCCTATGGGCTTCCTTGCAAATATTTTTGCTCAATGGAGAGGTTCTATCAATTACACTTTTAAATTAGTCAAAACAGGTTTTCATTCAGGGCGTTTAAGAGTGTTTTTTGTACCTCAAGGAGTAGCTAGTACATTAAAGGTAGGGAGCGCACCCACTATAGAAATAGAAAAAAATTATCAAATAGTGGTTGATATTGCTGAGAGCGATACCTTTACATTCAATGTACCTTTTGTAGCAACAAAACCATGGTTATCTACATATGGCCCATCAGCGTTTACAGGTTACATAGTTGTAACTGTGCTTAATGAGTTGCGAGCCCCTCCAGTTGTATCAAACAAAATTAATGTTATTGTTGAAGTAGCTGGAGGTTCTGACTTTTCCTTCTCAATGCCCTGTGAGCCATGGGCTTTGACTGGTTCGCCTACGCCATTACCACCTACTATTAAACCAGCTGAAGTACAGATTGCTGGTACTAATGTTGATTTGAACGATGATCAAAAGCGTTCTTTAGTGGATCCTGATTCCATTTCTGTCATAAATCCGTTAGCTAATTGGTCTCCAGAAAGTCATTGCATCGGAGAAAAAATTATGAGTATTAGACAACTCATAAAAAGGGCTAATTGGATAGGGTCCGCAACTATGTTACCAGCTAGCGCTACTGATCAACAATATGCTGTTTTGAACCCTTACGCTAATGCATCTAACCAACAAAATAAGCAAATTGATTATTTATCTTATTTTTCGAATTTATTTGCGTTTTTTAGAGGTGGTGTTAGAATTAAGGTCAATGCAAGTATATTGTATGCTAATGGTAATTTAGGTAACTCAACACCTAATGGTGAATGGTTCACTCCAGCAAATGGAAATCCTTGTTTAAACATTAAAATGTTAAACGCTATTACAACTGATTATATTCAAGTAGCAGCCAATATTAAAAAATTAAATAATATTGTTGGTAAAGTAGGCTTTGCTAATGCTGATGTGTATAATTCTGGTACTTTAAATGCAGCTATGGTAAATAGTTCGAGTACCACAGTAGTGAACCAAGGGGTAGAAGGTATGGTAGAAATCGAGGTACCATACTACAATTCTTCACATTTAACCCCAAGTAATGTACCAAACATTGATGATCCTTTAATTGATATAGATAGTCAAACTACATCTGAAGGTACATATCCCTTACCAATGTTGTTGATTGGTACAAATCCTTTACCTCGTCAATTAAGAATAGCATCAACTGCTGAACATAATGCCCAAGTCTATAAAACTGAAGCGTTAACTTGTAATTTTTATAGAGCAGCATCTGATGATTATTCCTATCATTATTTTATTGGAGTTCCAACAATGATATTCAGACCATCCGTGGCATCTGATTATTATAAACCCCAAAATTAACGGGGGCACATCTATAAGTGTGTCCCTACTACACAACTCTCATTGCAAGCTCGAGAGTGCACTCCGAAAAGAACTTTGAAGCAGAGTCAGCTTGATGGGATCACTGTGTTTTATATATTTAACCCATATACTACGCGTGGGTTATCATACCGTACAACTAATTAAAAGTTGTTTGTTATGTTAACTGGCGTTTTTTAAAAAAAAA

>Fopius arisanus permutotetra-like virus

CGGGGCAAAAGGGACAAAAATTGCTAGGGGGGGCACCAAAACGGACTTGAACTTTTAAAAGTTCGAGTCCGTTTTGGTGCCCCCCCTAGCAATTTTTGTCCCTTTCGCCCTTTCTCATTTTGATTTATTGCTTTACATGATGGATGCCTCAAACCCTGTCTTGTCAAACGACCGCAGAACTATTGGAGAAGTCATGGAGGCGAGCAAGTTGAGGAGGAGACAGAATCTGACTTATATTGCAGAGGTGGCAAGAACATCCCAGCGAGTTGTTCTTCCTGTTGCCTCAGCCCTTCTACCAGTGGAGGAAGTTCGGCAGTTAGTCACTGAATACCGAAACTCCAGAGTGAAGGTCGACGCAAGCAAAGATGGGGACCTCCCTGAAGACATCTTTAGAGTTGTCTACGAGGATCTCCCTGTCTGTGGATTGATGGACGAGCTGGGAGTGCCCTTGCACCCTGCAGGTATCAACAATTCACAAGGAAAACTTTGTGTTAATAGGATAATGGGAGGTGAAGCCCCCTTACCCTTAGGCCTTTCGGAAGTGGCTGAGGTGGCAGGAGGCATGGTTCTAGAGCGAGCCCATGTCTATACAGGCGGAACCTGGAAGGGATTCCTAACCAGACTAGCCCAACAGATGGCGCGCAAAACCGTGCCCCTGCGGAAGACCCTTTCTATAACAGACAAAGAGGTCACGTTTAAAGCAGCACTTGATACACTGGATCAGTATATGCCTAGACAGCCCAATAAAAACTGGCCTGGGGTGCATACTGATCTCTACACTGCGCTGACTGAGGGCATAAAGATAACCTCTAACAGTAGTGCTGGTGCTCCTTATTGGAGAAACAAAGGTGAGTGCATGGACCAGATTCTCGATGTCGGCATACCCATCCTTGTGGAAGCCATCAAAGACAACAAATTGGAGCAGCTTTGGAAGGAAAATCCAGAGCTTTTCATTTGTGAGGTTAAAAACAAGATGGATAGGTACGAGGTTGAGAAGCTCGATTCCAAGACGCGCCCTTATACGTGTGTGCCTGCTCATGTGGCCTTCCTCTTGAGCATGCTGACTCAGGGTTTCCAGGAAACCCTTGAGGTTTTTAGCGAGGAGACGAGCCCTCACTGCAGCAATGCTTATGGTTTTTCGTCAGCCAATGGAGGCATGAATAAACTCCATCGGTGGATGAGGGGAGCAACCAAGAGAGGTAAGGTGATCTGTTATGGCGACGACGCTAAAATCGTCGTCAGGAAGGGAAACGACATCTACTGTGTTGATCCCGATTTCAAACAGATGGATGGTTCTCTGGATGCGGACGACATCGAGCTGACCATTAGATGGGTGCTTAGGCACCTGAGGAAGGATAGTGGAGAAACCCAGGTCCCTCATTTTTGGAAAGCTGTTGCTGATTTGTGGAAGCTAATGGCCACAAATCCCATGTTCGTCGTGGATGGGAAGAAGGTGTATAGAAAAGTAAGCCCTAATGGGCTGATGACAGGAGTCCCCGGGACAACTTTGTTTGATACAGTAAAGTCTGTCCTAGCCTGGAATGCCTACCTGGATAAGTGCGAGATGGACGGAACAGATGTTCTGTCTGAAAGCAATGCAACTGGGTTTATGAAGGCACAGGGTCTTGTCATAAAACCTGGAACGTGGAGCCCCGAGCGGCTCCCTCAGGCAAAACCTGGAGTGTTGATGACGTCCCACAAGTTCTTGGGAGTCCAGATGCTGTGCACCGAGTGGAGGGGGGAACTCTTGATGGTTCCCACCATCCCGGAAAGTGAGGCTCTTCACATGATGGTTGTCCAAAAGGACAGCCCTTTTGAGAAGAATAAATCAAAATTGTCGGCGTCGAGGACTCTTTATGACCGCATGAGAGGCTACATGATAACATGTGGCTTTGCCATTCCTCTTATGCGGGACGCCATTCATAATGTGGTGAATCACCTCCCGCCCGAGGCCATCCTTATGGACGTCCAAACGGCGTCAGGTGAAAAACCTGATCACATATTGCTTCAGGACTTTATGTATCCAGATAGCACCGGATTCCCGTCTGTAGAGTTTTGTTTAGATCTTTATCAGGGACACGAGGACAAGTCCTCGTGGACACAAATTTACCCTGAATTGGTGGGAAAACTTGGCCTCTTCAAGACAGAAGAACGCCAGTGGGGCTCGAAGCTCAAGGCAGTTATTAAGAAGGTTGAGTTTCAACCTGGCTTTCTGACCATGCACTTTGAGGATAAAACCCCTAAGCCTAAAGAGATAGATCCCATTCTTGAGTCCTCTGTGGCTTTTGATAAAGTGGAGCCCCTGCTGGGAGGACAACCAAACAGCAGATCCTTCATCTGTAAGGTTGCAGACTCCAACACCAAAACCCCTGTTAAGAGGATGCCCACTGTGGGCGACCTTGTAATTTCTTATTTGAAGGATATTGGGGGCGTTGGCCAGTTGGGCGTCTGTGTTGAGCGATTAGGAGTTGCCAAACGAGCTGTAGTCAAAGATGCCAGCAAATATGCCTACTACTTGACAGGGGAGGAGGAGGGGGACCTCTTGAGTCTCTACCCTATCCAAACCCCTATGGCAACCATCCAGGATGAGATCGTGGACCACAACAGAGAGGCGCCAATTGGGGAGAGGAAGGTGAAAGCCTTTGACCCCAAAGCCCCTGTGGTGGTGACAAAACCTGACCTCATCTATCTAAATATGCAGATGGTGAGCAACCTTCCCAGAACCCACAGGGTTCTCAAGAATGGTCTTAACGGAGCACAGGCCTTGGAGGCTCTCCAACCCCTGATAACCTCGTTTTATGCTGAGATTAGATGGGAAACTCTCCCTGTTGTACCAAATGCAGACAATCCAGTGGGCGTGCGCCTACTTGTGGACGAGTTTATAATGGAGAGGTTTGACATTAATAAAGTGACGGTAGGCGATTTATGGGCCCCCCCTTTGAGGAGGACCCAGGAACTGGCCCAGGCCTGGAGTAAAAATGCGACCCTGGCCAAACAGTACATTGCCAAAACAATCCTAGAGTTGTGTGGTGTCGTCTGTAAGGAGACTAGCCTCACCACCACAATGTACCCTCCACCCCCGGACTTTGCTGATAGTTGGTATGAGCAAACTCTCCGTTATGAGGATCCCAAGCTGGACCCTCCAATCTCGGACCCCACCACCACCCTCCACCCGTTGAGTGAGCTGATGGAGAACACTCTTTTCTCAGAATTAGCTACTGAGTTCCCTAACGTTGATCCATCACGGATAAGGGCAGCGATCTCCCTAACAAGGAGCAACCCTGAGACTCAGAAACAGGTGGTTAGAAGATGGATTTCAAAGATCAGTCTGAAACAGAACCAGAGGCAGGAGAAGGAAGATCCTGCCACCTCAGTGTCAAAAAGATCCTTGATGACACCGACAGCGCGCCGCAGGCTCAATCGCAACAACGAGCTGCGGAAGAAAAGGAGGCGTCTTGAAAAACAGACACTCCAAGTTGCTCCCCCCTTAAATTGACATTTGAATTTTGTACTACCCACTATCACGATGACTGCCAAAAAGAATATGAACCGAAAGAATGCGAAGAAGCAAGCCAATCGAAATACCCAGAAAGGTAGACCAATGGGCTCAGGAAGCGGCAACGCACCAGTATCCATGGCTGTTGCCGTCCAATCTCGTGCGAATGCGCGTGTCAAGTTGTCCGGAACTGACAGGATCGCCCACCTCGCCAACCTCTCAGGTATGGCAGAGGGTACGGTTGTCATCGACATCCCCCTCACCTGCACAGACATTCCTAGACTTTCAAGTCTGGCAAAAGCCTACCAGAGGGTCCAGTTTTCAAAGCTGGTGTTCAGAGTCGTTCCTATGTCGAGTACCTCAAATAGTGGAGGATATGTCGCCGCGTTCGTCCCTGATGTACGGGATGCTTTTGGCGGCTTACCAAACGCACTCAACAGACTTGTGGCCCAGTCAGGTGCCAAGATTGCGAAGATATGGCAGTCTTGCACCGTCTCGCACCGATGTTTACCAGACAACCTGTATACCAGCATCCCCCCTCTTGGAGAGCTCCGCCTATCTTCCCCCGGAAGATTTGTTATGGCGCTCGATTCGCAGATGCCTGCGAGGGGTGATACGATACCTGTTTCAGTGTACCTGGATTGGACGGTGACACTTTCTGAACCTTCCCTGGAGATAGACTCCCCTATGGCCTCGACCTTGACTGCTGAGGCAAATTTTTACTTGCGGTCTGAGAATGTAGGCCTATGGTATTCCGATACCGGAGGAGGTGATAACCCAACTTTAAAGATACCTGGAATTCAGTTCAACGTGACGTATCGGCTGAGTTCGAAACGATACGTTGACTTCTCCGGTGGTGGAGACACCGGTGTTGAGACGGTAGGTTCCTTCGACAGAGTTCGTTTGGAACGCGACAGTGTCCATGGTGTCACCTTCTTTATCGTTGGTTATGACAACAAAACCATCAAGGAAAAACCTGGCAAAAACCAGTGGTTCATTGAGAAAGGAGATGTCCTAACTCCCGAGGCCCCAAACGTGAAACTGGTCCTGTCCTCTCCCTGTCTGAGCAGATCAACAGGGGTTCAAGAGGAGCAGCCAGGACCATTGTTGACGCAATTGCAGCGCTCGGAGAGTCAATCAAGCATAGAGACAATACCTTCGAGGCAAGAATTGATGAGCTTAGTAAAAGACTTGACAAGGCGATAACCGGTAGTACTCATCTCATCACTGTTGAAGGCGCAGTTGATGCAGTCCCAGCGCAAATTACAGGCCTTCTTACATTTGAGACCTAAAATGGTGAGGATCTGACCCCTCCTCACCCTTCTCGATGTAACATAAATTCCACCCTTCCCCCAGCCGGATGCGCCCTCCAGTATCTAAATTGGAGGGAGTTGAAGTTCAACTCTTCTTTGGGGGTCCACCCCTCCTCACCCTTCTCGATGTAACATAAATTCCAC

>Fopius arisanus narna-like virus

ATCGGGACATTGTTTGCCTAAACGGGGTACGCCCTACATGGCGCACTCAAGAGGTGGGTGGCTTGCCGCGGCGCTTGGACCGAGATTCAGTGTAGTGAATCGAGTCGGCCAGCACCATGCCAGAACGAAACGTTCTGACAGCGACAGCTCCCTGCCCCCTACTACGGACTGCCCTTTCCAGGGTACTCCGTTTAGGCAAACAATGTCCCGATATCCCCGCCTCATTGTCATCGAGGCCCCCATACACGAAGTCGTCATCCAGGCGACTCTCCCAGTTATCCGGGACTCCCCCTACTTGACTAACCCCCCCACACCCATTACCCCTCCCATGGCTGGAAGCCCGGCCGGCGCGATAGCGCCGGCGGTGAAGACCAACAAGCGACGGATGATCGTCGGAAAGCGGCATCAGGCGGCGTGGGTCCCGCTCCGAGAGCGGGACGTAGACCGACTCGGCGCCCCGGGCCATTGCAACGTCGGCCTGATACTCGGCCCTGACAAGGGACCGAACCAGGAACCGGTCGTTGAAGGCCAGGCGCCGGGCGGCCATGTCCGCCTTAGACAGCCGCTTCGAGATGGTAGCCAGGCGGACACCCCGATCCCTCCGGGTGCAGCGCTCTTTTCGGTCAGCCGGACAAAACAGGTCCCGCCACAAGGACTCGAAGGCCACCTCACGGGTGACCTCGTCCTCCAATGGGACAGGAACCATGTTGCCCAGCTGGCCCGAATTCATTGCAGCTGCTTCCCGGGCACGGATGAGACGTGCGGCAGCGCGGCGGTCCGAATTCGAACCGTCCACCCTCCACACGTCCTCGTCGAGGTGTCTCCTGGCACGTTGCTCAACACCGTAGGTGGTGACCCGCAAGGTGGCGCGGAGCCAGTGGCCCCGCCCGCCAGAGGCGAGGGCCTTGGAGAACCCTCCCGGATGAGGAAACCCTGCCCCTCCCACCTCTCGTGGGAGGAGGAGTGGGATCCGATAGTCCCGGTAGCGCTGCACGTACTCCGAGTTCACACTCAGGACGGCCCTGCACATACCGGGAAACAACGGAGACCCCTCCTCCTCAGCGGCATACAGAACGTCTGCAGCCGCTGCGGGCAGGGACACATACACGGGAAGGCCCTCCTTCGAGGGCGCCGGGATAAGGTGCCGGACCGGGATGTCCTGGAGAGCAGTGAGACACGACACGACCTCAACGGCCGCGTCAGTCCACGTCGCTCCACCCAGGTCCTTCCCAGTCAACGGACCGCCGAATCCCAGCGCCTCCAGGGCCCGCGGGTCTCGCGCCAACATAGGAGCGTCAACCGCGAGGCCGGCAGGGAAGGAGATAAACCCCCTCTTCTGCCCGCCCGACAGCGGACGCACCTCGCGGTCCACCAAAAAGGACTTCTCAGCCAGTACGAATGCAGAGTGGGACCGGTAAGACTTGGCCCTGTTGGCCTCGCCGCCGGTGGCCGCAATCAGCTCCTCGTAGGCGGTCGCCTGGCGTTCTGTGAGAGCAGCCGCCAGGTCATCCCCCCTAACGATGGCCGGGGCCAGACCGACGATTCGGCGCACCTCGGCCTCATCGAGACCTCCGTCCTGGGGATCAGTCAGCGCTGCGATGCCACCCAGGCACGCAATGTCGACGATCGCCAGGTTGTAGAGGTTCAAGAGGAACCACGACAGGTTCAGGCCCATCATGCAGCCCCTCATGGAGCGCACAACGATGGGTTTCTCCCCGGGCGCAGCCAGGTCAGGATAGGAGACATCAACGCAACCCAGGATCTTATCTCCCGCTCTTGCGAAGAAAGATTCCTCGGGGATCCCAAGGCCGTGAAGGACCCCATGCCAGAGAGCGAGGATAACGTTCTCAGGCATGAGGTCCGTCGCGGCGGTGAGATCAGCGGAGTACATCCACTGACCGGCCGCACCACGGAGAGACTTGACCACCTGATCGTGGAACTGAGACGCCGCGCCGTCCAACGAACGGCGGCCCGACAGGTCGATCCGGGGGTCCGTCTCCAGGAGTGGCCACAGCACCTTACGCATGGCATCTCCAATGATGCCCCAGTGGGCCGGTGGAGCCGTGACAACTCGGGCTTTGTTCCCGAGCTCCGGCAGAGCCGTTGCCCTCACTGGGATTGCGTAGCGGGTGTCCGCGGCCTCCTCCCGGACCCGGGCCATCGCCGAATCGGTGACCGTCTGGAGGTCCCACTCGACAAAGTACAAACCAGCATAATCCCCCTCGTCGACGTCAAAGAAGCCCGGATCTGTGTCCGGATGCCCGGCGATATCAGCGCGGATGGCGTCAATCTCTGCTCGAGCCAGCTGTTCAAGCTCGGCCCGTTGCCCGCCCTTGGCCCTGCTCATCCCTTCCGAAGCGGAAGAGGAGAACGAGGCGACCACAGAATTCCTGAGCGAGGCTGGTCTGCTTCCAGACCAGGAACTCGCCCAGTGGAAAAGACCGTCGAGGAGTGTCAAAGGCACTCCCGGGTCTGCTGTGGTCAGGACGCGGCGATGTTGTTCCAGGGCAGCAGTGAGAATCGCCTGATCCGCGGGTGGCATGGACCGACCAAGCCGGGCGAGCTGGGCCAGGGCAGTCCTACCGGAGGAGGCGCCCCGGAGGAGGGACCCGAGGCCGTGTTCGAATGCACGGACCGGGAACCCCCGAGGCGCCCCCCGGCGGGACTGCTCCGCGTCCAGAGCCCGGGCGCGGCAGAACTCGGCATATGCCTTGATCTGCCGGATGGTCTCGTTCCGGCCGGAAGACCGCCAGTTGATGGACAGCCACGACCGGACCTTCAGGATCGAGACTGCCATACGGTAGTCTCTCCTGTTACTCAGGTCAAGTCCGCTTCTTTGGAGTCGAGGAATCAGGGAAAGGATACACAAGCGGAAGCTGTCCCAGATGCGGCGGAGGTCGCGGCAGTCTGCCGCGGCCCCGCACCCCAGAGGGCAGCCACCCGGTGGATCCCGCGAGTGGCCCCGCTTCCGAGGTCGCTCGCCTGCTGGTCCCCCCCCCCCCCCTCCCGGGCGGGGGGGGGGGGGTGGGGGGGGGGGGGGGGGGGAGGGAAGTCAAGGGGGAGCAGGCAGGGTCTCCCCTGCCTGGCGTGTATTTTCTAAGGGGATGAGGCTCAGAGCCTCGGGGATGTCTGGAGACGGAACAGGCTGGTATGCCTGCCGCCTCCAGTCGGGACAAATAATTGCCCGTAACCCCGCGGGGGAACGCGTCTTTCCTCAGGTTACTGAGGAGGCGCGCTGCCGCCGCGGGGGGAGTCACAGCTGTGGCGACGGGCTCAGGTAGGTTGGGAAGTTTCCACTTCCCGGACCTCCCAAGTCGTGCACTAGCC
